# *In silico* development and characterization of tri-nucleotide simple sequence repeat markers in hazelnut (*Corylus avellana* L.)

**DOI:** 10.1371/journal.pone.0178061

**Published:** 2017-05-22

**Authors:** Gehendra Bhattarai, Shawn A. Mehlenbacher

**Affiliations:** Department of Horticulture, Oregon State University, 4017 ALS Building, Corvallis, Oregon, United States of America; University of Helsinki, FINLAND

## Abstract

Plant genomes are now sequenced rapidly and inexpensively. *In silico* approaches allow efficient development of simple sequence repeat markers, also known as microsatellite markers, from these sequences. A search of the genome sequence of 'Jefferson' hazelnut (*Corylus avellana* L.) identified 8,708 tri-nucleotide simple sequence repeats with at least five repeat units, and stepwise removal of the less promising sequences led to the development of 150 polymorphic markers. Fragments in the 'Jefferson' sequence containing tri-nucleotide repeats were used as references and aligned with genomic sequences from seven other cultivars. Following *in silico* alignment, sequences that showed variation in number of repeat units were selected and primer pairs were designed for 243 of them. Screening on agarose gels identified 173 as polymorphic. Removal of duplicate and previously published sequences reduced the number to 150, for which fluorescent primers and capillary electrophoresis were used for amplicon sizing. These were characterized using 50 diverse hazelnut accessions. Of the 150, 132 generated the expected one or two alleles per accession while 18 amplified more than two amplicons in at least one accession. Diversity parameters of the 132 marker loci averaged 4.73 for number of alleles, 0.51 for expected heterozygosity (H_e_), 0.49 for observed heterozygosity (H_o_), 0.46 for polymorphism information content (PIC), and 0.04 for frequency of null alleles. The clustering of the 50 accessions in a dendrogram constructed from the 150 markers confirmed the wide genetic diversity and presence of three of the four major geographic groups: Central European, Black Sea, and Spanish-Italian. In the mapping population, 105 loci segregated, of which 101 were assigned to a linkage group (LG), with positions well-dispersed across all 11 LGs. These new markers will be useful for cultivar fingerprinting, diversity studies, genome comparisons, mapping, and alignment of the linkage map with the genome sequence and physical map.

## Introduction

European hazelnut (*Corylus avellana* L.), one of the world's major nut crops, is a diploid (2n = 2x = 22) that belongs to the Betulaceae. Between 1993 and 2013, the USA produced ~4% of the world crop and ranked third in world production, with around 99% grown in Oregon's Willamette Valley. In the decade 2005–2014, the leading country, Turkey, produced 67.2% of the world crop, followed by Italy at 12.6%, the United States at 4.0%, Azerbaijan at 3.4%, and Georgia at 3.1% (www.fao.org/faostat/en/#compare, accessed 20 Jan. 2017). Most of the world's cultivars originated as selections from local wild populations. All are monoecious and wind-pollinated, highly heterozygous, and have been clonally propagated for decades or even centuries. Thus, one could say that there are several different sites of domestication. Based on simple sequence repeat (SSR) markers, also known as microsatellite markers, most cultivars have been assigned to one of four major geographical groups: Central European, Black Sea, English or Spanish-Italian [1,2].

SSRs are DNA segments made up of tandem repeat motifs 1–6 nucleotides in length. Primers can be designed from conserved regions that flank the repeat, and fragments amplified using the polymerase chain reaction (PCR). SSRs are ubiquitous in eukaryotic genomes. Their evolution is not completely understood but DNA replication slippage [3,4], mutation, unequal crossing-over and gene conversion [5] have been offered as explanations for motif length variation.

DNA markers are signposts or flags at specific positions on the linkage map. DNA markers are not affected by growth stage or environment, and hence, can be employed at any plant growth stage, and used for indirect selection of alleles at loci tightly linked to them. Compared to morphological and biochemical markers, DNA markers are more abundant. Several types of DNA markers are available to detect genetic variation in plant populations, and these markers are indispensable tools for finding associations between genotype and phenotype. SSRs are the genetic markers of choice for many applications, due to their relative abundance, extensive genome coverage, high reproducibility, high level of polymorphism with multiple alleles, co-dominant inheritance, interspecific and inter-generic transferability, amenability to automated high-throughput genotyping, and ease of sharing among laboratories [6]. They are neutral in that there is generally no effect on phenotype [7] and are transferable across hazelnut species and related genera in the Betulaceae [8,9,10]. SSR markers have been widely used in plant genetic studies and have practical applications in plant breeding and the management of plant collections. Applications include cultivar identification, parentage and genetic diversity analyses, identification of duplicates in collections, marker-assisted selection (MAS), quantitative trait locus (QTL) analysis and linkage mapping [11,12,13]. SSR markers serve as anchor loci on the linkage map and create the framework for a physical map by aligning the linkage map with BAC contigs [14,15,16]. Moreover, SSRs have been used in comparisons of genome structure and synteny between related species, including diploids and polyploids, and functional, evolutionary and comparative genomic studies [14,17,18,19].

Around 350 polymorphic SSR loci have been developed in *Corylus avellana* from genomic DNA libraries enriched for specific repeats [8,9,20,21], inter simple sequence repeat (ISSR) markers and flanking sequences [22], BAC sequences [15], transcriptome sequences [23] and searches of public databases of genome, transcriptome and expressed sequence tag (EST) sequences [24,10]. Early marker-development efforts used genomic libraries enriched for fragments containing SSRs, but the high cost and the time involved are major disadvantages of this approach [25]. With the advent of next-generation sequencing technology for whole genomes [26] and transcriptomes, and the resequencing of the genomes of additional accessions at low coverage, it is now feasible to develop new polymorphic SSR markers for plant species in a short time at relatively low cost. Using genomic sequences, SSR markers have been developed for dwarf bulrush (*Typha minima*) [27], *Sorghum bicolor* [28] and foxtail millet (*Setaria italica*) [19]. From transcriptome or EST sequences, SSRs have been developed for garden rose (*Rosa* sp.) [29], mung bean (*Vigna radiate*) [30], hemp (*Cannabis sativa*) [31], foxtail millet (*Setaria italica*) [32], barley (*Hordeum vulgare*) [33] and *Chrysanthemum nankingense* [34].

The genome of 'Jefferson' hazelnut was sequenced at 93× coverage using the Illumina HiSeq 2000 platform and assembled into contigs and scaffolds with a total length of 345 MB, representing 91% of the genome [35]. An additional seven cultivars were sequenced at ~20× coverage [35]. We used these genomic sequences to rapidly develop new polymorphic tri-nucleotide SSR markers, a type that is often polymorphic and easy to score, and of which few have been developed to date. Further, we characterized the new markers, studied segregation and mapped them in our reference population, and used them to study diversity in 50 hazelnut accessions

## Materials and methods

### *In silico* SSR identification

Genome sequences of 'Jefferson' and seven other *C*. *avellana* cultivars ('Barcelona', 'Ratoli', 'Tonda Gentile delle Langhe', 'Tonda di Giffoni', 'Daviana', 'Hall's Giant' and 'Tombul') at lower coverage [35] were used. SSR motifs were identified in the 'Jefferson' genome sequence using the MISA (MIcroSAtellite) Identification Tool [33], which identifies only perfect repeats, and sorted according to repeat motif length into four files (tri-, tetra-, penta- and hexa-repeats). For the di-, tri-, tetra-, penta- and hexa- repeats, the search criteria specified minimum numbers of repeats as 6, 5, 4, 4 and 4, respectively. This study focused on tri-nucleotide repeat motifs with ≥ 5 repeats. Di-repeats were not pursued as hundreds had been previously developed, and repeat motifs containing only A's and T's were not pursued as in our experience they are difficult to score. Unique 'Jefferson' fragments containing SSRs were trimmed, retaining 250 bp on either side of the repeat motif. Paired-end Illumina genome sequences from the seven other accessions were pooled using the "concatenate" command, and then aligned with the 'Jefferson' sequences as the references using the MAQ program [36]. The aligned sequences were visualized using Tablet software [37] and the aligned reads inspected for variation in number of repeat units but conserved flanking regions. Tablet software displayed the reference sequence at the top, with the aligned reads from the seven cultivars shown in rows below it, but the identity of the cultivar for each aligned read was not indicated. Each nucleotide was shown with a different color, allowing efficient identification of SSRs that showed variation in number of repeats. Repeats near the ends of fragments were not pursued if the length of the flanking region was insufficient or the sequence was unsuitable for primer design (e.g. very low G-C content). Although most fragments were 500 bp in length, fragments as short at 400 bp were used, but fragments <400 bp were discarded. After alignment in Tablet, they were visually inspected for variation in number of repeats but conserved flanking regions. Loci were classified as "not polymorphic", "clearly polymorphic" or "slightly polymorphic", the latter class for aligned sequences with <2% of the reads showing variation in the number of repeats. Tri-nucleotide repeat SSRs that were rated as "clearly polymorphic" and met these criteria were targeted. The programs Websat [38] and Primer 3 [39] were used to design forward and reverse primers with lengths of 18 to 27 bp, annealing temperature of 60°C, and a range of expected product sizes of 90–400 bp. The wide size range was intended to facilitate multiplexing of PCR products. Non-fluorescent forward and reverse primers were ordered from Integrated DNA Technologies (Coralville, IA).

### Plant material

A diversity panel of 48 hazelnut accessions (Tables 1 & 2) plus the two parents of the reference mapping population were used to characterize the new SSR markers. The same 50 accessions were used in previous characterization studies [21,22]. Of the accessions, 24 (Table 1) were used to validate SSR polymorphism on agarose gels after their *in silico* identification. These accessions represent the wide geographic range and phenotypic diversity of *Corylus avellana* and were chosen from those previously investigated [2] to increase the likelihood of identifying polymorphic marker loci.

**Table 1:** Hazelnut accessions used to screen for polymorphism on agarose gels and to characterize tri-nucleotide simple sequence repeat markers mined from genome sequences.

Accession	Designation	Origin	Source
Ala Kieri COR187	PI 557080	Finland	Lappa, Finland (seeds)
Albania 55	PI 617207	Albania	Cajup, Albania (seeds)
Aurea	PI 557050	France	Morton Arboretum, Lisle, IL, USA (scions)
Barcelona	PI 557037	Spain	Oregon nursery (scions)
Bergeri	PI 557114	Belgium-Luttich	ISF Rome, Italy (scions)
Casina	PI 557033	Spain-Asturias	Q.B. Zielinski from Spain (scions)
Cosford	PI 557039	England-Reading	NYAES, Geneva, NY (scions)
Cutleaf	PI 557306	England	Arnold Arboretum, Boston, MA (scions)
DuChilly	PI 557099	England	OSU Entomology Farm, OR, USA (scions)
Fusco Rubra	PI 557047	Germany	Morton Arboretum, Lisle, IL, USA (scions)
Gasaway	PI 557042	USA-Washington	Orchard in Washington, USA (scions)
Hall's Giant	PI 557027	Germany/France	Oregon State Univ. Entomology Farm, OR, USA
Imperiale de Trebizonde	PI 271105	Turkey	Q.B. Zielinski from France (scions)
Negret	PI 270340	Spain-Tarragona	Q.B. Zielinski from Spain (scions)
OSU 408.040	PI 617266	Univ. Minnesota	Univ. Minnesota Horticulture, MN, USA (seeds)
OSU 495.049	PI 557421.2	Russia-Southern	VIR, southern Russia (seeds)
OSU 681.078	PI 634204	Russia-Moscow	Moscow, Russia (seeds)
Palaz	PI 304632	Turkey-Ordu	Q.B. Zielinski from Greece (scions)
Pendula	PI 557048	France	Arnold Arboretum, Boston, MA, USA (scions)
Ratoli	PI 557167	Spain-Tarragona	IRTA Mas Bove, Reus, Spain (scions)
Rote Zellernuss	PI 271280	Netherlands	Q.B. Zielinski from Netherlands (scions)
Tombul Ghiaghli	PI 304634	Turkey	Q.B. Zielinski from Greece (scions)
Tonda di Giffoni	PI 296207	Italy-Campania	Q.B. Zielinski from Italy (scions)
Tonda Gentile delle Langhe	PI 557035	Italy-Piemonte	Univ. di Torino, Italy (scions)

**Table 2:** Additional hazelnut accessions used to characterize tri-nucleotide simple sequence repeat markers mined from genome sequences.

Accession	Designation	Origin	Source
Alli	R72.02	Estonia	Polli, Estonia (scions)
Artellet	PI 557108	Spain-Tarragona	IRTA Mas Bove, Reus, Spain (scions)
B-3	PI 557122	Macedonia	Skopje, Macedonia (scions)
Barcelloner Zeller	PI 557156	Spain	Faversham, England, UK (scions)
Buttner's Zeller	PI 557094	Germany-Landsberg	Faversham, England, UK (scions)
Contorta	PI 557049	England	Arnold Arboretum, Boston, MA, USA (scions)
Des Anglais	PI 557423	unknown	INRA, Bordeaux, France (scions)
Early Long Zeller	PI 557090	Germany	Faversham, England, UK (scions)
Gem	PI 557029	USA-Washington	Orchard in Oregon, USA (scions)
Gunslebert	PI 557191	Germany-Gunsleben	INRA Bordeaux, France (scions)
Gustav's Zeller	PI 557085	Germany-Landsberg	Faversham, England, UK (scions)
Iannusa Racinante	PI 557183	Italy-Sicily	Univ. di Torino, Italy (scions)
Mortarella	PI 339723	Italy-Campania	Q.B. Zielinski from Italy (scions)
OSU 26.072	PI 323961	Russia-N. Caucasus	North Caucasus, Russia (seeds)
OSU 54.039	PI 557060	Turkey-Giresun/Ordu	Giresun, Turkey (seeds)
OSU 556.027	PI 617269	Turkey-Istanbul	Istanbul market, Turkey (seeds)
OSU 759.010	OSU 759.016	Georgia	Ozurgeti, Georgia (scions)
Pellicule Rouge	PI 271110	France	Q.B. Zielinski from France (scions)
Römische Nuss	PI 557171	unknown	Hermansverk, Norway (scions)
Sant Jaume	PI 557103	Spain-Tarragona	IRTA Mas Bove, Reus, Spain (scions)
Simon	PI 557166	Spain-Tarragona	IRTA Mas Bove, Reus, Spain (scions)
Tapparona di San	PI 617239	Italy-Liguria	Univ. di Torino, Italy (scions)
Colombano Cortemoli			
Tonda Bianca	PI 296206	Italy-Campania	Q.B. Zielinski from Italy (scions)
Tonda Romana	PI 557025	Italy-Lazio	ISF Rome, Italy (scions)
OSU 252.146	—	USA-Oregon State Univ.	Parent of mapping population 93001
OSU 414.062	—	USA-Oregon State Univ.	Parent of mapping population 93001

### DNA extraction and amplification for polymorphism screening

For DNA extraction, 2–4 young leaves were collected during the spring from the USDA-ARS National Clonal Germplasm Repository (NCGR) and the Smith Horticultural Research Farm of Oregon State University (OSU) in Corvallis. DNA was extracted following the method of Lunde et al. [40] without RNAase treatment. The DNA was quantified using ultra-violet spectrophotometry with a BioTek Synergy 2 Multi-Mode Reader with a Take 3 microplate reader, the data was analyzed with Gen5 software (Biotek Instruments, Winooski, VT), and the DNA diluted with TE buffer to a concentration of 20 ng·μl^-1^. The polymerase chain reaction (PCR) was performed with each pair of primers using DNA of 24 accessions in the diversity panel (Table 1). PCRs were done in 10 μl volumes containing 0.3 μM each of forward and reverse primers, 1× Biolase NH_4_ reaction buffer, 2 mM MgCl_2_, 200 μM each of dATP, dCTP, dGTP, and dTTP, 20 ng template DNA, and 0.25 units of Biolase DNA polymerase (Bioline USA Inc., Taunton, MA). PCR amplification was performed in GeneAmp PCR system 9700 thermal cyclers (Applied Biosystems, Foster City, CA) in 96-well plates with denaturation at 95°C for 5 minutes followed by 40 cycles of 94°C for 40 seconds, 60°C for 40 seconds, 72°C for 40 seconds, extension at 72°C for 7 minutes, and a final infinite hold at 4°C. The PCR products were separated by electrophoresis on 3% agarose gels in TBE buffer at 90V for 3.5 h, stained for 30 min in ethidium bromide and then destained in water for 25 minutes. Gels were then photographed under UV light using a BioDoc-It® Imaging System (UVP, Upland, CA). The gel images of the PCR products were visually inspected for size polymorphism among the 24 genotypes.

### Genotyping at polymorphic SSR marker loci

For tri-nucleotide repeat SSR loci showing polymorphism on agarose gels, fluorescent forward primers labeled with 6FAM or HEX were ordered from Integrated DNA Technologies (Coralville, IA) and fluorescent forward primers labeled with NED were ordered from Applied Biosystems (Foster City, CA). DNA from 48 diverse accessions and the two parents was amplified with the fluorescent forward and non-fluorescent reverse primers. The use of fluorescent primers and PCR products of different size ranges allowed efficient post-PCR multiplexing of 5–7 primer pairs in a single well. For multiplexing, two μl of the PCR products from each primer pair were mixed and diluted with water to make a final volume of 150 μl. An aliquot of 1–1.5 μl of the multiplex was submitted to the Core Labs of OSU’s Center for Genome Research and Biocomputing (CGRB) for fragment sizing by capillary electrophoresis on an ABI 3730 (Life Technologies, Carlsbad, CA) with ROX-500 as the size standard. Allele sizes were determined using GeneMapper^®^ software (Life Technologies, Carlsbad, CA) and recorded in a spreadsheet. PCR amplification and capillary electrophoresis were repeated if the initial PCR failed or the result was ambiguous.

### Characterization of polymorphic SSRs

For primer pairs that showed the expected one or two PCR products in all cultivars, PowerMarker software [41] was used to calculate the number of alleles (n), observed heterozygosity (*H*_*o*_), expected heterozygosity (*H*_*e*_) and polymorphism information content (*PIC*) for each locus. Observed heterozygosity is the frequency of heterozygous genotypes per locus, and calculated as number of heterozygous genotypes divided by total number of genotypes at each locus. Expected heterozygosity estimates the probability that a randomly chosen individual is heterozygous at a locus and is calculated as $He=1-\sum_{i=1}^{n}p_{i}^{2}$, where frequency *p*_*i*_ is the frequency of the *i*^*th*^ allele and *n* is the number of alleles [42]. The polymorphism information content value is a measure of marker usefulness or informativeness and is calculated as $PIC=1-(\sum_{i=1}^{n}p_{i}^{2})-\sum_{i=1}^{n-1}\sum_{j=i+1}^{n}2p_{i}^{2}p_{j}^{2}$, where *p*_*i*_ is the frequency of the *i*^*th*^ allele, *p*_*j*_ is the frequency of the *j*^*th*^ allele, and *n* is the number of alleles [43]. The frequency of null alleles was calculated with Cervus software (www.fieldgenetics.com), which uses the formula of Kalinowski and Taper [44].

The genotype data for the 50 accessions at all new SSR loci were converted to binary format using an Excel macro [45] and then used in cluster analysis. A frequency-based distance matrix was computed and then dendrograms were constructed using PowerMarker using the neighbor joining (NJ) and unweighted pair-group method using arithmetic averages (UPGMA) algorithms. The resulting dendrograms were visualized using MEGA6 software [46] and compared.

To determine the number of new SSRs that were in genes, the SSR sequences were used in a BLAST search against the annotated transcriptome of 'Jefferson' hazelnut and counts recorded for sequences with matches of 100% and ≥ 95%.

### Segregation and linkage map construction

For loci at which the parent genotypes of the mapping population (OSU 252.146 × OSU 414.062) [47] predicted segregation, all 138 available seedlings were genotyped as described above. Each allele size was scored as present or absent in each seedling, scores were tallied, and the expected ratio (1:1 or 1:1:1:1 or 1:2:1) was noted. Scores for the new SSR markers were added to those for previously mapped Random Amplified Polymorphic DNA (RAPD) and SSR markers [23], and the data were imported into JoinMap 4.1 (Kyazma, Wageningen, Netherlands). A two-way pseudo-testcross analysis and the BC1 function were used to construct the maps with the maximum likelihood method and distances in Haldane units (cM). Maps for each linkage group were constructed separately, with a median LOD score of 12 (range 9 to 15). Markers that clustered loosely with the others and fell out at LOD scores <9 were removed. Markers present in repulsion phase were included by creating “dummy variables”, whose use allowed the merger of the repulsion phase and coupling phase markers and generation of a single map for each linkage group in each parent. The JoinMap output was inspected for "Fit and Stress", and markers removed in stepwise fashion until the "Nearest Neighbor Stress" value for all markers was less than an arbitrarily set value of 7.60. The "Nearest Neighbor Fit (cM)" values were also inspected, as high values indicate blocks of markers that fit poorly with adjacent markers. The length of the gaps between markers was also inspected, with gaps of >20 cM considered suspicious.

## Results

A search of 333,492 'Jefferson' genome sequences identified 167,048 SSRs with repeat motifs of one to eight bp. Mono-nucleotide repeats (≥ 10 repeat units) were most abundant and comprised 69.31% of the total, followed by di- (19.92%), tri- (5.21%), tetra- (3.83%), penta- (1.05%), hexa- (0.55%), hepta- (0.11%), and octa-nucleotide (0.001%) repeats. The identified tri-nucleotide repeats were investigated further. Of the 8,708 tri-nucleotide SSRs with five or more repeat units, those containing only A and T were the most common (43.1%), followed by AAG/CTT (28.4%) and ATC/TAG (8.4%). The number of sequences investigated was reduced in a stepwise manner, resulting in the final set of 150 polymorphic tri-nucleotide repeat SSR marker loci. Removal of 3,756 sequences containing only A and T reduced the number to 4,952. Removal of short fragments (< 400 bp) and fragments whose repeats were at or near the end (so primer design was not possible) further reduced the number to 1,056 sequences. Of these, the 'Jefferson' reference had no aligned reads from the other seven cultivars for 33 sequences, which we attribute to the low genome coverage of the seven re-sequenced cultivars. For 90 'Jefferson' sequences, the sequences of the other seven cultivars aligned poorly. Of the remaining sequences aligned with Tablet, 597 showed no polymorphism, 93 showed only slight polymorphism, and 243 showed clear variation in the number of repeat units but conserved flanking sequences. Sequences were scored as "slightly polymorphic" if <2% of the reads showed variation in the number of repeats. Because of the large number with clear polymorphism, the "slightly polymorphic" repeat-containing sequences were not pursued. Of the 243 for which primers were designed, 173 appeared polymorphic when PCR product sizes were inspected on agarose gels. Of these 173 sequences, comparisons identified 23 as identical to others. One was identical to an ISSR marker sequence, one to a cloned gene sequence, 14 to sequences in the hazelnut transcriptome [10,22,23], and seven were the reverse complements of others identified in this study. Identity in the first three categories was detected using a BLASTx search of sequences deposited in NCBI (blast.ncbi.nlm.nih.gov/Blast.cgi). Fluorescent forward primers were ordered for the 150 polymorphic tri-nucleotide markers, 57 labeled with 6FAM, 69 with HEX, and 24 with NED. Allele sizes at the 150 new SSR marker loci for the 48 accessions plus the two parents of the mapping population are presented (S3 and S4 Tables). At 132 marker loci, all of the 50 accessions had the expected one or two alleles, but at 18 marker loci, one or more accession had three or four PCR products. For the loci with only one or two products, the allele size data for 50 accessions provided estimates for characterization (S2 Table). A total of 624 alleles was identified at these 132 marker loci. The number of alleles per locus (n) ranged from 2 to 13 with an average of 4.73. Expected heterozygosity (*H*_*e*_) ranged from 0.039 to 0.845 with a mean of 0.509, and observed heterozygosity (*H*_*o*_) ranged from 0.040 to 0.900 with a mean of 0.486. The polymorphism information content (*PIC*) values ranged from 0.038 to 0.825 and averaged 0.457. The loci with the highest PIC values were GB823 and GB916 with *PIC* > 0.80. In contrast, GB944 had the lowest *PIC* value (0.038) and only two alleles. The estimated frequency of null alleles [F(null)] ranged from -0.154 to 0.854 and averaged 0.042. Estimates of the frequency of null alleles were very high (> 0.25) at five loci (GB 858, GB821, GB902, GB393 and GB856).

A total of 105 loci segregated in the mapping population, of which 101 were placed on the linkage map (S2 Table). Four loci (GB333, GB354, GB824, GB887) could not be assigned to a LG, all of which showed poor fit to the expected ratios of 1:1 or 1:2:1. Of the 105 segregating loci, 22 were tested for fit to a 1:1 ratio from the female parent, 37 to a 1:1 ratio from the male parent, 17 to a 1:1:1:1 ratio, and 28 to a 1:2:1 ratio (S2 Table). At GB876, the female parent had three amplicons and the male parent had four amplicons, but amplicons 176 from the female parent and 178 from the male parent showed 1:1 segregation and were placed on the map. Segregation ratios showed poor fit to Mendelian expectations (P < 0.05) at 17 loci, of which 11 showed very poor fit (P<0.01). Of the 17, 13 were assigned to LGs, and the other four were not. The initial map of the female parent consisted of 11 distinct linkage groups while the male parent had 10 distinct groups, with LG2 and LG7 merged together. Following the methods of Colburn et al. [23], we separated LG2 and LG7 and present the map as 11 pairs with loci common to female and male parents connected by lines (S1 Fig). At several loci that map to LG11, including GB315, GB343, and GB357, one allele from the male parent is transmitted at a much higher frequency than the other. For markers that did not segregate in the reference mapping population, we expect future research in alternate mapping populations to assign them to LGs. The newly developed markers were assigned to all 11 LGs but were not distributed uniformly (S1 Fig). LG9 presented challenges for mapping. The map for LG9S shows a 15 cM gap between B732 and BR414, with high "Nearest Neighbor Fit" values for the markers flanking the gap. The map of LG9R showed two large gaps: 22 cM between GB838 and KG845, and 33 cM between markers BR410 and GB318. Alignment of LG9S and LG9R showed that homologues of markers in the upper part of LG9S were in the middle segment of LG9R, possibly indicating errors in assembly of these large pieces or cytogenetic abnormalities.

**Fig 1:**
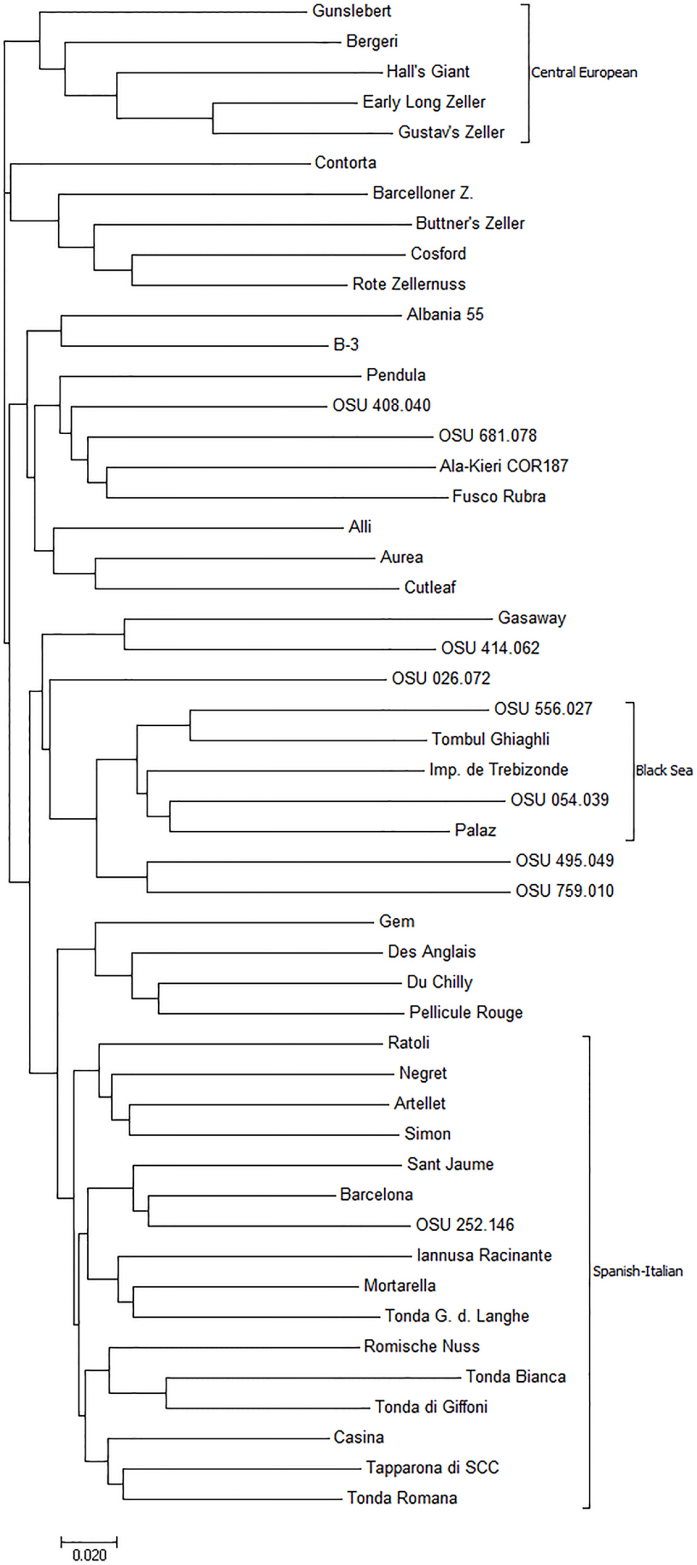
Neighbor-joining dendrogram of 50 hazelnut accessions constructed from binary data for 150 new simple sequence repeat markers.

Dendrograms constructed using NJ and UPGMA for the 50 genotypes were very similar, and only the NJ dendrogram is presented (Fig 1). The dendrogram confirms the wide genetic diversity in hazelnuts, and shows three (Central European, Black Sea and Spanish-Italian) of the four main geographic groups observed in previous studies [1,2,21,23], while the English cultivars 'Cosford' and 'DuChilly' (syn. 'Kentish Cob') appeared in different branches in the dendrogram. The groupings are not tight, and many accessions of different origin appear outside of the major groups. 'Hall's Giant', 'Gunslebert', 'Early Long Zeller' and 'Gustav's Zeller', all of German origin, were placed in the Central European cluster. 'Palaz', 'Imperiale de Trebizonde', 'Tombul Ghiaghli', OSU 054.039 and OSU 556.027, all of Turkish origin, were placed in the Black Sea group. Sixteen accessions were placed in the Spanish-Italian group at the bottom of the tree.

A BLAST search of the 150 SSR sequences against the 'Jefferson' full-length annotated transcriptome showed that 48 sequences showed 100% match and 107 showed ≥ 95% match to transcriptome sequences, including multiple hits to the same gene sequence. When the multiple hits were removed, there were 44 sequences showing 100% match and 83 sequences showing ≥ 95% match to transcriptome sequences.

## Discussion

An *in silico* approach is efficient in terms of both the cost and time involved for developing simple sequence repeat markers for organisms with draft genome sequences. The high throughput and low cost per sample of Illumina sequencing compensates for the short read lengths, which have steadily increased in recent years. Newer technologies, including those of Pacific Biosciences, currently allow longer read lengths at reasonable cost, but were not available at the time that Rowley [35] sequenced the 'Jefferson' hazelnut genome. We used a stepwise approach to develop 150 new polymorphic tri-nucleotide simple sequence repeat markers. Post-PCR multiplexing of products of different sizes and different fluorescent tags reduced genotyping costs, as several fragments were simultaneously separated in a single sizing run. Uniform fluorescence intensity is desired for accurate allele size calling, with dilution factors adjusted to achieve this. "Bleeding" was encountered, where peaks appeared on the capillary electrophoresis output for one dye but the products had actually been tagged with a different dye. Thus, the PCR product sizes of the SSRs in a multiplex set should not overlap in size. In addition, several markers showed stutter bands which made scoring difficult. Stuttering results from slippage of Taq DNA polymerase during PCR and is generally less frequent with longer repeat motifs. Di-nucleotide repeats were abundant in the 'Jefferson' genome but were not used in this study as large numbers of these markers had been previously developed [8,9,20,21,22,24], and stuttering is a more common problem with di-nucleotide repeats [12]. Similarly, mononucleotides were highly abundant but not pursued as they tend to be difficult to score, and they were not needed as we have many SSRs with higher repeat lengths. Colburn et al. [23] developed 111 polymorphic simple sequence repeat markers from hazelnut transcriptome sequences, of which 96 have three-base repeats. The 150 new tri-nucleotide repeat polymorphic simple sequence repeat markers developed from the 'Jefferson' hazelnut genome sequence substantially increase the number of these useful markers.

SSR markers developed in *Corylus avellana* have a high rate of transferability to other *Corylus* species and some transferability to related genera [10,24], and thus these new markers will be useful not only in *C*. *avellana* but also in relatives and allow comparative mapping with *Betula*, *Alnus* and other genera in the Betulaceae. Similar results on transferability have been reported for markers developed in barley [33], foxtail millet [19,32], sugarcane [13], peanut [48], chestnut [49], *Prunus* [50] and the Euphorbiaceae [51].

Several markers showed alleles that only occur in one individual, as reported by Gökirmak et al. [2] who studied 21 SSR markers in 198 unique accessions. Unique alleles are evident in the histograms (S2 Fig) and tables of allele sizes in the 50 accessions (S3 and S4 Tables). These alleles, called private or unique, were confirmed in our study by repeating PCR and genotyping.

Null alleles, which fail to amplify with PCR, are the result of SNPs or other mutations in either one or both primer binding site sequences. These binding sites are generally conserved in genomes. The presence of null alleles leads to errors in pedigree and segregation analysis [52,53]. The segregation ratios at GB346 indicate presence of a null allele in the female parent (S2 Table). The null allele frequency was low at most loci (mean = 0.04), but higher at others, including five with values > 0.25.

The NJ dendrogram showed accessions clustered according to their geographic origin into three (central European, Black Sea, and Spanish-Italian) of the four main groups reported in previous studies (1,2). Understanding the genetic variation present in collections of cultivars and advanced selections, and using a broad genetic base in breeding, are very important for continued genetic improvement, especially for cross-pollinated and clonal crop species where inbreeding must be avoided. A subset of these new polymorphic SSR markers will be useful for studies of genetic diversity and its efficient use in breeding. The addition to the map of thousands of new markers from genotyping-by-sequencing [54] would be straightforward. A dense linkage map will facilitate the study of qualitative traits such as new sources of resistance to eastern filbert blight, as well as the placement of quantitative trait loci (QTL) on the map [55]. A major attraction of simple sequence repeats is that they can serve as anchor loci on linkage maps. They are often polymorphic in several full-sib families and map to the same location, and thus are powerful for aligning the maps of the parents of the different families. Four new SSRs are of particular interest for studying sources of resistance to eastern filbert blight. GB917 was assigned to LG6 close to a mapped resistance locus from 'Gasaway', 'Culpla', 'Crvenje', OSU 408.040 and OSU 495.072 [56,57]. GB822 was placed on LG7 close to a mapped resistance locus from 'Ratoli' [58], and GB329 and GB829 on LG2 were close to a mapped resistance locus from Georgian OSU 759.010 [59]. Additionally, GB309 lies on LG5 near the S-locus that controls pollen-stigma incompatibility.

Additional markers could be developed from the SSR-containing fragments identified in this study, in particular those with longer repeat motifs (tetra-, penta- and hexa-), the >19,000 di-repeats that were identified but not pursued, and 93 tri-nucleotide repeat SSRs classified as "slightly polymorphic". Fragments <400 bp and those that contained SSR repeat motifs with flanking sequences insufficient for primer design, identified in this study, could be pursued in the future, especially as the genome sequence assembly is improved.

The 150 genomic tri-nucleotide repeat SSRs developed in this study, along with the >350 SSRs developed earlier, will facilitate further research in the genetics of hazelnut and related species, particularly in cultivar fingerprinting, studies of genetic diversity, qualitative and quantitative trait locus mapping, marker-assisted selection, and the fine mapping and cloning of genes. The mapped SSR markers will allow alignment of the linkage map with genome sequences and the physical map of BAC contigs and genome sequences.

## Supporting information

S1 Fig Linkage maps of the hazelnut reference mapping population (OSU 252.146 x OSU 414.062). The new tri-nucleotide simple sequence repeat markers are indicated by * and bold font.
(PDF)

S2 Fig Histograms showing allele frequencies at 150 new tri-nucleotide simple sequence repeat marker loci in hazelnut. (PDF)

S1 Table Characteristics and primer sequences of 150 new simple sequence repeat loci from the genome sequence of *Corylus avellana* 'Jefferson'. (PDF)

S2 Table Segregation at new tri-nucleotide simple sequence repeat marker loci in the hazelnut reference mapping population (OSU 252.146 x OSU 414.062). (PDF)

S3 Table Allele sizes at 132 tri-nucleotide simple sequence repeat loci developed from the 'Jefferson' hazelnut genome. (PDF)

S4 Table Amplicon sizes at 18 tri-nucleotide simple sequence repeat loci developed from the 'Jefferson' hazelnut genome. Three or four amplicons were observed in some accessions.
(PDF)
