# Supplementary material for: In silico development and characterization of tri-nucleotide simple sequence repeat markers in hazelnut (Corylus avellana L.)
Source: PLoS One. 2017 May 22;12(5):e0178061. doi: 10.1371/journal.pone.0178061 (PMC5439716; doi:10.1371/journal.pone.0178061)
Supplement: S1 Fig — The new tri-nucleotide simple sequence repeat markers are indicated by * and bold font. (PDF) [file pone.0178061.s001.pdf]

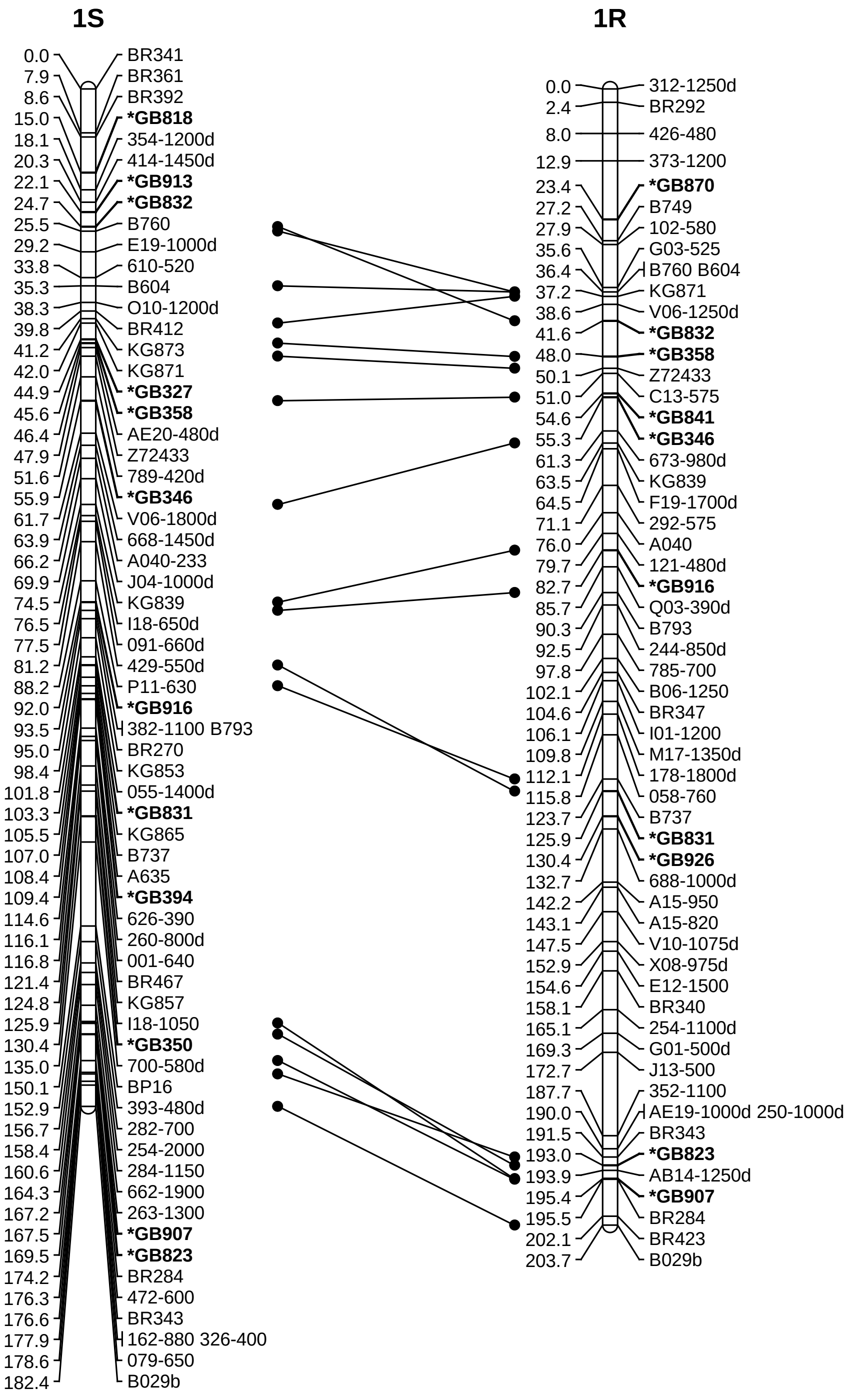

1S
0.0 BR341
7.9 BR361
8.6 BR392
15.0 *GB818
18.1 354-1200d
20.3 414-1450d
22.1 *GB913
24.7 *GB832
25.5 B760
29.2 E19-1000d
33.8 610-520
35.3 B604
38.3 O10-1200d
39.8 BR412
41.2 KG873
42.0 KG871
44.9 *GB327
45.6 *GB358
46.4 AE20-480d
47.9 Z72433
51.6 789-420d
55.9 *GB346
61.7 V06-1800d
63.9 668-1450d
66.2 A040-233
69.9 J04-1000d
74.5 KG839
76.5 I18-650d
77.5 091-660d
81.2 429-550d
88.2 P11-630
92.0 *GB916
93.5 382-1100 B793
95.0 BR270
98.4 KG853
101.8 055-1400d
103.3 *GB831
105.5 KG865
107.0 B737
108.4 A635
109.4 *GB394
114.6 626-390
116.1 260-800d
116.8 001-640
121.4 BR467
124.8 KG857
125.9 I18-1050
130.4 *GB350
135.0 700-580d
150.1 BP16
152.9 393-480d
156.7 282-700
158.4 254-2000
160.6 284-1150
164.3 662-1900
167.2 263-1300
167.5 *GB907
169.5 *GB823
174.2 BR284
176.3 472-600
176.6 BR343
177.9 162-880 326-400
178.6 079-650
182.4 B029b
1R
0.0 312-1250d
2.4 BR292
8.0 426-480
12.9 373-1200
23.4 *GB870
27.2 B749
27.9 102-580
35.6 G03-525
36.4 B760 B604
37.2 KG871
38.6 V06-1250d
41.6 *GB832
48.0 *GB358
50.1 Z72433
51.0 C13-575
54.6 *GB841
55.3 *GB346
61.3 673-980d
63.5 KG839
64.5 F19-1700d
71.1 292-575
76.0 A040
79.7 121-480d
82.7 *GB916
85.7 Q03-390d
90.3 B793
92.5 244-850d
97.8 785-700
102.1 B06-1250
104.6 BR347
106.1 I01-1200
109.8 M17-1350d
112.1 178-1800d
115.8 058-760
123.7 B737
125.9 *GB831
130.4 *GB926
132.7 688-1000d
142.2 A15-950
143.1 A15-820
147.5 V10-1075d
152.9 X08-975d
154.6 E12-1500
158.1 BR340
165.1 254-1100d
169.3 G01-500d
172.7 J13-500
187.7 352-1100
190.0 AE19-1000d 250-1000d
191.5 BR343
193.0 *GB823
193.9 AB14-1250d
195.4 *GB907
195.5 BR284
202.1 BR423
203.7 B029b

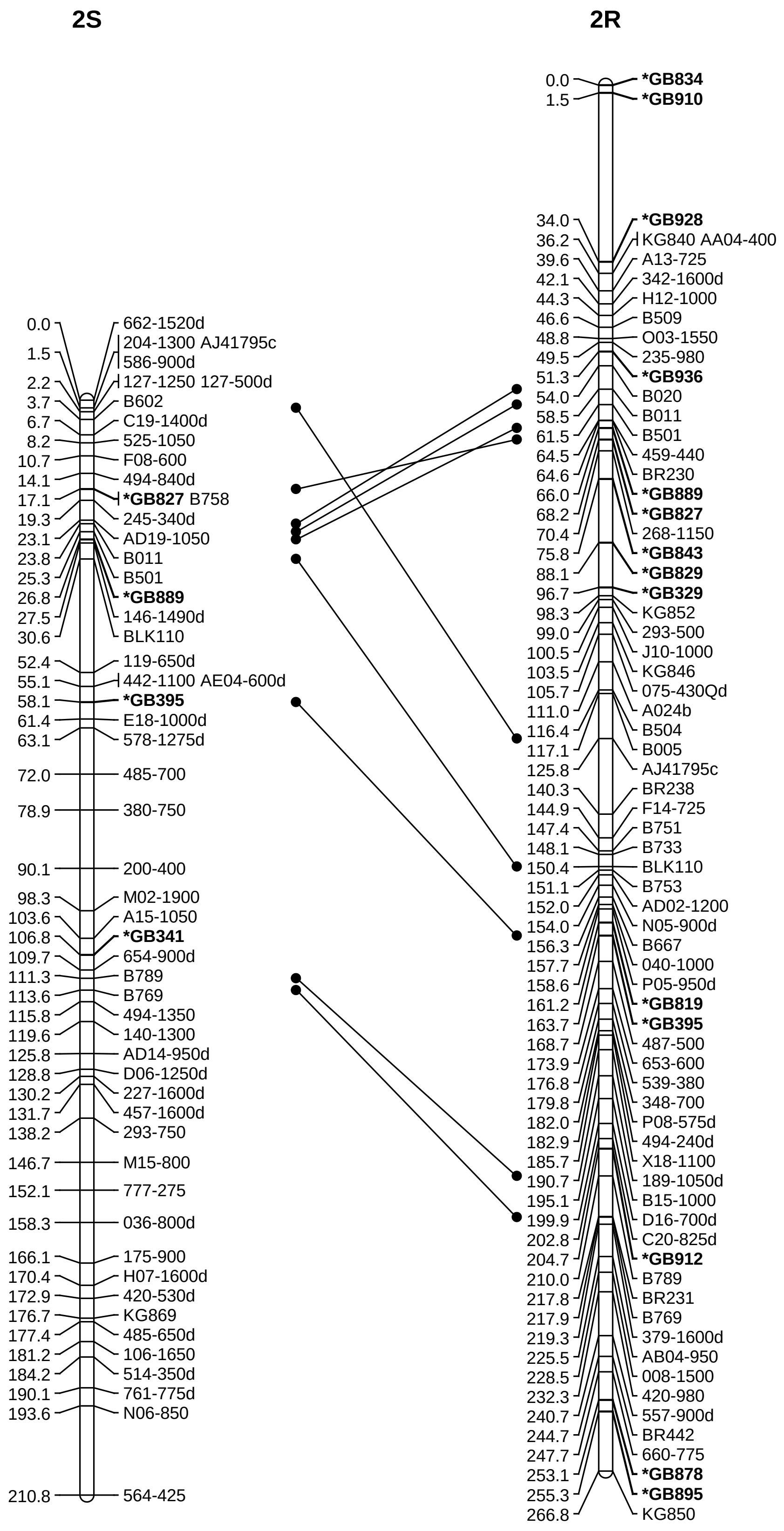
2S
0.0 662-1520d
1.5 204-1300 AJ41795c
586-900d
2.2 127-1250 127-500d
3.7 B602
6.7 C19-1400d
8.2 525-1050
10.7 F08-600
14.1 494-840d
17.1 *GB827 B758
19.3 245-340d
23.1 AD19-1050
23.8 B011
25.3 B501
26.8 *GB889
27.5 146-1490d
30.6 BLK110
52.4 119-650d
55.1 442-1100 AE04-600d
58.1 *GB395
61.4 E18-1000d
63.1 578-1275d
72.0 485-700
78.9 380-750
90.1 200-400
98.3 M02-1900
103.6 A15-1050
106.8 *GB341
109.7 654-900d
111.3 B789
113.6 B769
115.8 494-1350
119.6 140-1300
125.8 AD14-950d
128.8 D06-1250d
130.2 227-1600d
131.7 457-1600d
138.2 293-750
146.7 M15-800
152.1 777-275
158.3 036-800d
166.1 175-900
170.4 H07-1600d
172.9 420-530d
176.7 KG869
177.4 485-650d
181.2 106-1650
184.2 514-350d
190.1 761-775d
193.6 N06-850
210.8 564-425
2R
0.0 *GB834
1.5 *GB910
34.0 *GB928
36.2 KG840 AA04-400
39.6 A13-725
42.1 342-1600d
44.3 H12-1000
46.6 B509
48.8 O03-1550
49.5 235-980
51.3 *GB936
54.0 B020
58.5 B011
61.5 B501
64.5 459-440
64.6 BR230
66.0 *GB889
68.2 *GB827
70.4 268-1150
75.8 *GB843
88.1 *GB829
96.7 *GB329
98.3 KG852
99.0 293-500
100.5 J10-1000
103.5 KG846
105.7 075-430Qd
111.0 A024b
116.4 B504
117.1 B005
125.8 AJ41795c
140.3 BR238
144.9 F14-725
147.4 B751
148.1 B733
150.4 BLK110
151.1 B753
152.0 AD02-1200
154.0 N05-900d
156.3 B667
157.7 040-1000
158.6 P05-950d
161.2 *GB819
163.7 *GB395
168.7 487-500
173.9 653-600
176.8 539-380
179.8 348-700
182.0 P08-575d
182.9 494-240d
185.7 X18-1100
190.7 189-1050d
195.1 B15-1000
199.9 D16-700d
202.8 C20-825d
204.7 *GB912
210.0 B789
217.8 BR231
217.9 B769
219.3 379-1600d
225.5 AB04-950
228.5 008-1500
232.3 420-980
240.7 557-900d
244.7 BR442
247.7 660-775
253.1 *GB878
255.3 *GB895
266.8 KG850

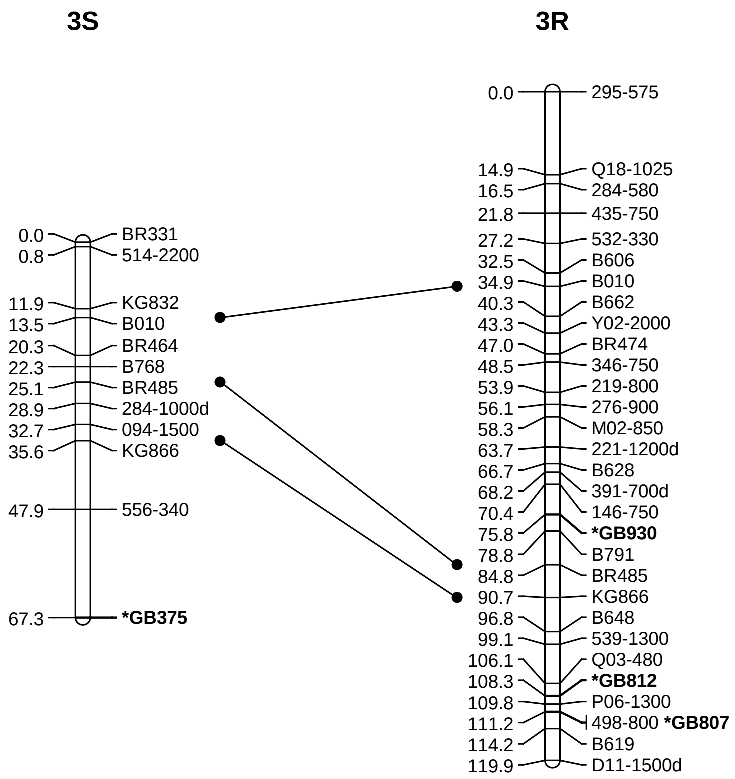
3S
3R
0.0 BR331
0.8 514-2200
11.9 KG832
13.5 B010
20.3 BR464
22.3 B768
25.1 BR485
28.9 284-1000d
32.7 094-1500
35.6 KG866
47.9 556-340
67.3 *GB375
0.0 295-575
14.9 Q18-1025
16.5 284-580
21.8 435-750
27.2 532-330
32.5 B606
34.9 B010
40.3 B662
43.3 Y02-2000
47.0 BR474
48.5 346-750
53.9 219-800
56.1 276-900
58.3 M02-850
63.7 221-1200d
66.7 B628
68.2 391-700d
70.4 146-750
75.8 *GB930
78.8 B791
84.8 BR485
90.7 KG866
96.8 B648
99.1 539-1300
106.1 Q03-480
108.3 *GB812
109.8 P06-1300
111.2 498-800 *GB807
114.2 B619
119.9 D11-1500d

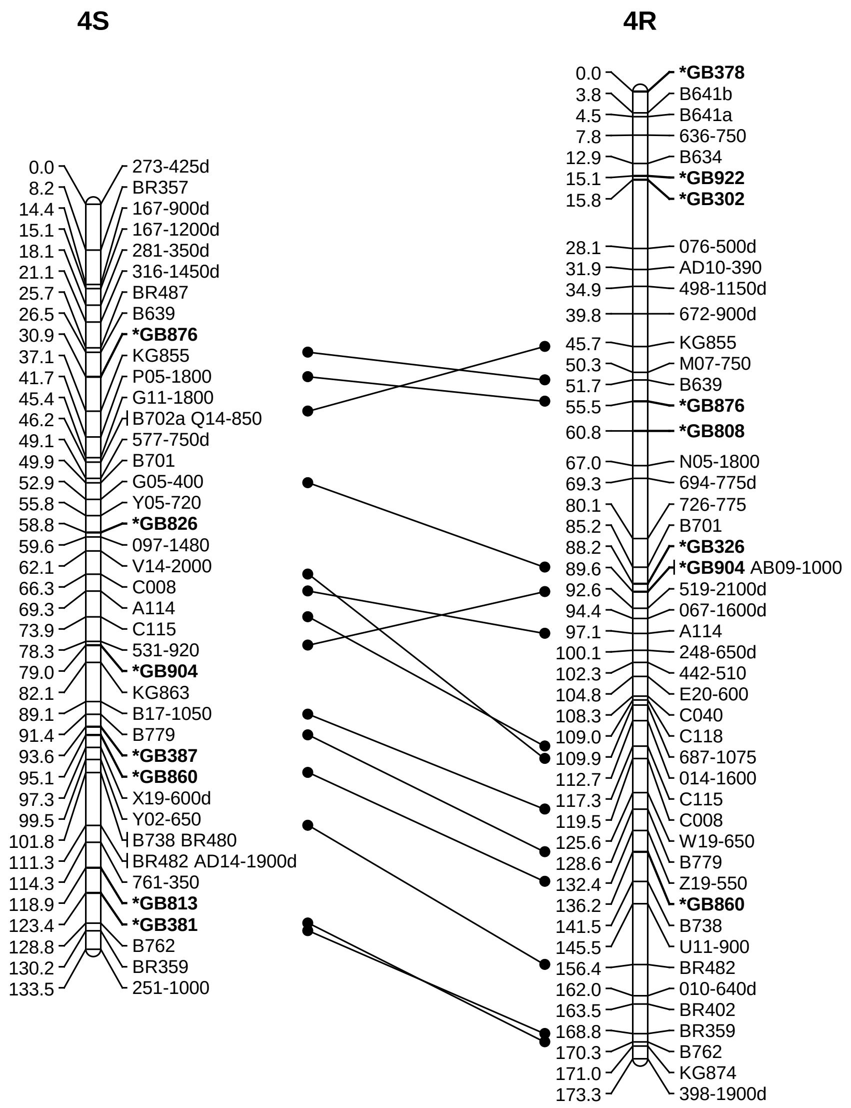
4S
4R
0.0 273-425d
8.2 BR357
14.4 167-900d
15.1 167-1200d
18.1 281-350d
21.1 316-1450d
25.7 BR487
26.5 B639
30.9 *GB876
37.1 KG855
41.7 P05-1800
45.4 G11-1800
46.2 B702a Q14-850
49.1 577-750d
49.9 B701
52.9 G05-400
55.8 Y05-720
58.8 *GB826
59.6 097-1480
62.1 V14-2000
66.3 C008
69.3 A114
73.9 C115
78.3 531-920
79.0 *GB904
82.1 KG863
89.1 B17-1050
91.4 B779
93.6 *GB387
95.1 *GB860
97.3 X19-600d
99.5 Y02-650
101.8 B738 BR480
111.3 BR482 AD14-1900d
114.3 761-350
118.9 *GB813
123.4 *GB381
128.8 B762
130.2 BR359
133.5 251-1000
0.0 *GB378
3.8 B641b
4.5 B641a
7.8 636-750
12.9 B634
15.1 *GB922
15.8 *GB302
28.1 076-500d
31.9 AD10-390
34.9 498-1150d
39.8 672-900d
45.7 KG855
50.3 M07-750
51.7 B639
55.5 *GB876
60.8 *GB808
67.0 N05-1800
69.3 694-775d
80.1 726-775
85.2 B701
88.2 *GB326
89.6 *GB904 AB09-1000
92.6 519-2100d
94.4 067-1600d
97.1 A114
100.1 248-650d
102.3 442-510
104.8 E20-600
108.3 C040
109.0 C118
109.9 687-1075
112.7 014-1600
117.3 C115
119.5 C008
125.6 W19-650
128.6 B779
132.4 Z19-550
136.2 *GB860
141.5 B738
145.5 U11-900
156.4 BR482
162.0 010-640d
163.5 BR402
168.8 BR359
170.3 B762
171.0 KG874
173.3 398-1900d

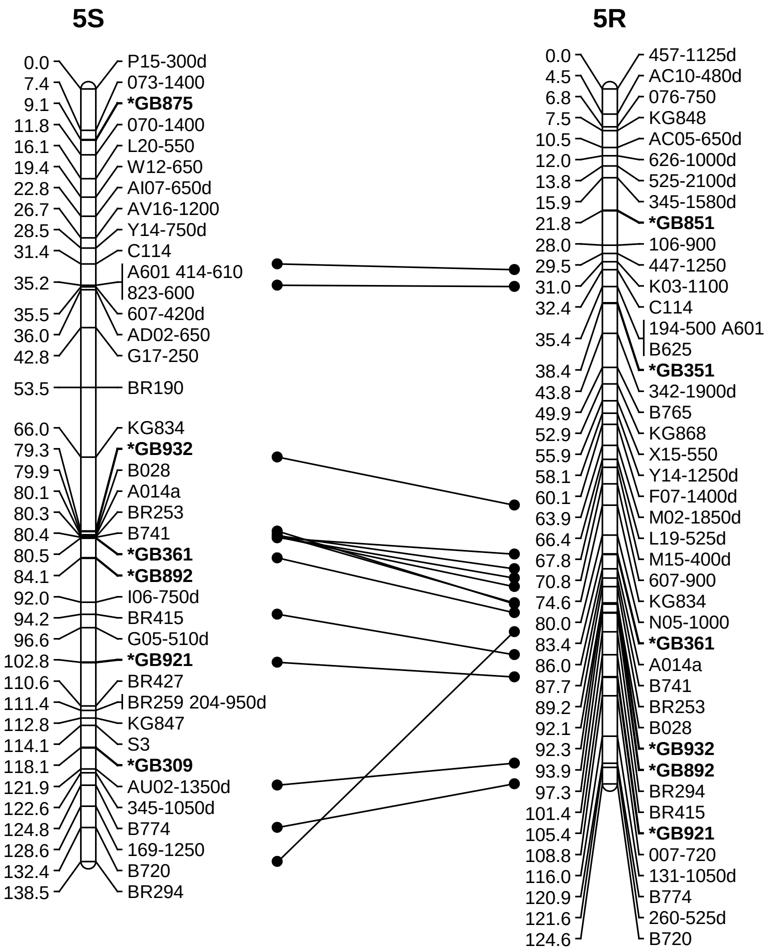
5S
5R
0.0 P15-300d
7.4 073-1400
9.1 *GB875
11.8 070-1400
16.1 L20-550
19.4 W12-650
22.8 AI07-650d
26.7 AV16-1200
28.5 Y14-750d
31.4 C114
35.2 A601 414-610 823-600
35.5 607-420d
36.0 AD02-650
42.8 G17-250
53.5 BR190
66.0 KG834
79.3 *GB932
79.9 B028
80.1 A014a
80.3 BR253
80.4 B741
80.5 *GB361
84.1 *GB892
92.0 I06-750d
94.2 BR415
96.6 G05-510d
102.8 *GB921
110.6 BR427
111.4 BR259 204-950d
112.8 KG847
114.1 S3
118.1 *GB309
121.9 AU02-1350d
122.6 345-1050d
124.8 B774
128.6 169-1250
132.4 B720
138.5 BR294
0.0 457-1125d
4.5 AC10-480d
6.8 076-750
7.5 KG848
10.5 AC05-650d
12.0 626-1000d
13.8 525-2100d
15.9 345-1580d
21.8 *GB851
28.0 106-900
29.5 447-1250
31.0 K03-1100
32.4 C114
35.4 194-500 A601 B625
38.4 *GB351
43.8 342-1900d
49.9 B765
52.9 KG868
55.9 X15-550
58.1 Y14-1250d
60.1 F07-1400d
63.9 M02-1850d
66.4 L19-525d
67.8 M15-400d
70.8 607-900
74.6 KG834
80.0 N05-1000
83.4 *GB361
86.0 A014a
87.7 B741
89.2 BR253
92.1 B028
92.3 *GB932
93.9 *GB892
97.3 BR294
101.4 BR415
105.4 *GB921
108.8 007-720
116.0 131-1050d
120.9 B774
121.6 260-525d
124.6 B720

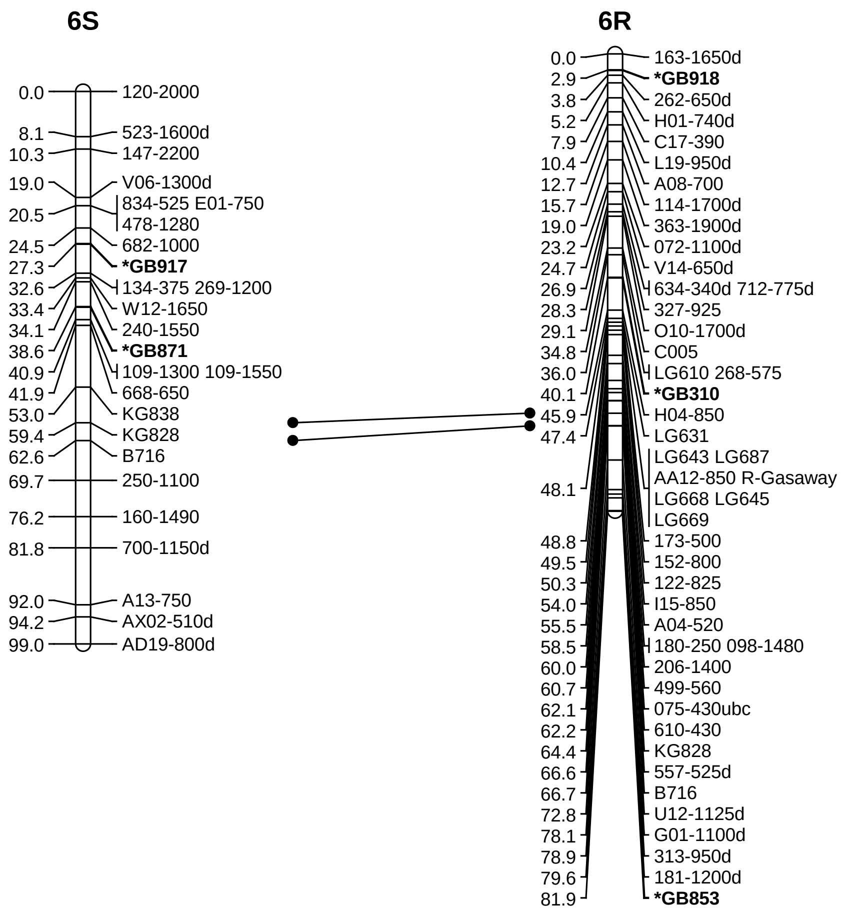

6S
0.0 120-2000
8.1 523-1600d
10.3 147-2200
19.0 V06-1300d
20.5 834-525 E01-750
478-1280
24.5 682-1000
27.3 *GB917
32.6 134-375 269-1200
33.4 W12-1650
34.1 240-1550
38.6 *GB871
40.9 109-1300 109-1550
41.9 668-650
53.0 KG838
59.4 KG828
62.6 B716
69.7 250-1100
76.2 160-1490
81.8 700-1150d
92.0 A13-750
94.2 AX02-510d
99.0 AD19-800d
6R
0.0 163-1650d
2.9 *GB918
3.8 262-650d
5.2 H01-740d
7.9 C17-390
10.4 L19-950d
12.7 A08-700
15.7 114-1700d
19.0 363-1900d
23.2 072-1100d
24.7 V14-650d
26.9 634-340d 712-775d
28.3 327-925
29.1 O10-1700d
34.8 C005
36.0 LG610 268-575
40.1 *GB310
45.9 H04-850
47.4 LG631
48.1 LG643 LG687
AA12-850 R-Gasaway
LG668 LG645
LG669
48.8 173-500
49.5 152-800
50.3 122-825
54.0 I15-850
55.5 A04-520
58.5 180-250 098-1480
60.0 206-1400
60.7 499-560
62.1 075-430ubc
62.2 610-430
64.4 KG828
66.6 557-525d
66.7 B716
72.8 U12-1125d
78.1 G01-1100d
78.9 313-950d
79.6 181-1200d
81.9 *GB853

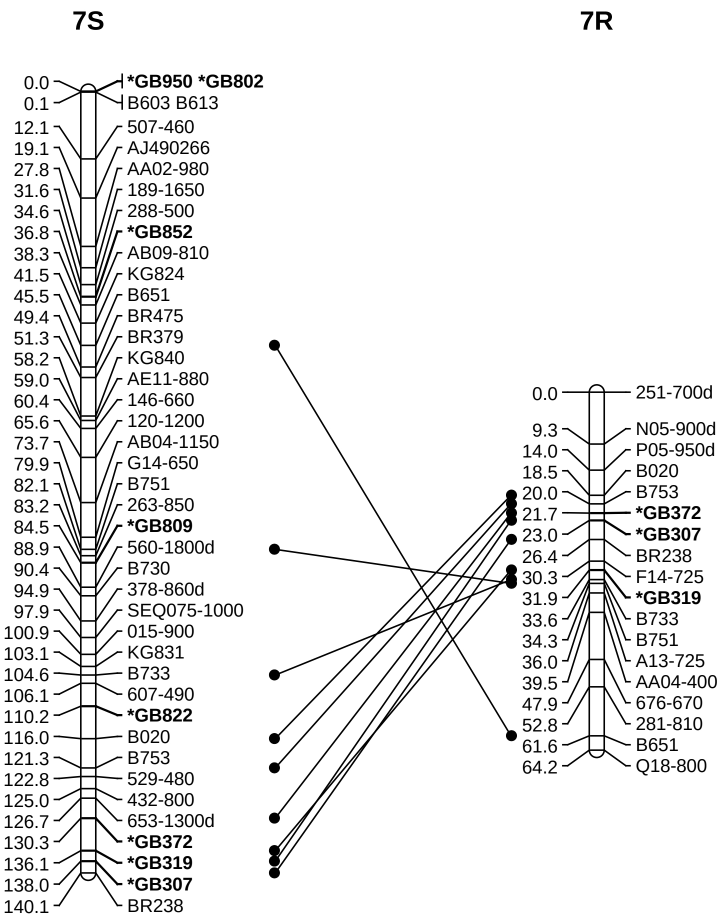
7S
7R
0.0 *GB950 *GB802
0.1 B603 B613
12.1 507-460
19.1 AJ490266
27.8 AA02-980
31.6 189-1650
34.6 288-500
36.8 *GB852
38.3 AB09-810
41.5 KG824
45.5 B651
49.4 BR475
51.3 BR379
58.2 KG840
59.0 AE11-880
60.4 146-660
65.6 120-1200
73.7 AB04-1150
79.9 G14-650
82.1 B751
83.2 263-850
84.5 *GB809
88.9 560-1800d
90.4 B730
94.9 378-860d
97.9 SEQ075-1000
100.9 015-900
103.1 KG831
104.6 B733
106.1 607-490
110.2 *GB822
116.0 B020
121.3 B753
122.8 529-480
125.0 432-800
126.7 653-1300d
130.3 *GB372
136.1 *GB319
138.0 *GB307
140.1 BR238
0.0 251-700d
9.3 N05-900d
14.0 P05-950d
18.5 B020
20.0 B753
21.7 *GB372
23.0 *GB307
26.4 BR238
30.3 F14-725
31.9 *GB319
33.6 B733
34.3 B751
36.0 A13-725
39.5 AA04-400
47.9 676-670
52.8 281-810
61.6 B651
64.2 Q18-800

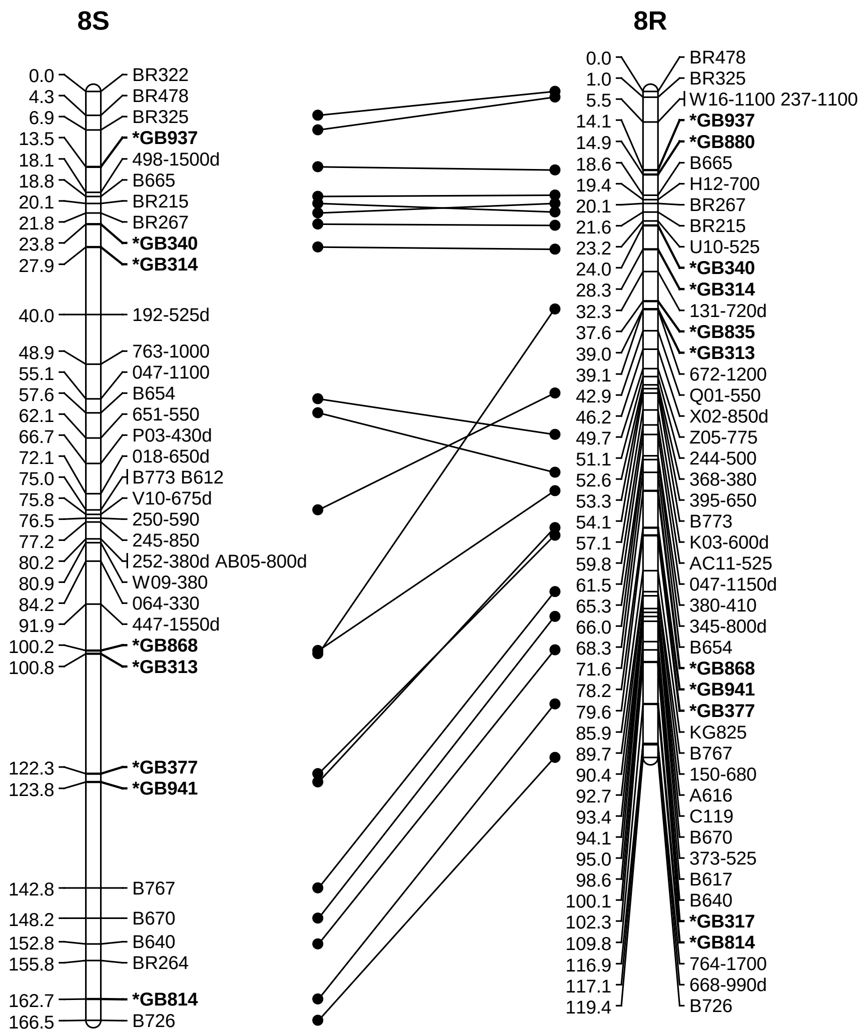
8S
8R
0.0 BR322
4.3 BR478
6.9 BR325
13.5 *GB937
18.1 498-1500d
18.8 B665
20.1 BR215
21.8 BR267
23.8 *GB340
27.9 *GB314
40.0 192-525d
48.9 763-1000
55.1 047-1100
57.6 B654
62.1 651-550
66.7 P03-430d
72.1 018-650d
75.0 B773 B612
75.8 V10-675d
76.5 250-590
77.2 245-850
80.2 252-380d AB05-800d
80.9 W09-380
84.2 064-330
91.9 447-1550d
100.2 *GB868
100.8 *GB313
122.3 *GB377
123.8 *GB941
142.8 B767
148.2 B670
152.8 B640
155.8 BR264
162.7 *GB814
166.5 B726
0.0 BR478
1.0 BR325
5.5 W16-1100 237-1100
14.1 *GB937
14.9 *GB880
18.6 B665
19.4 H12-700
20.1 BR267
21.6 BR215
23.2 U10-525
24.0 *GB340
28.3 *GB314
32.3 131-720d
37.6 *GB835
39.0 *GB313
39.1 672-1200
42.9 Q01-550
46.2 X02-850d
49.7 Z05-775
51.1 244-500
52.6 368-380
53.3 395-650
54.1 B773
57.1 K03-600d
59.8 AC11-525
61.5 047-1150d
65.3 380-410
66.0 345-800d
68.3 B654
71.6 *GB868
78.2 *GB941
79.6 *GB377
85.9 KG825
89.7 B767
90.4 150-680
92.7 A616
93.4 C119
94.1 B670
95.0 373-525
98.6 B617
100.1 B640
102.3 *GB317
109.8 *GB814
116.9 764-1700
117.1 668-990d
119.4 B726

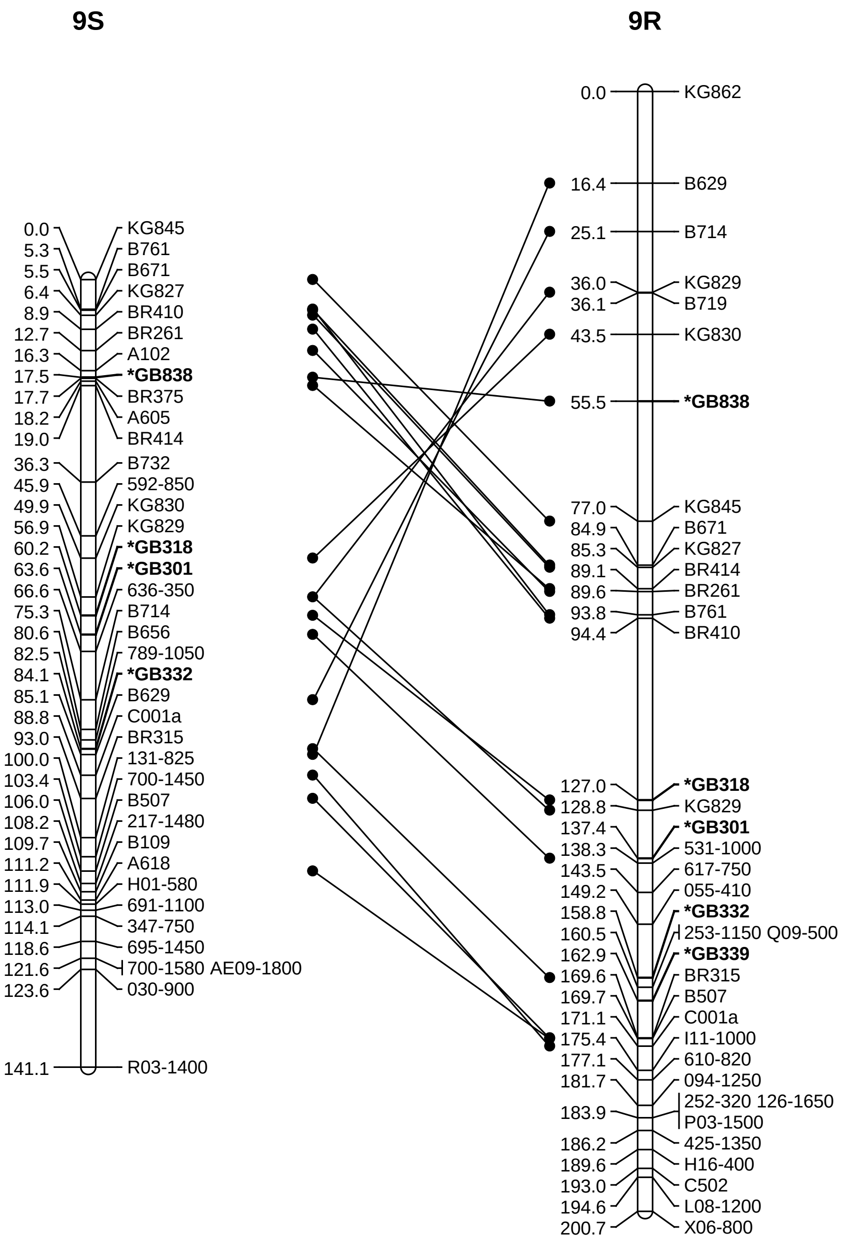

9S
9R
0.0 KG845
5.3 B761
5.5 B671
6.4 KG827
8.9 BR410
12.7 BR261
16.3 A102
17.5 *GB838
17.7 BR375
18.2 A605
19.0 BR414
36.3 B732
45.9 592-850
49.9 KG830
56.9 KG829
60.2 *GB318
63.6 *GB301
66.6 636-350
75.3 B714
80.6 B656
82.5 789-1050
84.1 *GB332
85.1 B629
88.8 C001a
93.0 BR315
100.0 131-825
103.4 700-1450
106.0 B507
108.2 217-1480
109.7 B109
111.2 A618
111.9 H01-580
113.0 691-1100
114.1 347-750
118.6 695-1450
121.6 700-1580 AE09-1800
123.6 030-900
141.1 R03-1400
0.0 KG862
16.4 B629
25.1 B714
36.0 KG829
36.1 B719
43.5 KG830
55.5 *GB838
77.0 KG845
84.9 B671
85.3 KG827
89.1 BR414
89.6 BR261
93.8 B761
94.4 BR410
127.0 *GB318
128.8 KG829
137.4 *GB301
138.3 531-1000
143.5 617-750
149.2 055-410
158.8 *GB332
160.5 253-1150 Q09-500
162.9 *GB339
169.6 BR315
169.7 B507
171.1 C001a
175.4 I11-1000
177.1 610-820
181.7 094-1250
183.9 252-320 126-1650 P03-1500
186.2 425-1350
189.6 H16-400
193.0 C502
194.6 L08-1200
200.7 X06-800

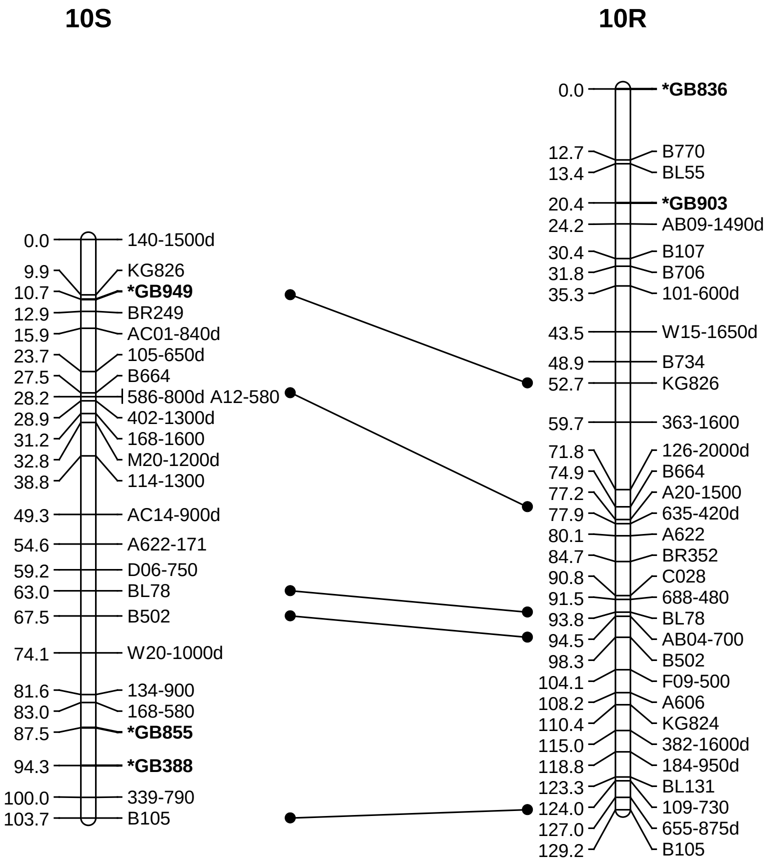
10S
10R
0.0 140-1500d
9.9 KG826
10.7 *GB949
12.9 BR249
15.9 AC01-840d
23.7 105-650d
27.5 B664
28.2 586-800d A12-580
28.9 402-1300d
31.2 168-1600
32.8 M20-1200d
38.8 114-1300
49.3 AC14-900d
54.6 A622-171
59.2 D06-750
63.0 BL78
67.5 B502
74.1 W20-1000d
81.6 134-900
83.0 168-580
87.5 *GB855
94.3 *GB388
100.0 339-790
103.7 B105
0.0 *GB836
12.7 B770
13.4 BL55
20.4 *GB903
24.2 AB09-1490d
30.4 B107
31.8 B706
35.3 101-600d
43.5 W15-1650d
48.9 B734
52.7 KG826
59.7 363-1600
71.8 126-2000d
74.9 B664
77.2 A20-1500
77.9 635-420d
80.1 A622
84.7 BR352
90.8 C028
91.5 688-480
93.8 BL78
94.5 AB04-700
98.3 B502
104.1 F09-500
108.2 A606
110.4 KG824
115.0 382-1600d
118.8 184-950d
123.3 BL131
124.0 109-730
127.0 655-875d
129.2 B105

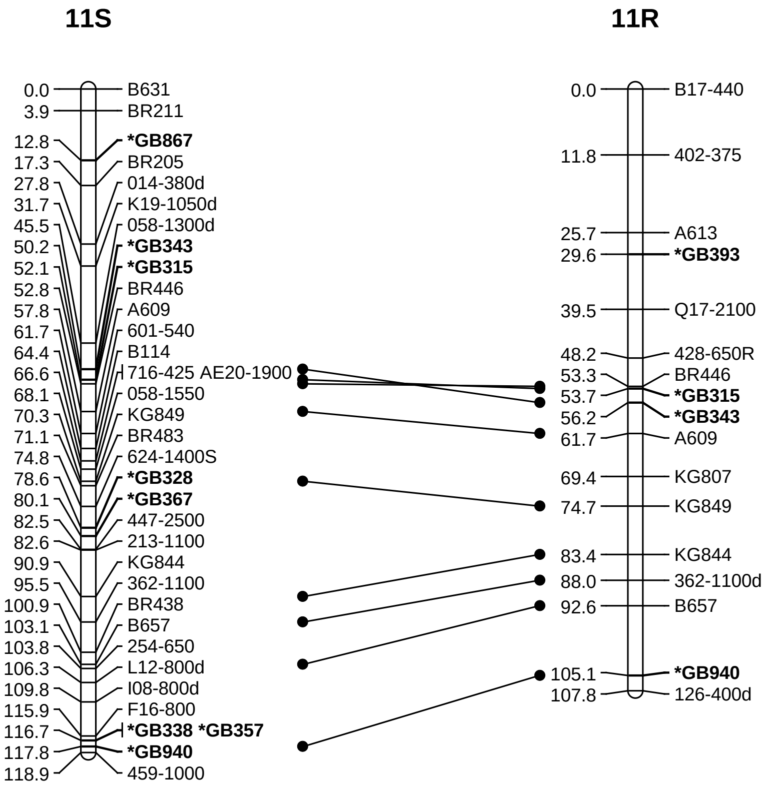
11S
11R
0.0 B631
3.9 BR211
12.8 *GB867
17.3 BR205
27.8 014-380d
31.7 K19-1050d
45.5 058-1300d
50.2 *GB343
52.1 *GB315
52.8 BR446
57.8 A609
61.7 601-540
64.4 B114
66.6 716-425 AE20-1900
68.1 058-1550
70.3 KG849
71.1 BR483
74.8 624-1400S
78.6 *GB328
80.1 *GB367
82.5 447-2500
82.6 213-1100
90.9 KG844
95.5 362-1100
100.9 BR438
103.1 B657
103.8 254-650
106.3 L12-800d
109.8 I08-800d
115.9 F16-800
116.7 *GB338 *GB357
117.8 *GB940
118.9 459-1000
0.0 B17-440
11.8 402-375
25.7 A613
29.6 *GB393
39.5 Q17-2100
48.2 428-650R
53.3 BR446
53.7 *GB315
56.2 *GB343
61.7 A609
69.4 KG807
74.7 KG849
83.4 KG844
88.0 362-1100d
92.6 B657
105.1 *GB940
107.8 126-400d
