# Supplementary figures and images for: In silico development and characterization of tri-nucleotide simple sequence repeat markers in hazelnut (Corylus avellana L.)
Source: PLoS One. 2017 May 22;12(5):e0178061. doi: 10.1371/journal.pone.0178061 (PMC5439716; doi:10.1371/journal.pone.0178061)

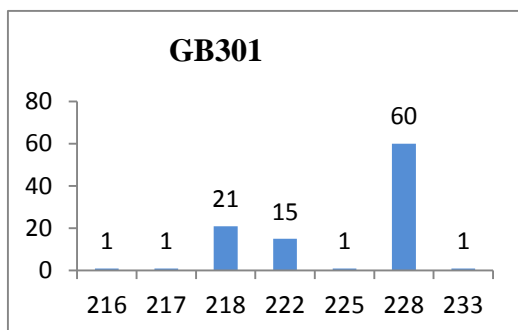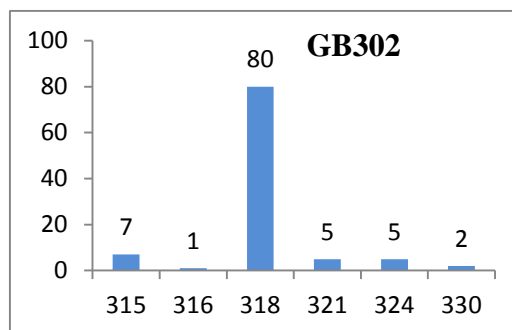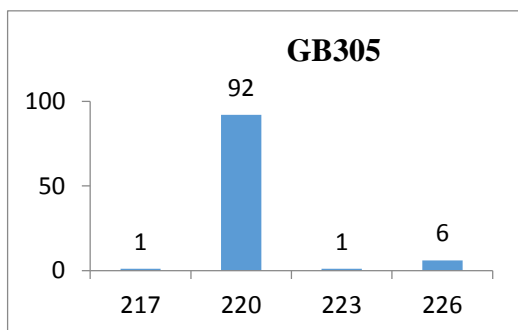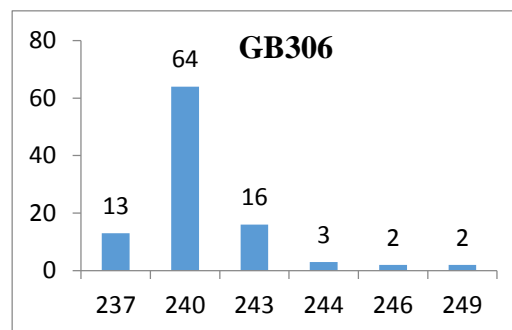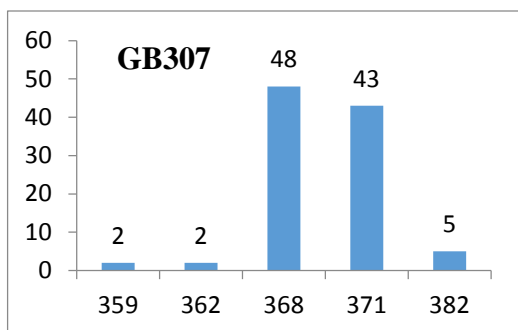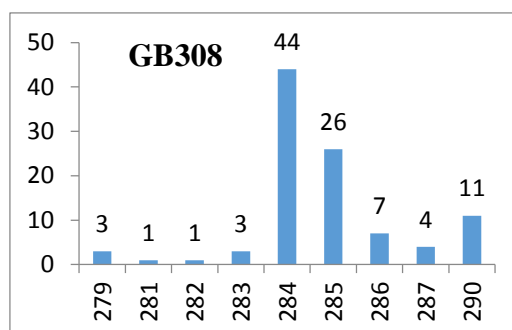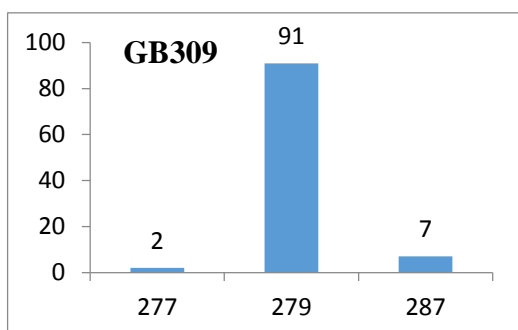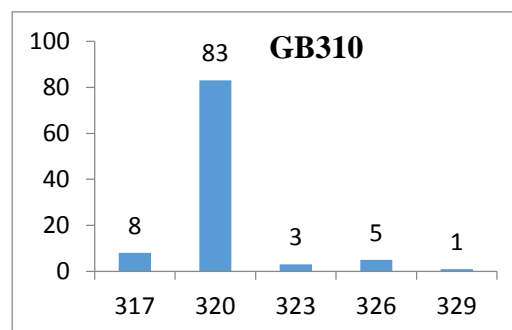

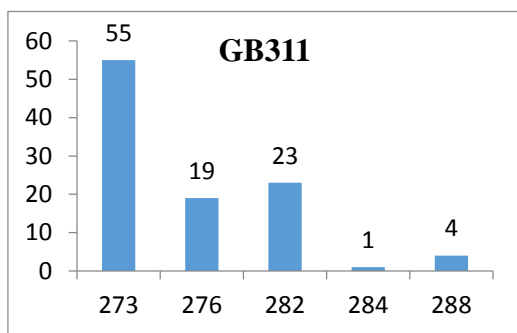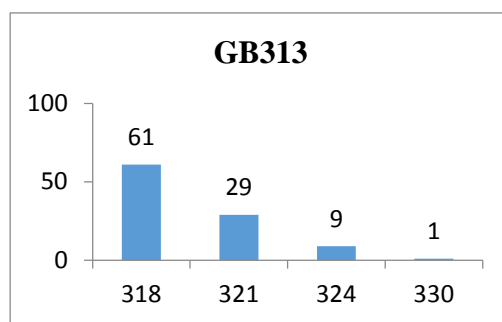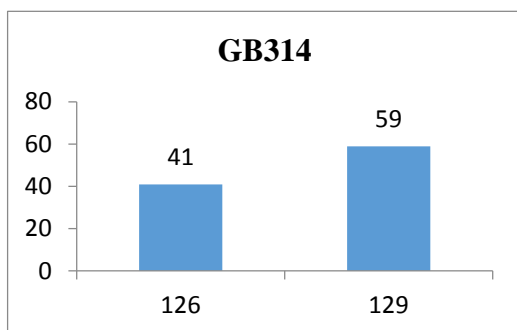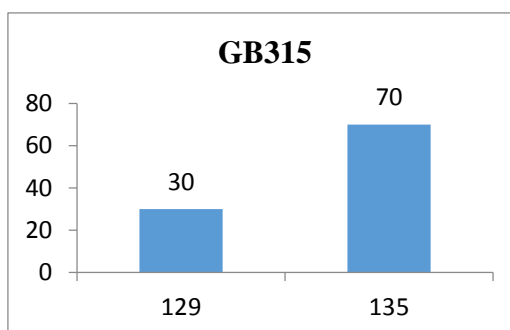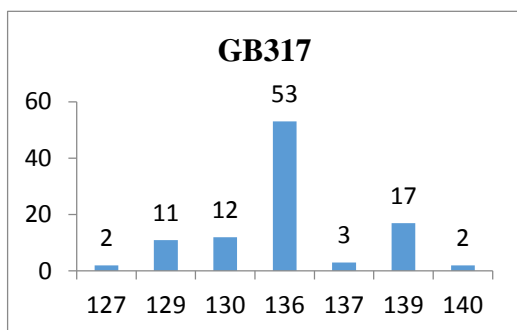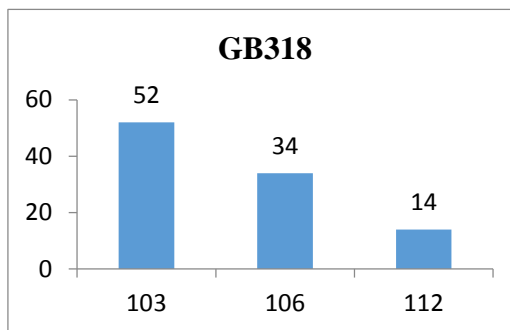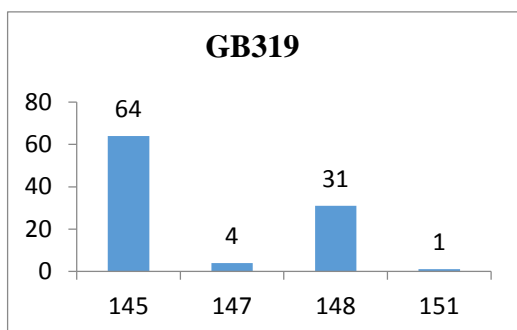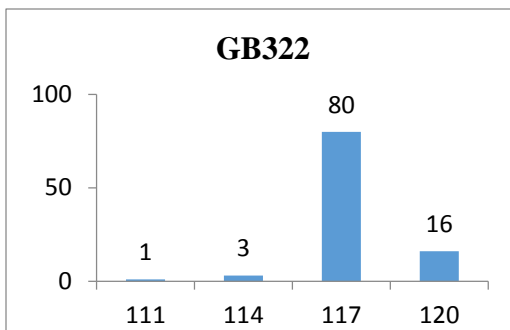

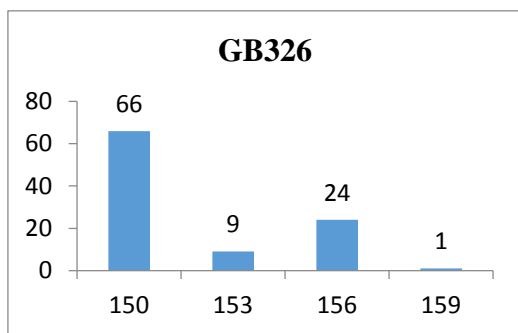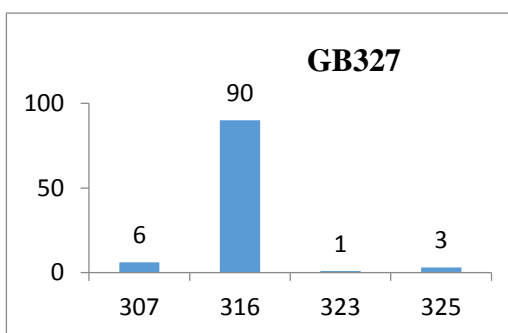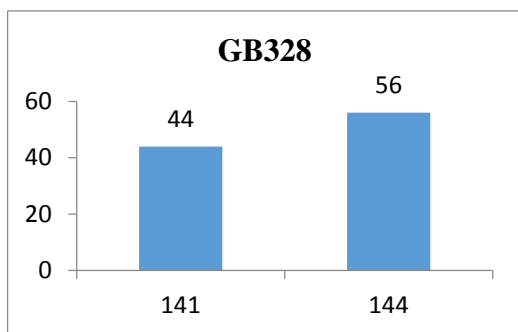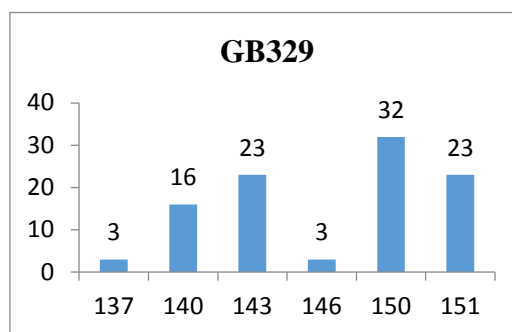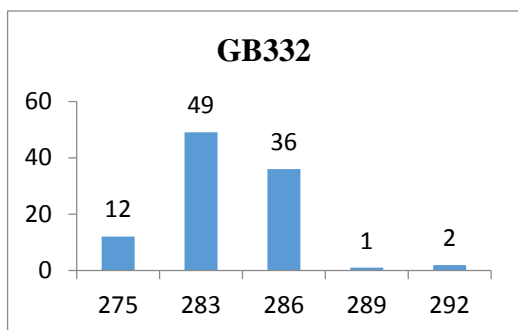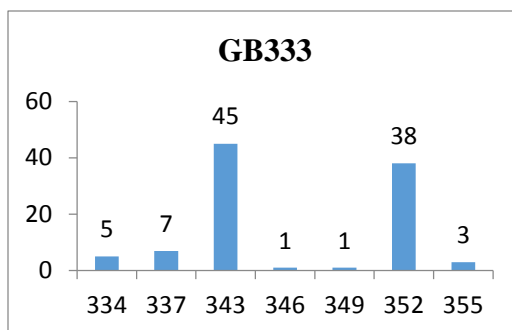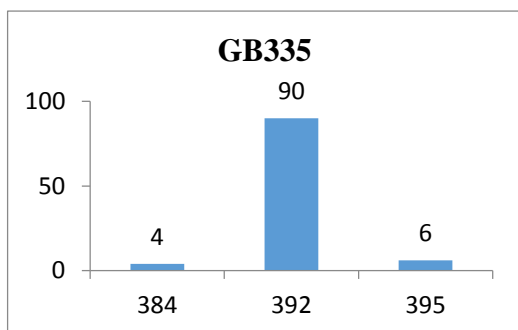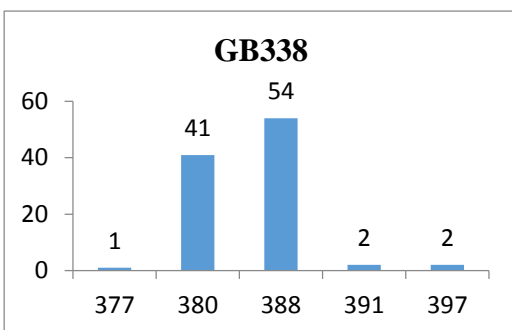

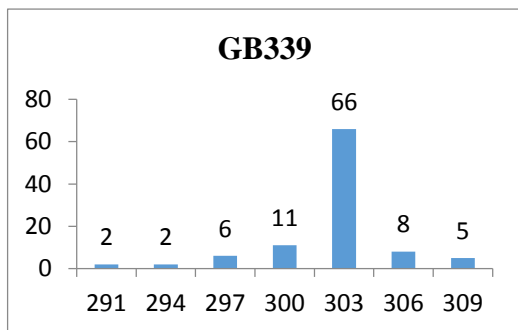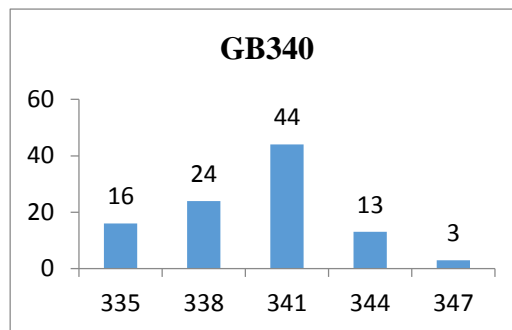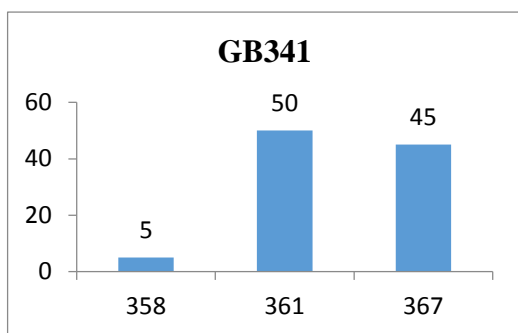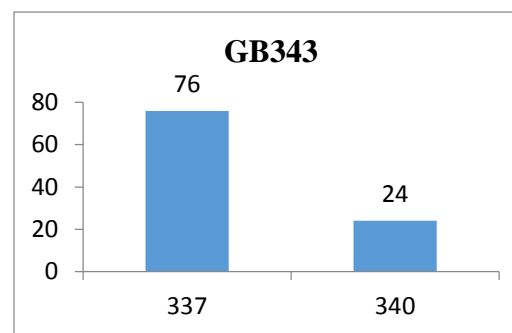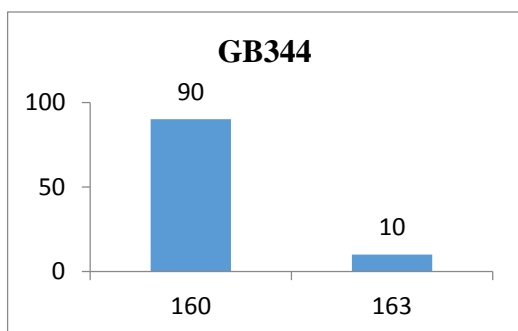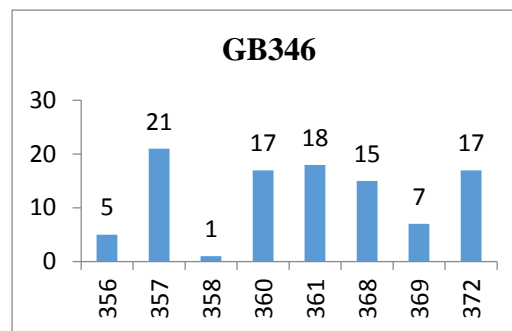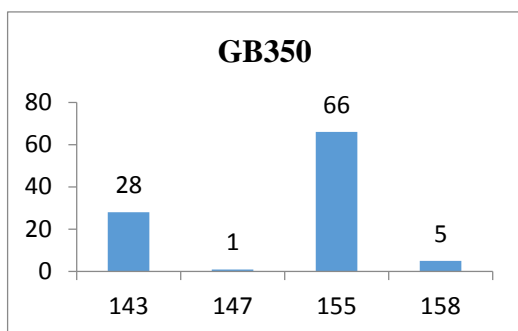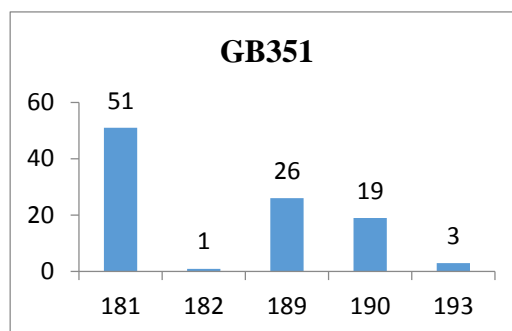

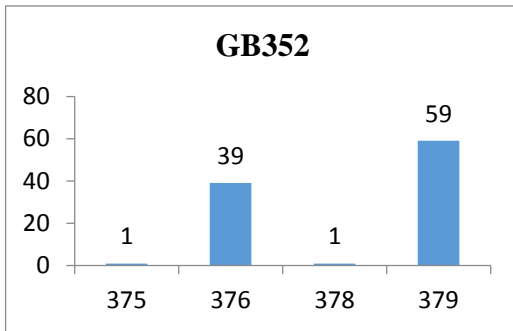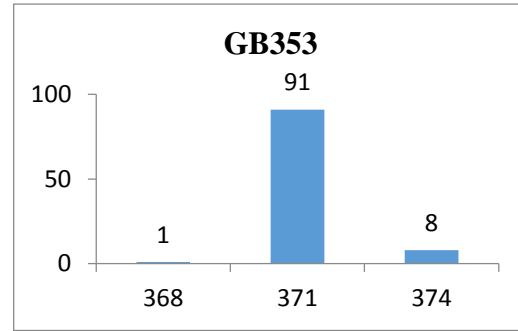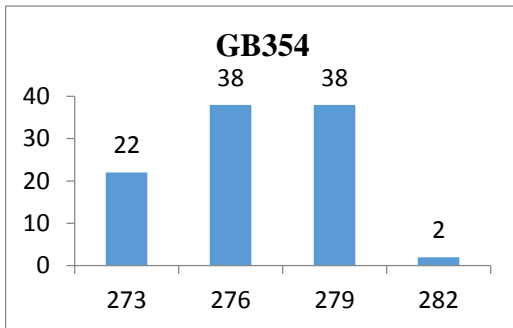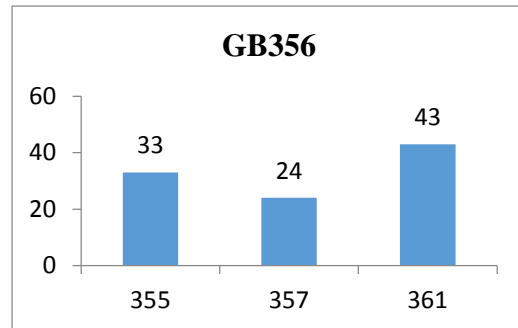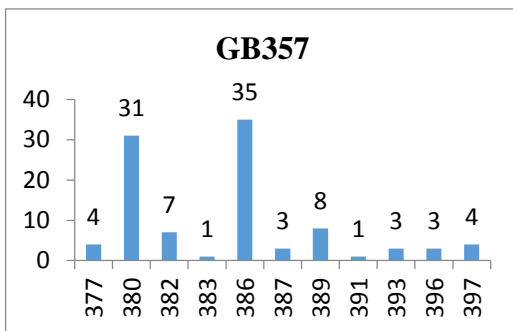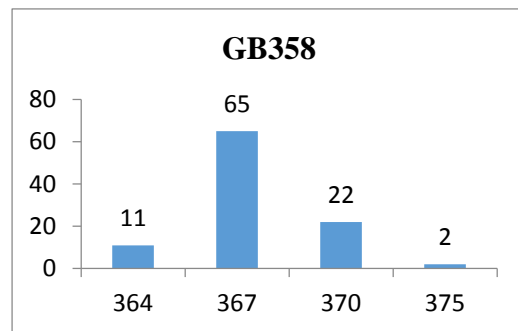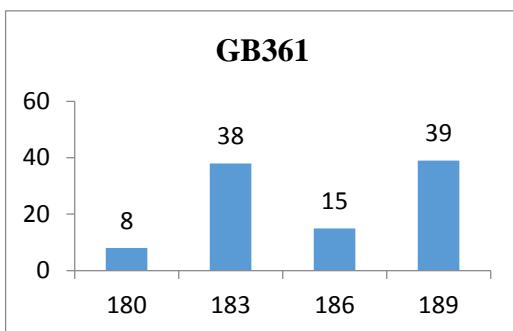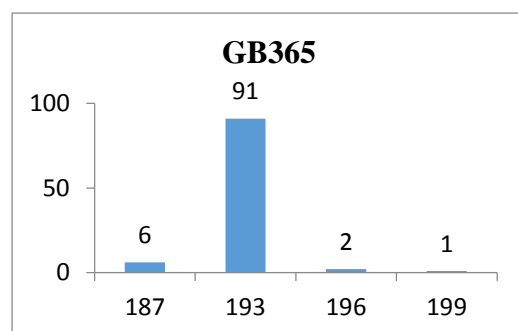

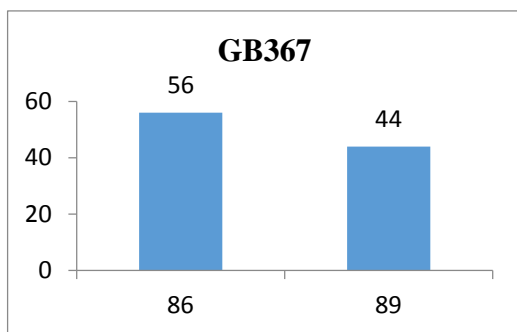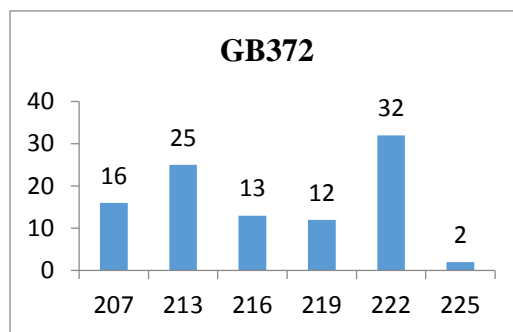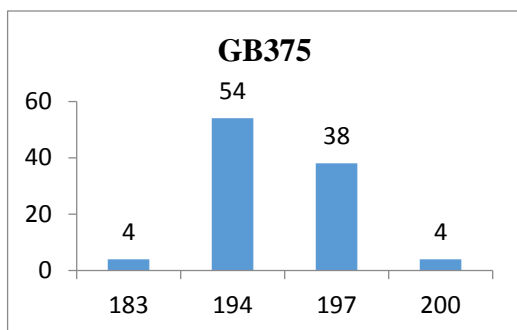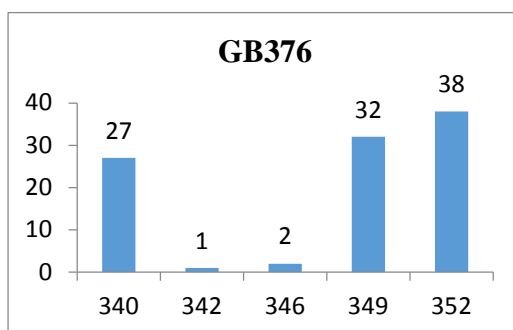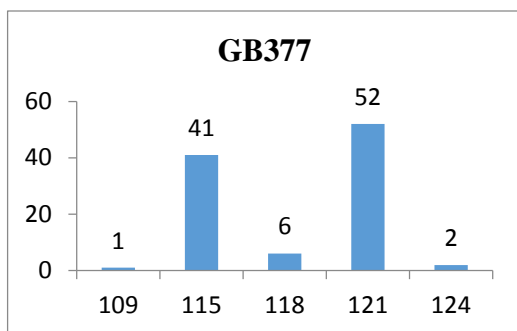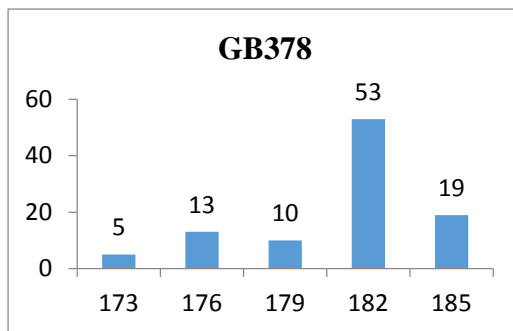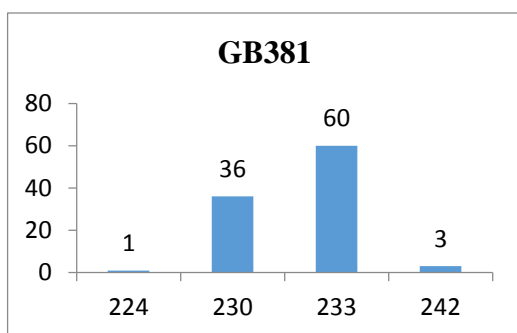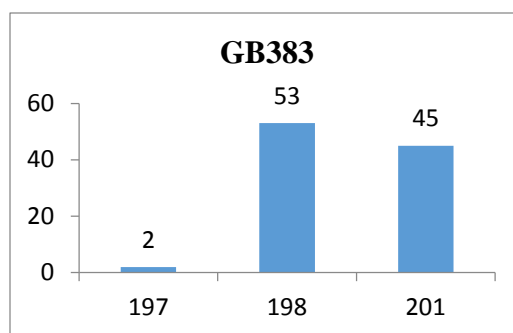

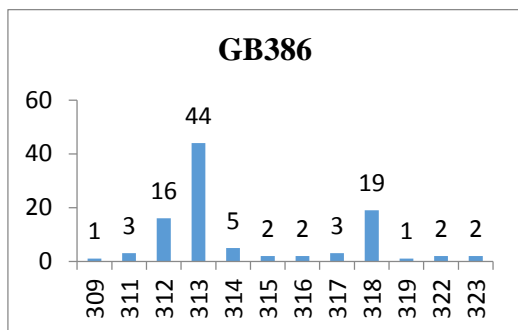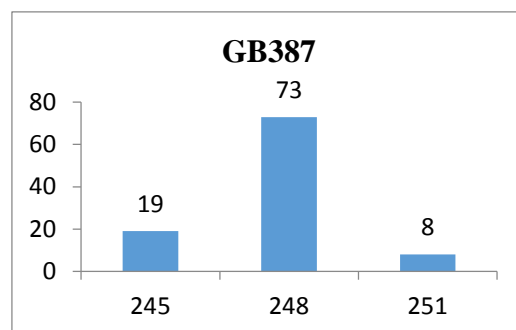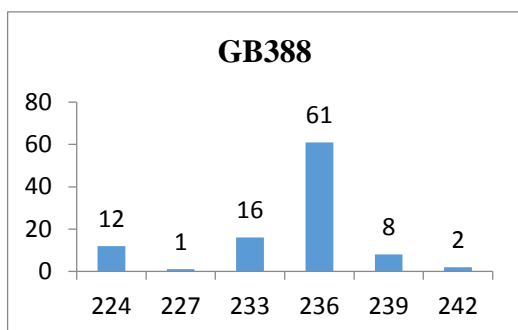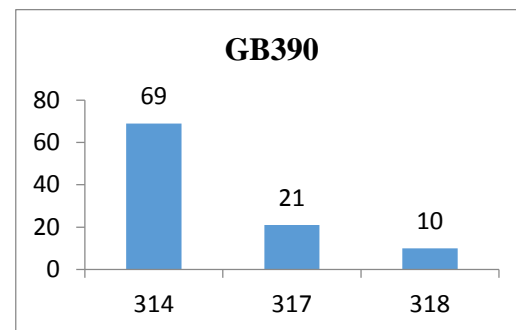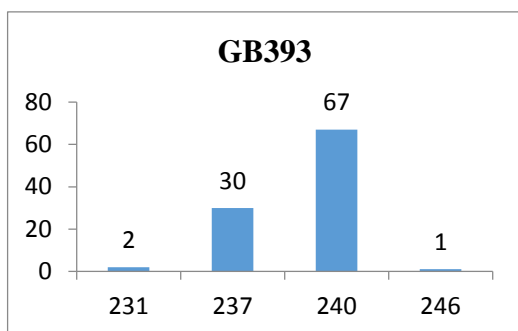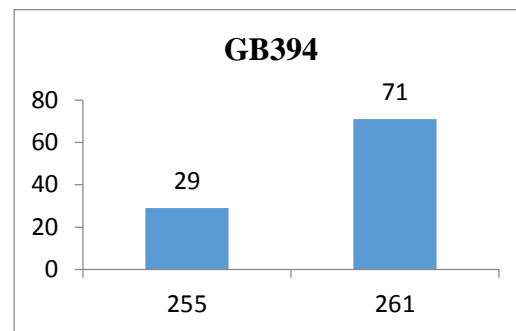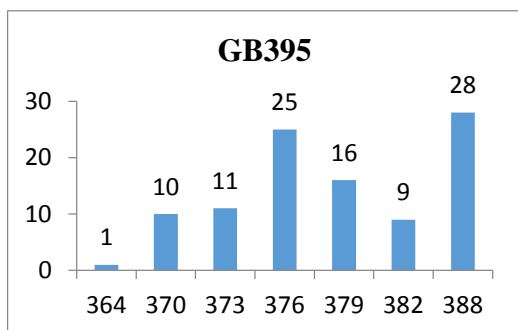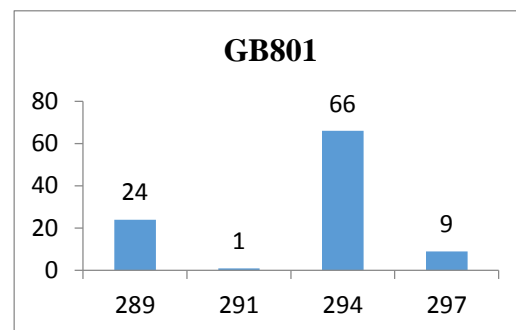

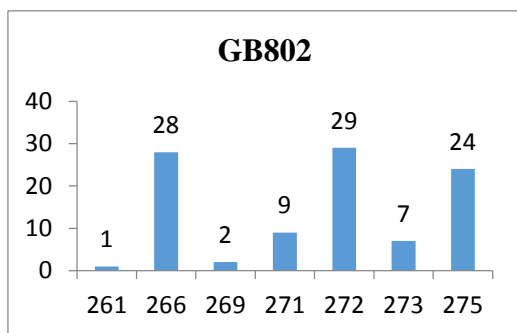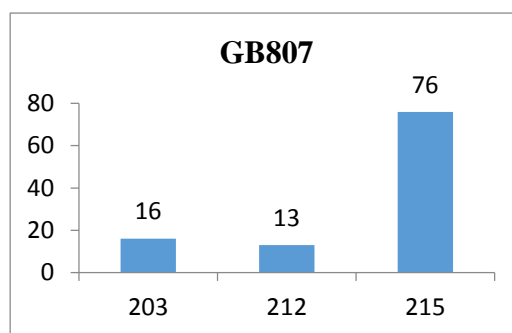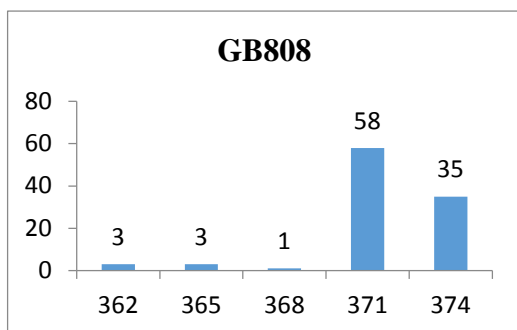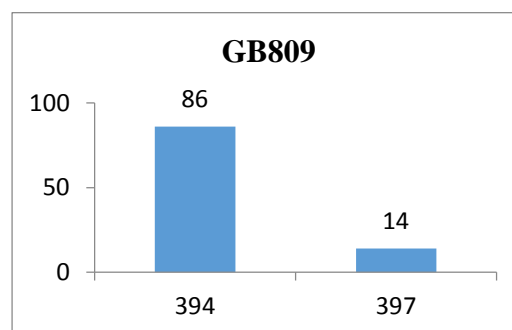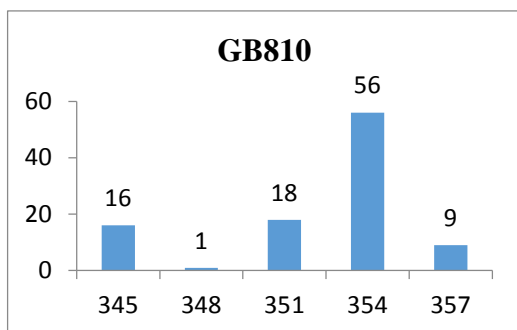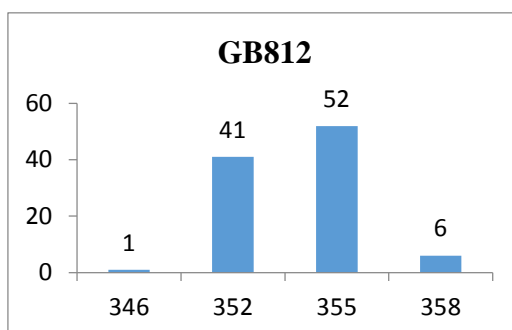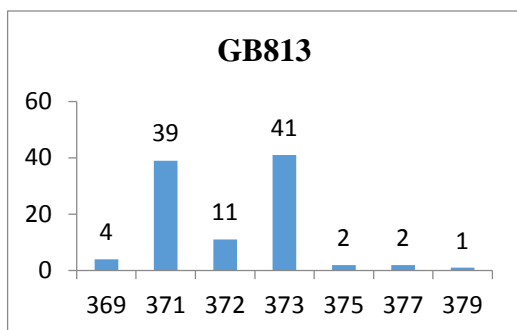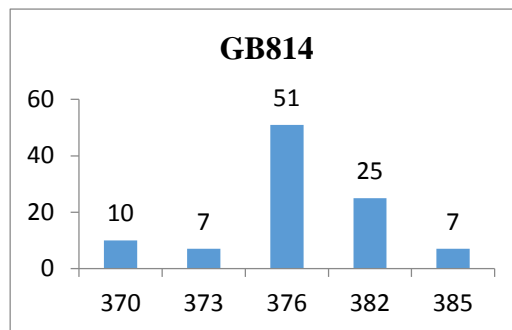

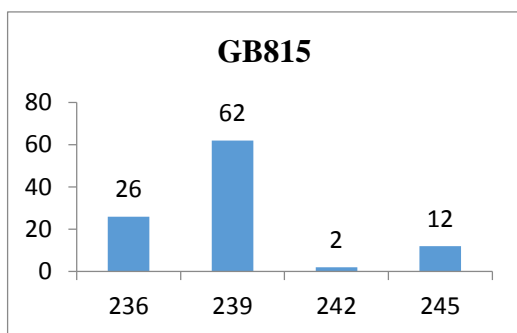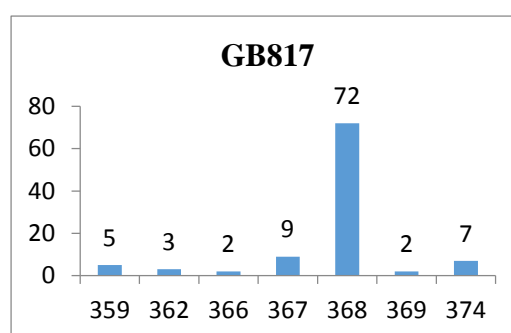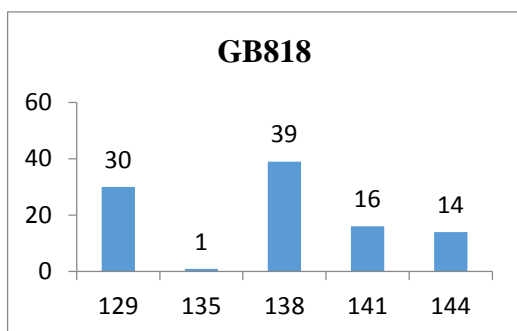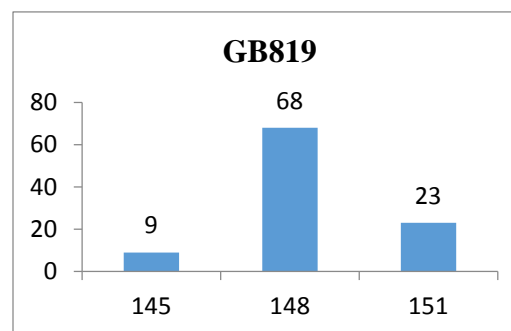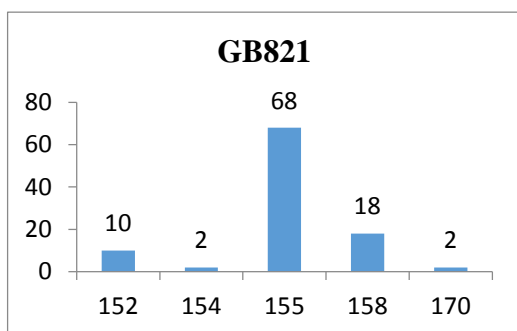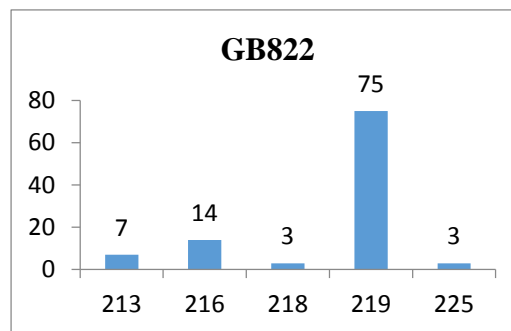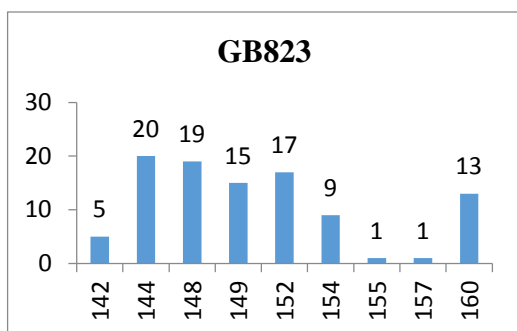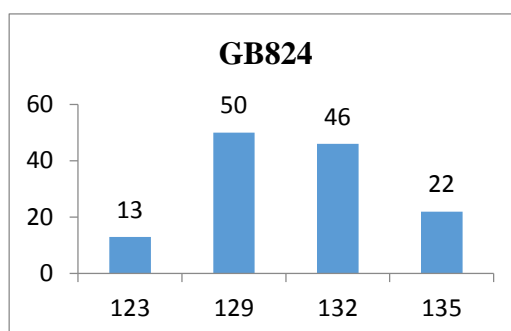

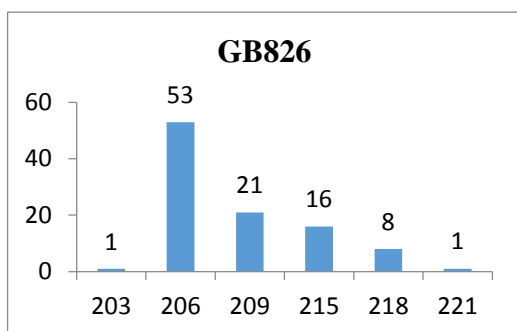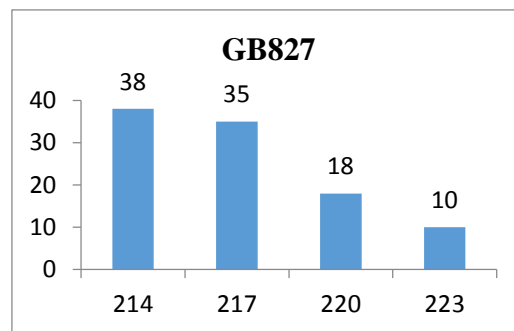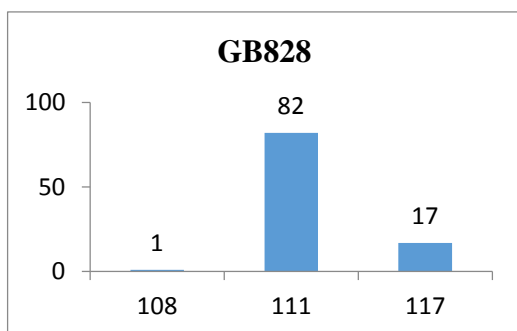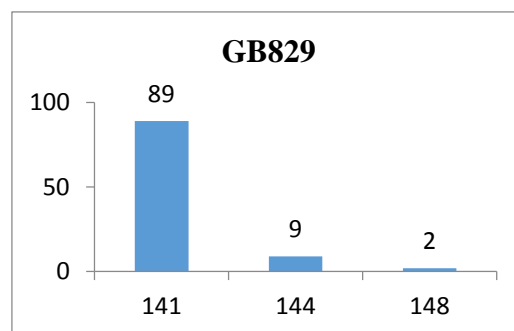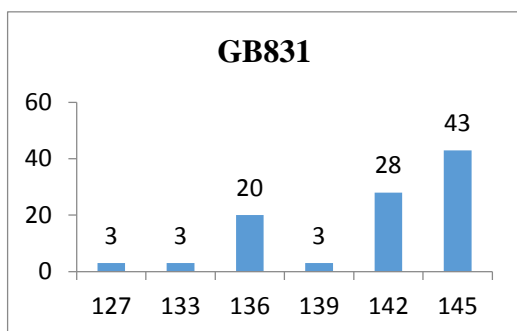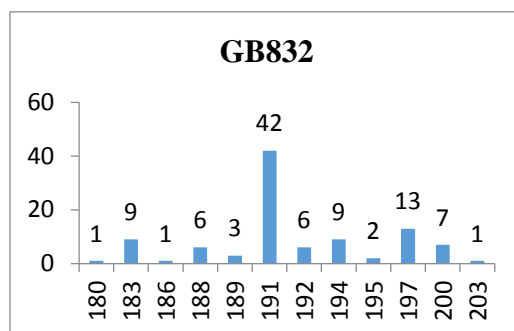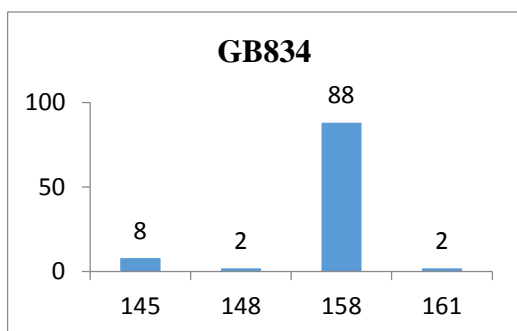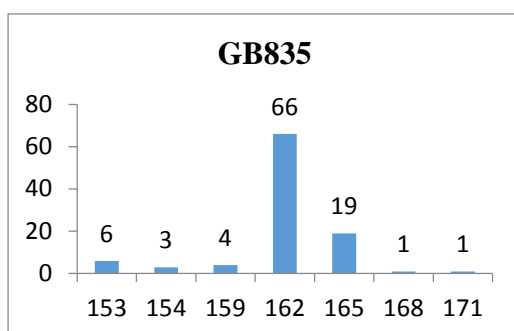

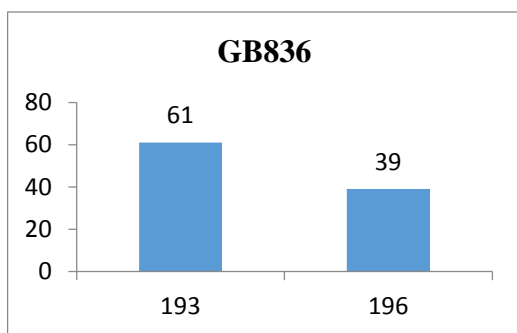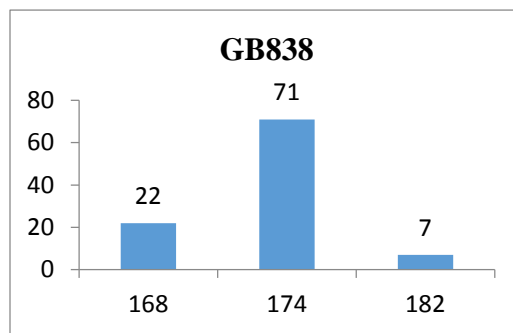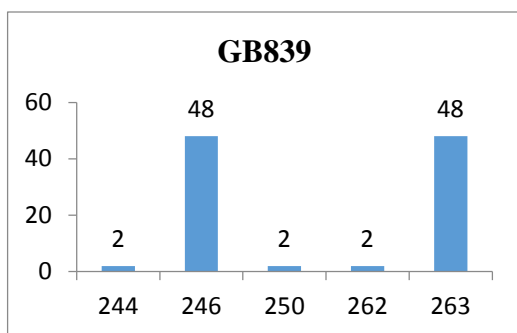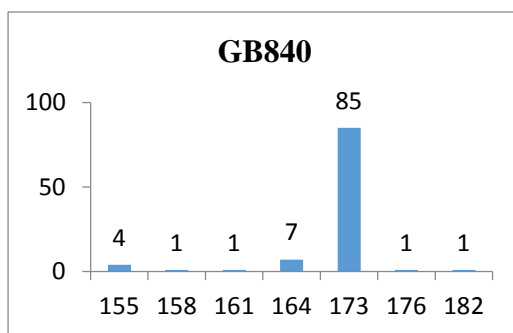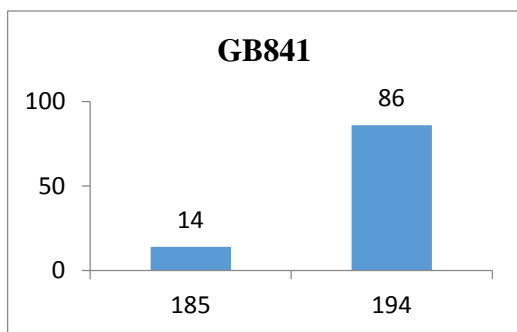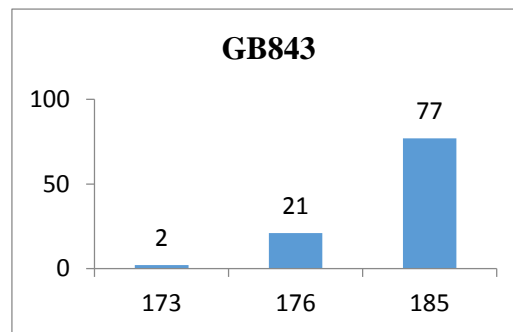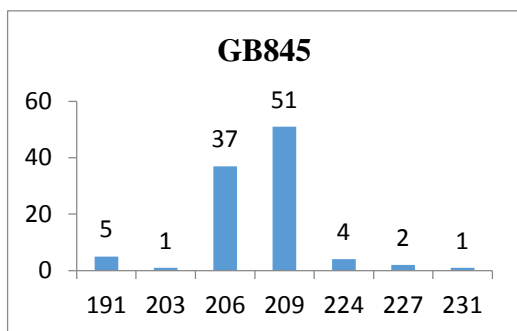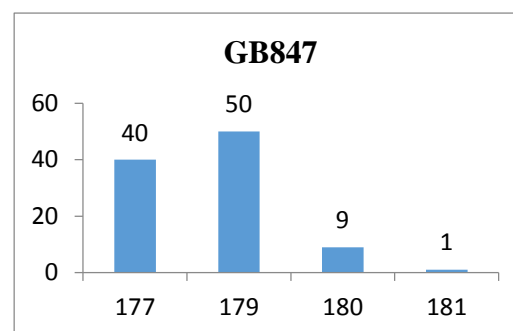

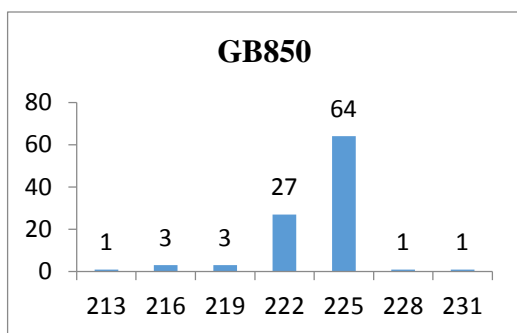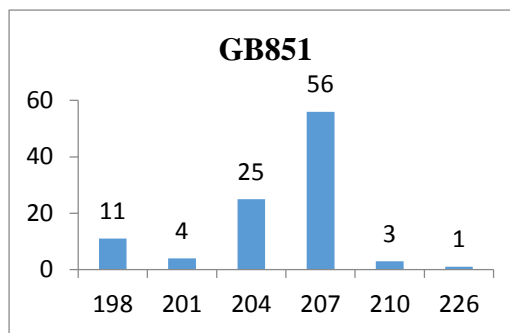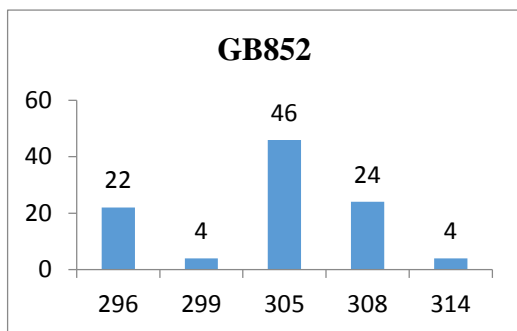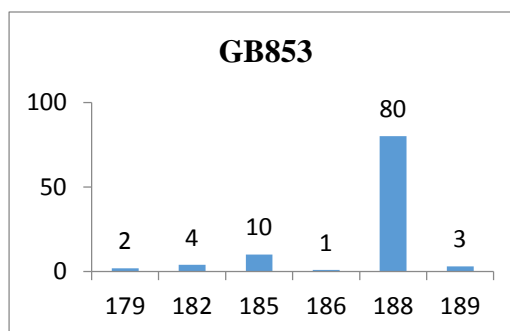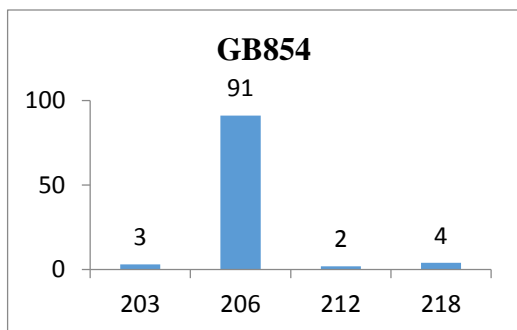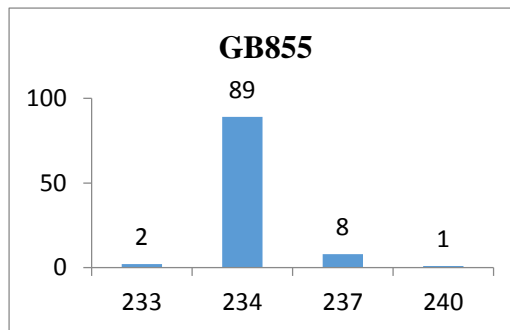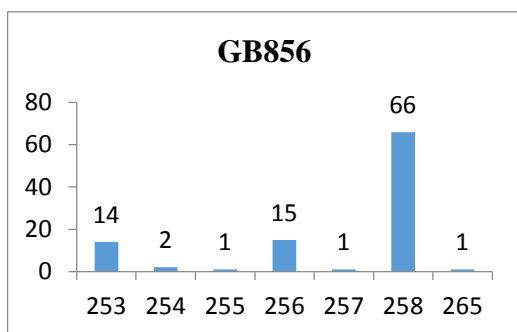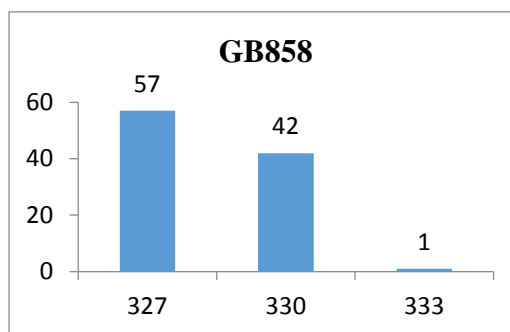

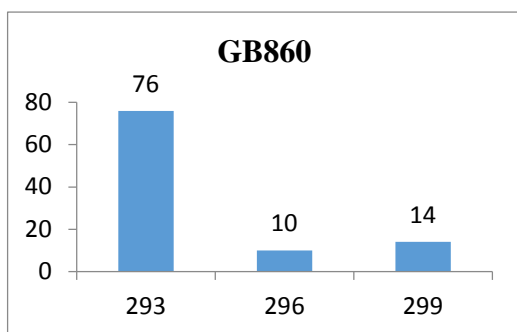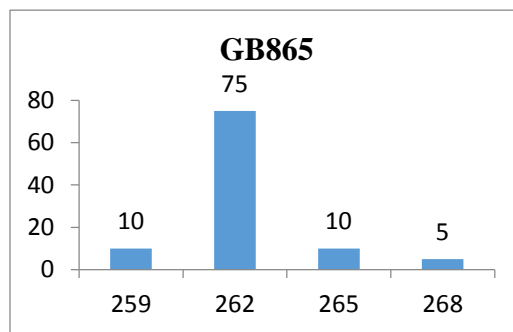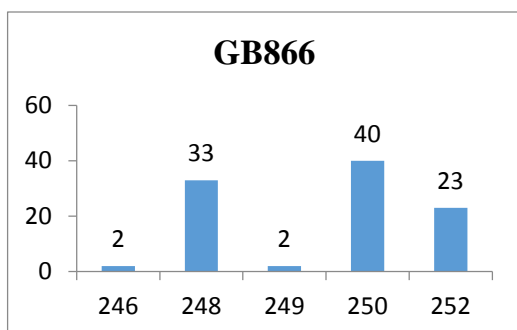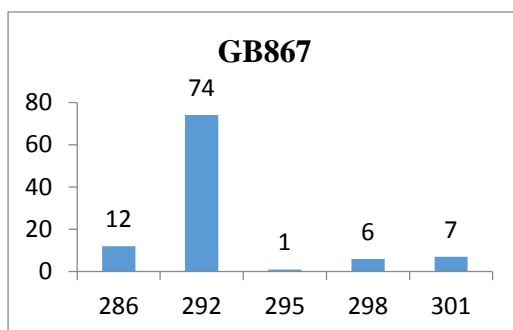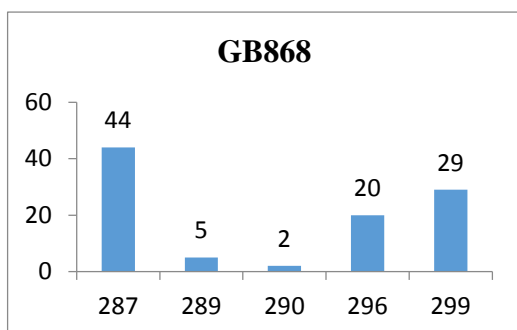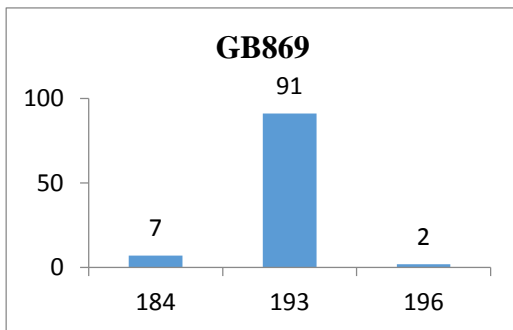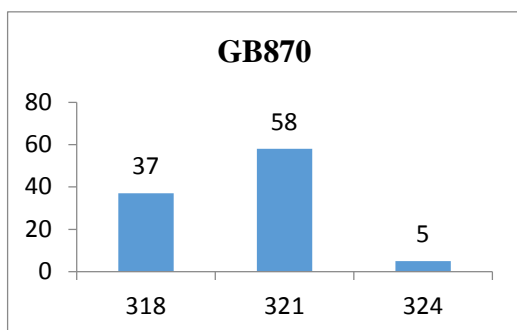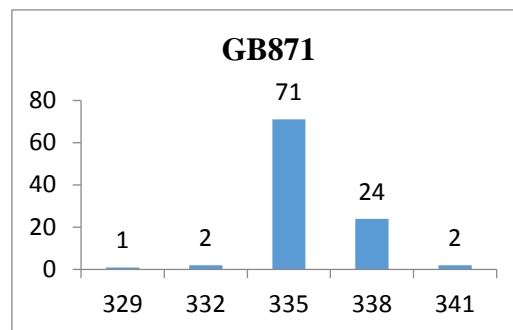

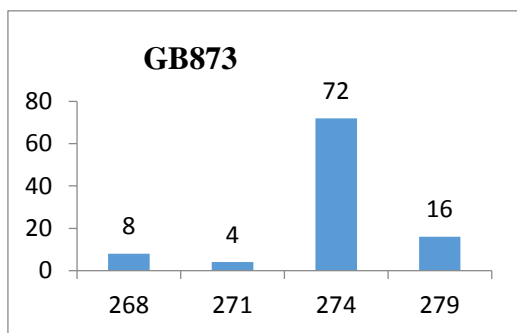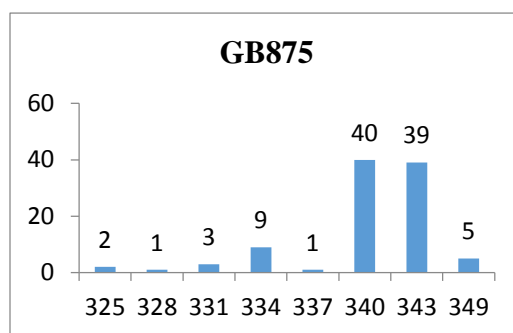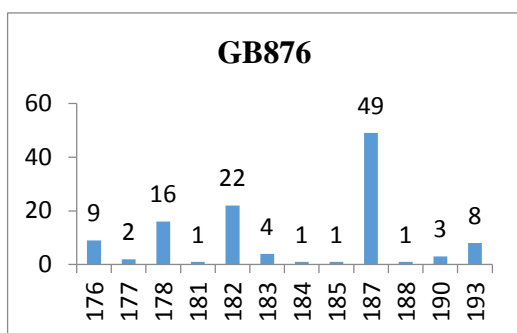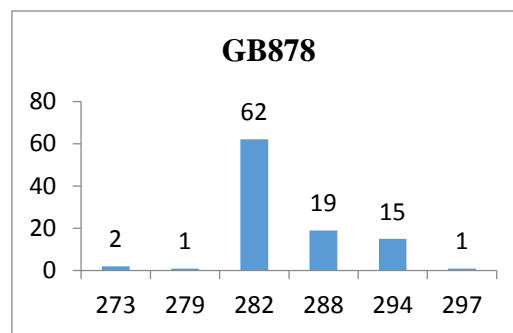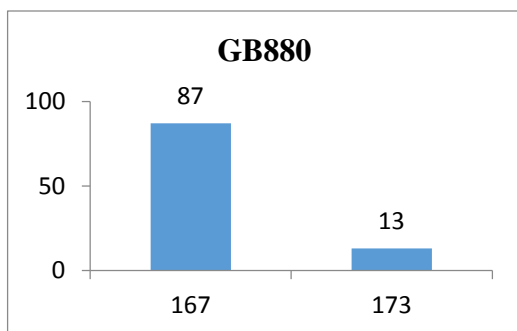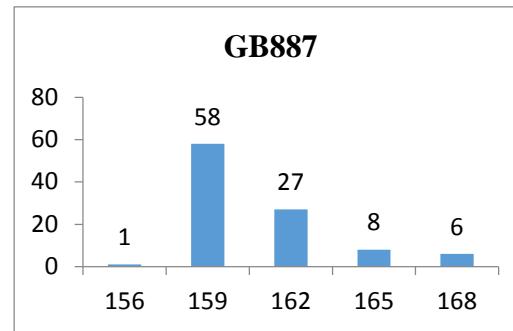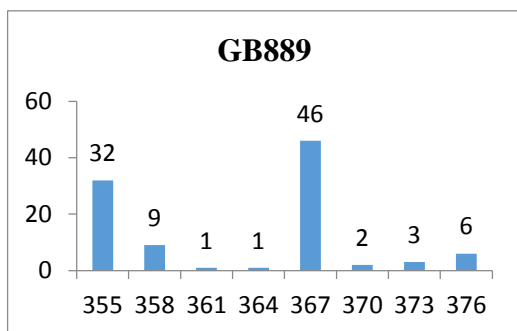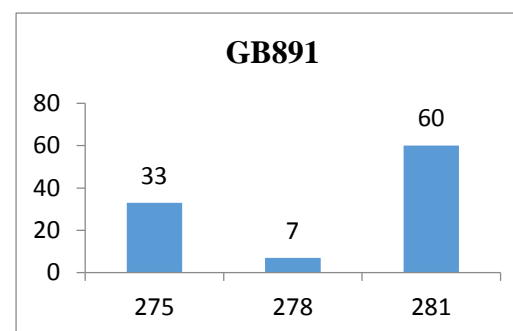

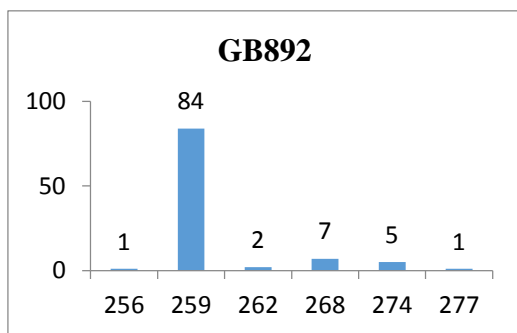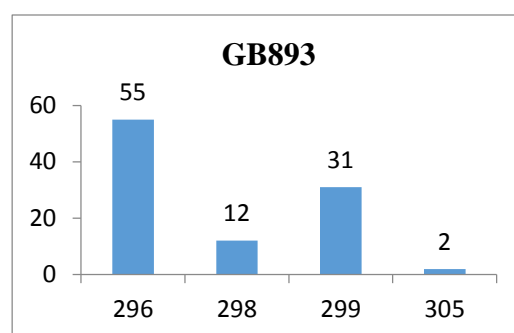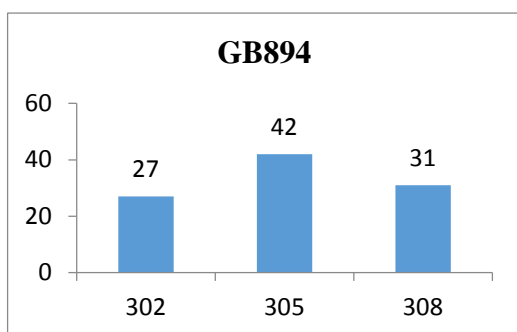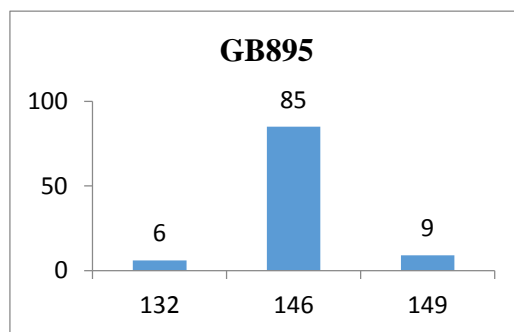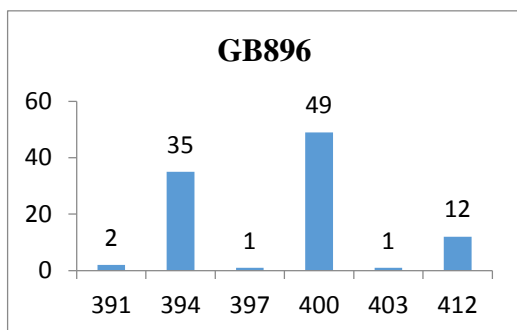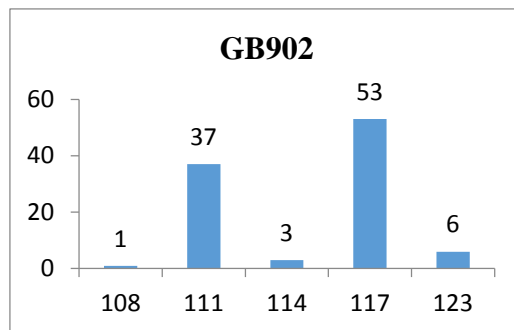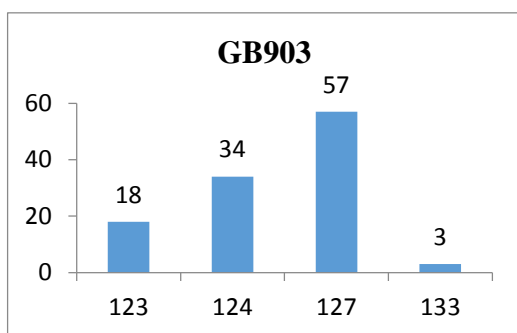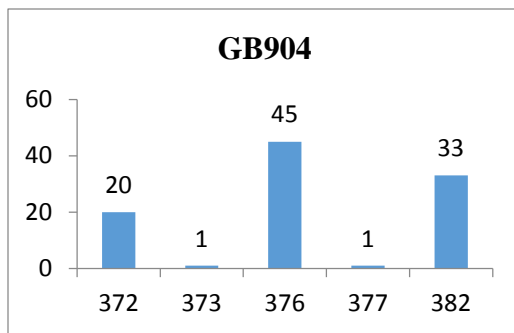

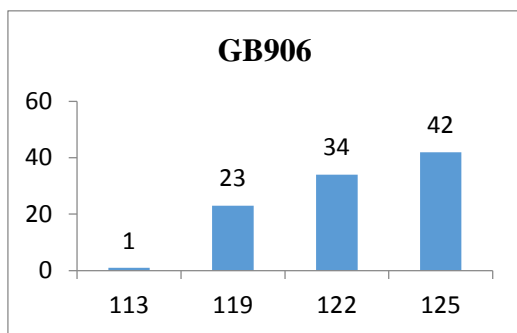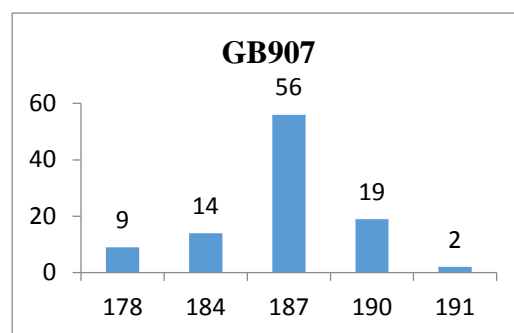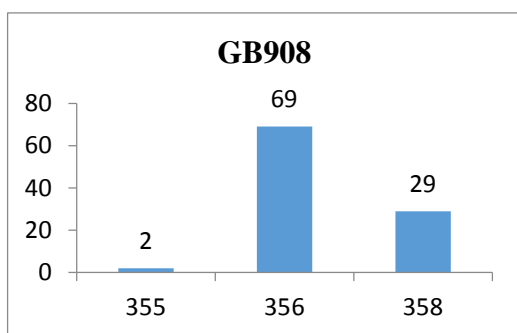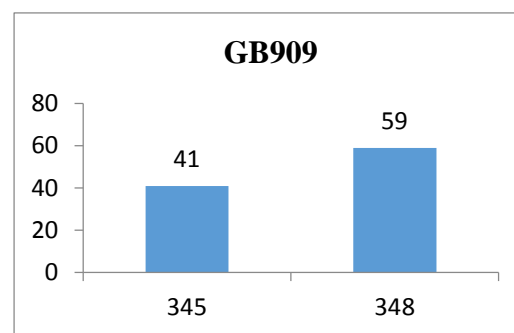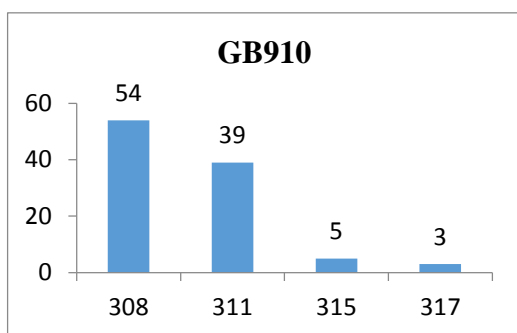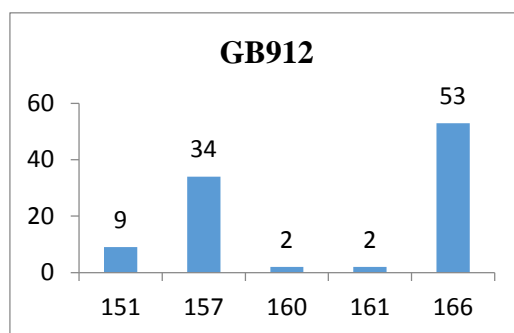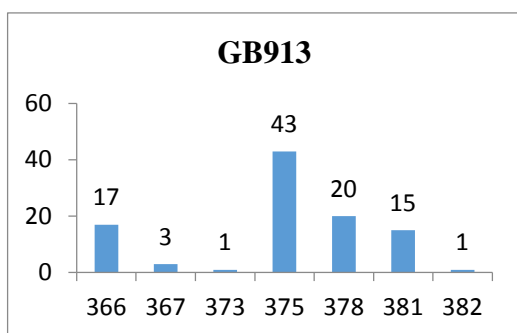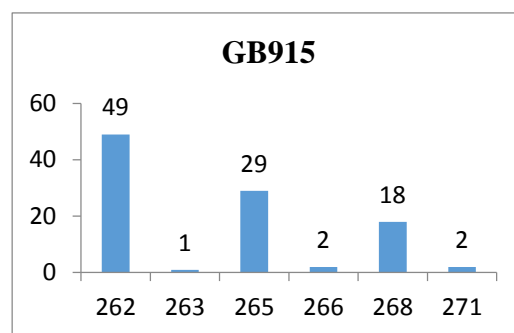

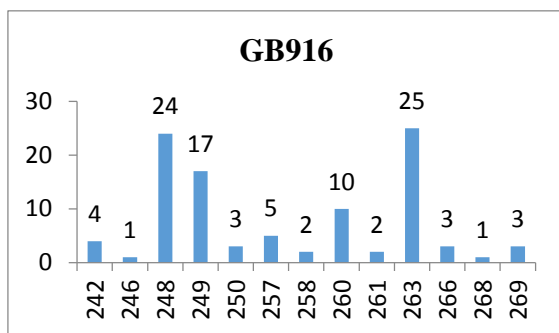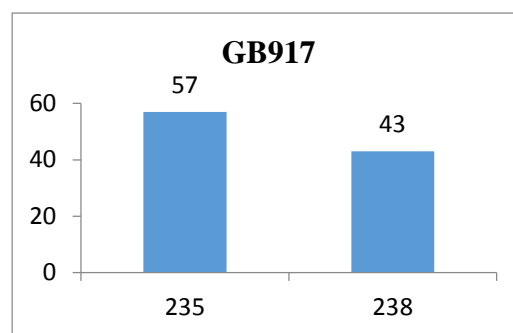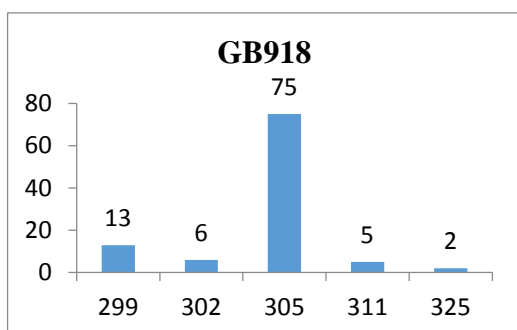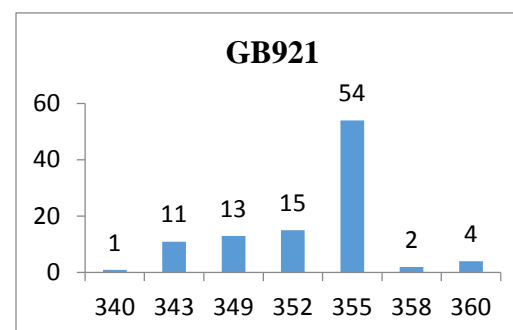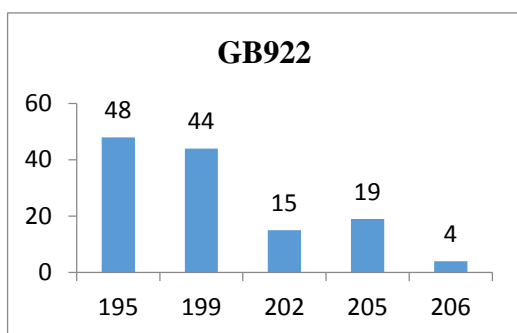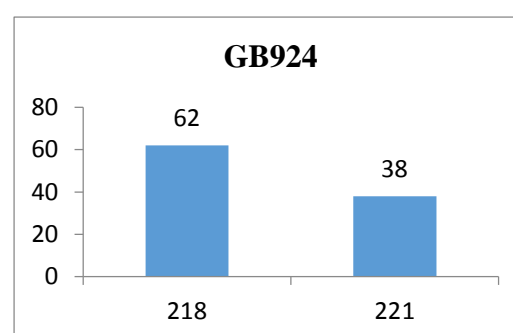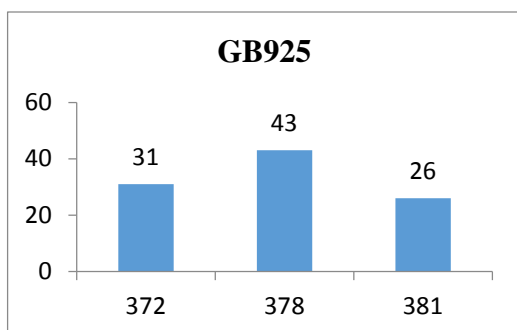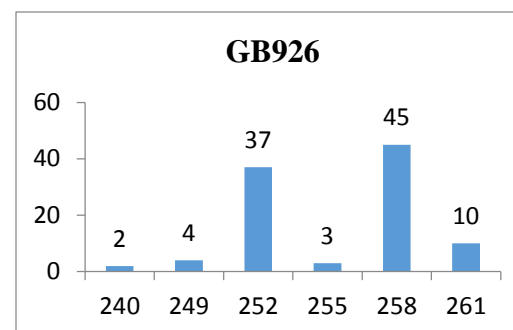

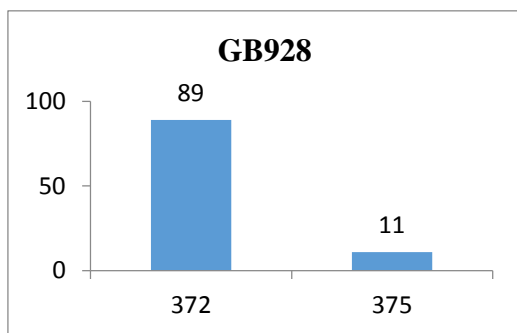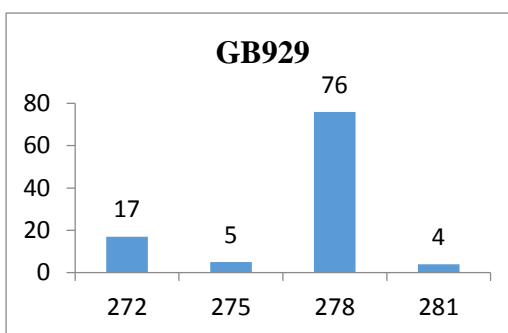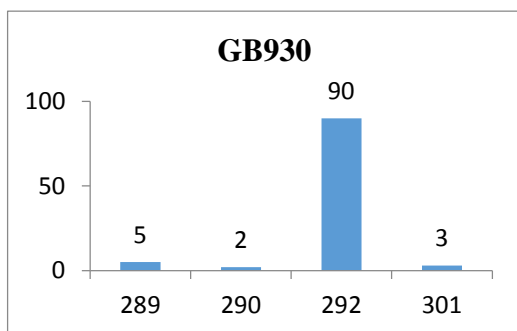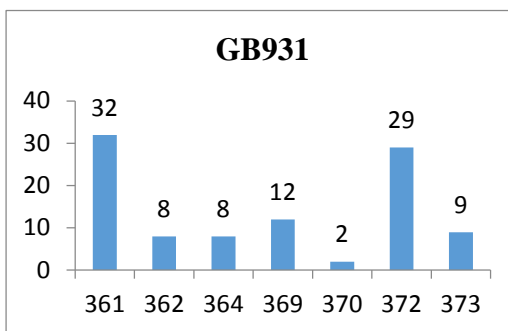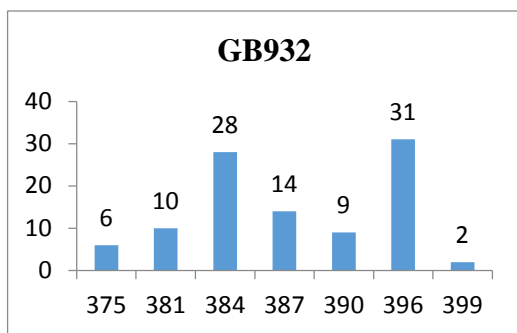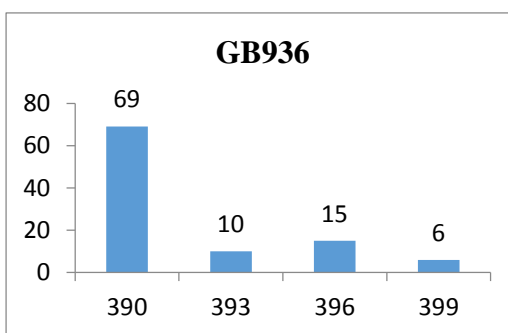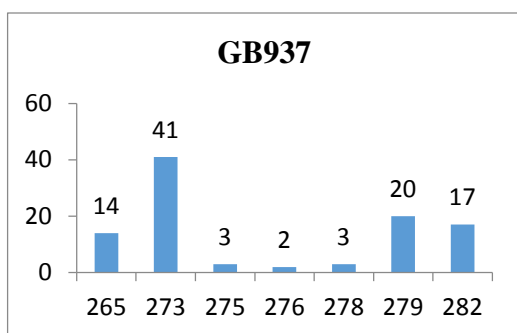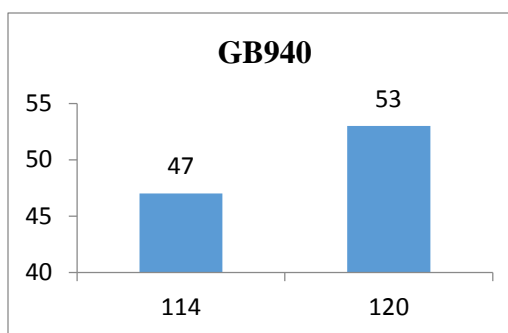

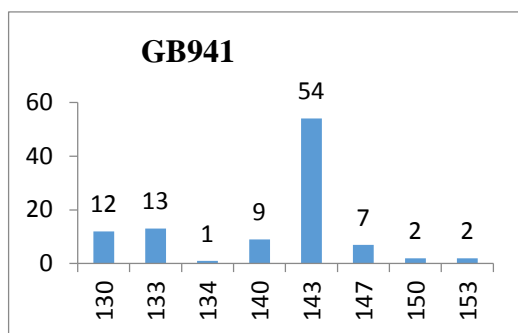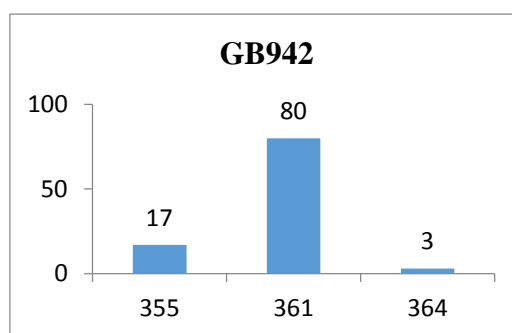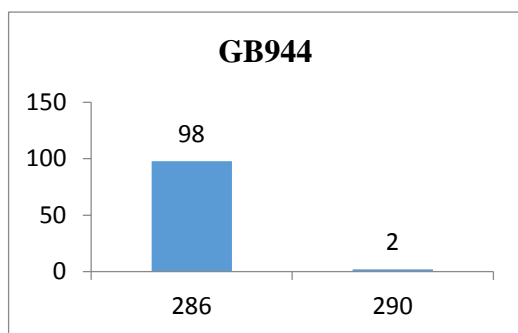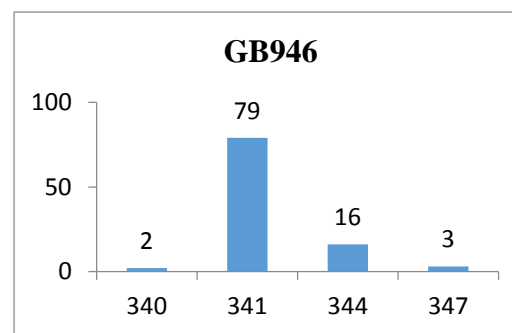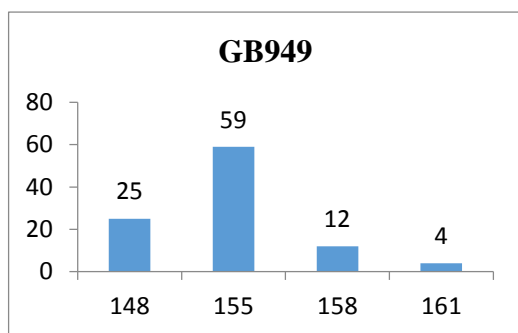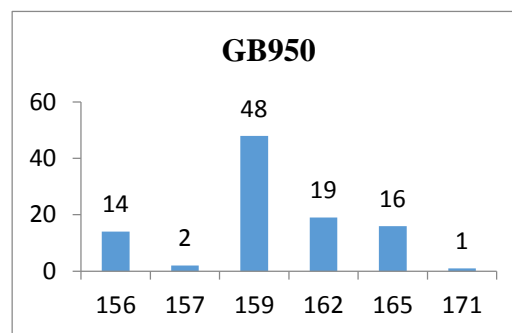

Supplement: S2 Fig — (PDF) [file pone.0178061.s002.pdf]
