# Supplementary material for: In silico development and characterization of tri-nucleotide simple sequence repeat markers in hazelnut (Corylus avellana L.)
Source: PLoS One. 2017 May 22;12(5):e0178061. doi: 10.1371/journal.pone.0178061 (PMC5439716; doi:10.1371/journal.pone.0178061)
Supplement: S1 Table — (PDF) [file pone.0178061.s003.pdf]

S1 Table. Characteristics and primer sequences of 150 new simple sequence repeat loci from the genome sequence of *Corylus avellana* 'Jefferson'.

Locus	GenBank accession no.	Repeat motif	Allele size	Ta (°C)	n	H_e	H_o	PIC	F(null)	Primers (5' - 3')
Loci with one or two bands in all 50 accessions.										
GB301	KT943758	$(ACC)_5$	216-234	59	7	0.573	0.50	0.523	0.063	[HEX]TCTGCCCACATCAGCTTAGATA<br>ATGGAAAGTGCTTAGAGCGAAT
GB302	KT943759	$(ACC)_5$	315-324	60	6	0.350	0.34	0.336	0.005	[FAM]TACCTCCTGTGCCACTTTCC<br>CTCCAAACCAGCTTCCTCAC
GB305	KT943760	$(ACC)_6$	217-226	60	4	0.150	0.16	0.143	-0.033	[HEX]TAACGGGGTGAACACTTAGGTC<br>CGGATGCTACATGGACAAGTTA
GB306	KT943761	$(ACC)_6$	237-249	60	6	0.546	0.50	0.509	0.036	[NED]GGTCACACCCTCACAAGTAACA<br>CTCGTCCTCATCCACAACATTA
GB307	KT943762	$(ACC)_7$	359-382	60	5	0.581	0.52	0.493	0.062	[HEX]TCCTCATGTCTATTGTTGCAGG<br>ATTGTGTATTGGAGGTTTTCGC
GB308	KT943763	$(ACC)_7$	279-290	60	9	0.718	0.78	0.681	-0.048	[NED]TGGGCTGTTTTTAACCCAAA<br>GTACACGGTGGGAGAGATCC
GB309	KT943764	$(ACC)_8$	277-287	61	3	0.167	0.14	0.158	0.081	[NED]CAAGGCAAAGTCAATGCAGA<br>GTGTGGTGGAATTGGTGGTC
GB310	KT943765	$(ACC)_8$	317-329	60	5	0.301	0.30	0.288	-0.021	[FAM]CGCCTTCCAGCTCTGTAAAA<br>TTGGATTCAGGATCCGATGT
GB313	KT943767	$(ACG)_6$	318-330	60	4	0.536	0.60	0.466	-0.048	[FAM]ATGATGGGCTTCTCATCCAA<br>CGGGGACTTGGAAATAGTGA
GB314	KT943768	$(AGC)_6$	126-129	58	2	0.484	0.66	0.367	-0.154	[HEX]TGTTTAGCTGATCGAATAGGGT<br>TGTGTGCGAAAGTAGAGAAATG
GB315	KT943769	$(AGC)_6$	129-135	60	2	0.420	0.44	0.332	-0.023	[HEX]GGGGATGGGCAAAGATAATAA<br>ATAACAAAACACAAGCCGCAG
GB317	KT943770	$(AGC)_7$	127-142	60	7	0.662	0.70	0.628	-0.031	[HEX]AATACAAACACCCCACTCCAAC<br>AGGGGAGCATAGGGAATACAAT
GB318	KT943771	$(AGG)_5$	103-112	60	3	0.594	0.58	0.517	-0.003	[HEX]GGGGTTGTCTACAGAGCAGAA<br>CATTGGGCTTGATCCATACA
GB319	KT943772	$(AGG)_5$	145-151	60	4	0.493	0.54	0.412	-0.052	[HEX]GGTAGGAGGGTTGGTGTTCA<br>GATGACGTTGGAAGCATTACAC
GB322	KT943773	$(AGG)_7$	111-120	60	4	0.333	0.34	0.299	-0.004	[HEX]AATGAAAGAGAAACGGAAGCG<br>ATCCTCCTCACTCAAACAAAGC
GB326	KT943777	(AGT)7	147-159	58	4	0.499	0.46	0.440	0.029	[HEX]TCTCTTCCTTTCTCTTCTCCTATCT<br>GCAGTATCAGTTCACAAAGCTG

S1 Table (cont'd). Characteristics and primer sequences of 150 new simple sequence repeat loci from the genome sequence of *Corylus avellana* 'Jefferson'.

Locus	GenBank accession no.	Repeat motif	Allele size	Ta (°C)	n	H_e	H_o	PIC	F(null)	Primers (5' - 3')
GB327	KT943774	$(CAC)_5$	307-325	60	4	0.185	0.20	0.178	-0.044	[FAM]GCCACGTGTAGTTTCACCAA<br>GGACCAATGGTGGAGGAAG
GB328	KT943775	$(CAC)_5$	141-144	60	2	0.493	0.52	0.371	-0.027	[HEX]AGGAAAGTGATTAGGTAAGTCTGGTAG<br>TTGAAATATGGGTAGGTGGGTT
GB329	KT943776	$(CAC)_5$	137-149	60	6	0.764	0.60	0.726	0.121	[FAM]AACTTCATGGCTTTTCCTTCC<br>ATCAGAGGGAGATCGAGAGATG
GB332	KT943778	$(CAG)_5$	275-292	60	5	0.615	0.76	0.542	-0.122	[FAM]CCCTTCTACACGCAACACAA<br>GGGCACTCTCACCAAACAAT
GB333	KT943779	$(CAG)_5$	334-355	60	7	0.645	0.68	0.580	-0.038	[HEX]ATTCACCTTCCATTGACCACAT<br>AGACCCACCAAAATTCACATTC
GB335	KT943780	$(CAG)_6$	383-395	60	3	0.185	0.20	0.176	-0.045	[FAM]GGGGATGGAAGAGATAAGACATT<br>ATGGTTGTGAAATTGGGTCTGT
GB338	KT943782	$(CAG)_7$	377-397	60	5	0.539	0.68	0.441	-0.120	[FAM]ACATCCCTATTATTCCAGCGTG<br>AGCTCTCCTCCTCCTATGCTCT
GB339	KT943783	$(CAG)_7$	291-309	60	7	0.539	0.58	0.516	-0.036	[FAM]GTACCCGTCTTCCTCATCCA<br>GCCGTACGATCTCTGTAGCC
GB340	KT943784	$(CAG)_8$	335-344	60	5	0.705	0.62	0.660	0.056	[HEX]CTTCCCATATCATCTTCGCTTC<br>TTCTGTTGTACCAGCATGTTCC
GB341	KT943785	$(CAG)_8$	358-367	60	3	0.545	0.56	0.441	-0.013	[HEX]ACAGAGAGGTTGGATGAATGCT<br>GCAAAAGGTTATGGTGAGGAAG
GB343	KT943786	$(CCA)_5$	337-340	60	2	0.365	0.44	0.298	-0.093	[HEX]GCTCGGTATCGAAAGAATGC<br>GCTCCAATCTGCTGGTCTTC
GB344	KT943787	$(CCA)_5$	160-163	60	2	0.180	0.16	0.164	0.059	[FAM]CCTCCACCATTGATTTCCAC<br>TTCAGGTCGTTTTCCGATTC
GB350	KT943789	$(CCA)_6$	143-158	60	4	0.483	0.36	0.412	0.155	[FAM]CCATAGCTACGCCTTTCACC<br>ACGTGGATATGGACGTGGAC
GB351	KT943790	$(CCA)_6$	181-193	60	5	0.635	0.64	0.576	-0.019	[FAM]ATTGCTATCTTGCCCTAACCAA<br>ACATCAAACCACCAACAATGAC
GB352	KT943791	$(CCA)_6$	376-379	60	4	0.500	0.64	0.394	-0.125	[HEX]AGAACCAGAACCAGAACCAAAG<br>CAACACACACCTCACCAAAAGT
GB353	KT943792	(CCA)6	368-374	60	3	0.165	0.16	0.155	0.060	[HEX]CCTTTACTTTCCCTCCTCTGCT<br>GATAGTCGGATACCAATCCCAA

S1 Table (cont'd). Characteristics and primer sequences of 150 new simple sequence repeat loci from the genome sequence of *Corylus avellana* 'Jefferson'.

Locus	GenBank accession no.	Repeat motif	Allele size	Ta (°C)	n	H_e	H_o	PIC	F(null)	Primers (5' - 3')
GB354	KT943793	$(CAC)_6$	273-282	60	4	0.662	0.64	0.592	0.025	[NED]ATTTTCCTTGGAGGGTTGCT<br>CCCCACCCAAATTTTGATAA
GB356	KT943794	$(CCA)_6$	357-361	60.5	3	0.649	0.52	0.574	0.112	[HEX]GAATGGTCTCACAGTTGTTTGC<br>GTGCAAGGCCAGTGAAGTTTAT
GB357	KT943795	$(CCA)_7$	377-394	60	11	0.764	0.80	0.733	-0.022	[FAM]TGTCCCTCCCAATACATTAACC<br>AGGAAACGACCACGAGAGATT
GB358	KT943796	$(CCA)_7$	364-375	60	4	0.517	0.52	0.464	-0.005	[HEX]GACAATAATAGGGTGGGCAGAT<br>GTCAGGTGGATGGAGGTGTATT
GB361	KT943797	$(CCA)_8$	180-189	60.5	4	0.675	0.68	0.613	-0.018	[FAM]GGAGAACTATGACTGCCCTGAT<br>AAATCCTGTTGTTGGTGGTGAT
GB365	KT943798	$(ATC)_7$	187-199	60	4	0.168	0.18	0.161	-0.039	[FAM]GGCTCTAGCATCAGCTTTTCAT<br>ATTGAACCTACCCATTCTGCTG
GB367	KT943799	$(CCT)_5$	86,-89	60	2	0.493	0.60	0.371	-0.098	[HEX]TTTGCACACCTACCAGAATCC<br>GAATACACGCTGAAGGAAAACC
GB372	KT943800	$(CCT)_5$	207-225	60	6	0.778	0.76	0.744	0.008	[HEX]TGCTAGCAGACGAGGGATTT<br>GCTCCGAATCCAATCACAGT
GB375	KT943801	$(CCT)_6$	183-200	61	4	0.561	0.52	0.474	0.037	[FAM]AAGCATCACCGGACCAATTA<br>CCATAAGCATCAACAACATAGCTG
GB376	KT943802	$(CCT)_6$	340-352	60	5	0.680	0.52	0.614	0.138	[HEX]CACCTGGAAAAGTCGAGCAT<br>TTGCTGCTCCAAAAAGGAGT
GB378	KT943804	$(CCT)_7$	173-185	59	5	0.654	0.64	0.614	0.006	[FAM]GCCTCTAAAGTTGGTTCTTTGC<br>AAGTCTCTTATTTGGGTGTGGG
GB381	KT943805	$(CCT)_8$	224-242	59	4	0.509	0.48	0.415	0.033	[HEX]GGCAGAAATGTACTATGATGCACT<br>TATTGTTGATGGGTTGTTCTCC
GB383	KT943806	$(CGA)_5$	198-201	59	3	0.516	0.54	0.402	-0.032	[FAM]GCTCATCTCAGCCTCGTATATGT<br>GCATTCTTCGTTGTTCTCATTC
GB386	KT943807	$(CGT)_5$	312-322	60	12	0.739	0.54	0.710	0.177	[FAM]ACCTTTTTGGGTGGGTTTTT<br>ATTGCCGCGTGTTTTGTTAG
GB387	KT943808	$(CGT)_5$	245-251	60	3	0.425	0.46	0.379	-0.061	[HEX]AACAGAGGGTTGAAGATTTGGA<br>CTAAGTACGAGAGAAGGGAAGGG
GB388	KT943809	$(CTC)_{10}$	224-242	60	6	0.581	0.60	0.545	-0.034	[NED]CGAAAGAGGAGGTACGAGAGAA<br>GGTAGCCGGTGGAGAAGAA

S1 Table (cont'd). Characteristics and primer sequences of 150 new simple sequence repeat loci from the genome sequence of *Corylus avellana* 'Jefferson'.

Locus	GenBank accession no.	Repeat motif	Allele size	Ta (°C)	n	H_e	H_o	PIC	F(null)	Primers (5' - 3')
GB390	KT943810	$(CTC)_5$	314-318	60	3	0.470	0.46	0.417	0.030	[FAM]AACAAACTCCAGCGGACATC<br>TCAGTGGAGCTGTTGAGCTG
GB393	KT943811	$(CTC)_5$	231-246	60	4	0.461	0.26	0.379	0.293	[HEX]CCCTTAGGGCCACCATTATT<br>CCTTAGGCCACTCCCAAAAT
GB394	KT943812	$(CTC)_6$	255-261	60	2	0.412	0.30	0.327	0.157	[NED]TCTTGAGCCTGCTTCGAACT<br>TTCTACGGGGATTTTTGTCG
GB395	KT943813	$(CTC)_6$	370-388	60	7	0.803	0.78	0.775	0.010	[FAM]TGTTATTTTCTCATCTGCGTGG<br>CTCTGATCGAACCAACCATGT
GB801	KT943814	$(CTC)_9$	289-297	59	4	0.499	0.44	0.440	0.076	[FAM]TTCTGATAATGCTGATGTGGCT<br>GTGCAATAGAAAGTGACCAGGA
GB802	KT943815	$(CAG)_6$	266-275	60	7	0.766	0.90	0.728	-0.082	[NED]TTTAAGCCTACAACTACAAGTGGC<br>GATCACTCCACCCTCGTCA
GB808	KT943817	$(CTG)_7$	362-374	60	5	0.539	0.54	0.455	0.001	[FAM]GCATAAACCACTCCAACTCCTC<br>TTTGCTATCCCTACTCAGCTCC
GB809	KT943818	$(CTG)_7$	394-397	60	2	0.241	0.24	0.212	0.002	[FAM]TTCCATCTCATAGCCATTGTTG<br>GAACTATTGGAGAACTCGGGTG
GB810	KT943819	$(CTG)_7$	345-357	60	5	0.620	0.68	0.576	-0.041	[HEX]AGAAAAGAGCAGGAAGTTGCAC<br>TTGAGACAAAGCAAGGGAGATT
GB812	KT943820	$(CTG)_8$	352-358	60	4	0.558	0.58	0.464	-0.026	[HEX]GGGCTTTGAGAGTTGGATATGA<br>CAGCCGTCACTTACCTACCTG
GB813	KT943821	$(GAC)_5$	371-375	60	7	0.665	0.50	0.605	0.130	[HEX]CGTAATGAGAGGTAGGGAAGGA<br>CCCAAAACCAATAACCACATTC
GB814	KT943822	$(GAC)_6$	370-385	60	5	0.658	0.62	0.612	0.023	[HEX]GGTCGTCCTTGGTTTTGTAGC<br>CAGATGCTCAGTTGCGGATA
GB817	KT943824	$(GAC)_8$	359-374	61	7	0.464	0.52	0.446	-0.102	[HEX]GCACCCATTAGACCTCCAAATA<br>CTTACCTCCCAACGCCTCTT
GB818	KT943825	$(GAG)_5$	129-144	60	5	0.713	0.68	0.662	0.023	[HEX]GAAGTTGGGTTGGAAGCAGTT<br>CGTCCTCTGCACACTCTCATAC
GB819	KT943826	$(GAG)_5$	145-151	60	3	0.477	0.42	0.419	0.089	[HEX]ATTCAAGAGCAACCCACAATCT<br>CTCAACATGCACCACAAAATCT
GB821	KT943827	$(GAG)_5$	152-170	60	7	0.494	0.14	0.454	0.562	[FAM]TGGGAACACAAAAAGGGAAA<br>TCTCAAGTGGAGAGGCTTGC

S1 Table (cont'd). Characteristics and primer sequences of 150 new simple sequence repeat loci from the genome sequence of *Corylus avellana* 'Jefferson'.

Locus	GenBank accession no.	Repeat motif	Allele size	Ta (°C)	n	H_e	H_o	PIC	F(null)	Primers (5' - 3')
GB823	KT943829	$(GAG)_5$	142-160	60	9	0.845	0.70	0.825	0.094	[HEX]GGTGTCTCAAAACAACAGACGA<br>GTTGGAGTTCATCCCAGGAC
GB826	KT943831	$(GAG)_6$	203-221	60	6	0.643	0.70	0.597	-0.052	[HEX]CGTAACCTTCCCCTCTGACA<br>TCTCCAAACCATTCCTGCTT
GB828	KT943833	$(GAG)_7$	108-117	60.7	3	0.299	0.36	0.260	-0.096	[HEX]ACATTCGGTTCGGGTTTTC<br>ATCCAACACCAATCTGGGC
GB829	KT943834	$(GAG)_7$	141-148	60	3	0.199	0.22	0.186	-0.051	[HEX]ATCTGCCCAAGAAGCGTG<br>CCATCAATTCTCGTCTTATCTCTTC
GB831	KT943835	$(GAG)_7$	127-145	60	6	0.694	0.80	0.642	-0.074	[HEX]TGGCCCTCGTAGAATAAGATTG<br>CAACCAAGAAATGACCCTCTTC
GB832	KT943836	$(GAG)_8$	185-203	58	12	0.777	0.70	0.759	0.058	[HEX]AAGTGTGGCAATTCAGGC<br>GGCAGCTTCTCTCTTTGTAATC
GB834	KT943837	$(GAG)_8$	145-161	60	4	0.218	0.22	0.207	0.019	[FAM]GTGGGATTCGGACAATAAACC<br>CGACAGGTTCAAGAGGGTAATC
GB835	KT943838	$(GAG)_8$	153-171	60	7	0.522	0.36	0.485	0.181	[HEX]TTGGGAATATCACTCTCATCCA<br>GCGTTCAAAGGAGTCAAAAGTC
GB836	KT943839	$(CAG)_5$	193-196	60	2	0.476	0.42	0.363	0.062	[HEX]ATTTCTCCTCTACCGAACCCAT<br>TTTCCTGTTCTTGTTGTCGTTG
GB838	KT943840	$(GCA)_5$	168-182	59	3	0.443	0.44	0.388	-0.002	[FAM]AAGATAAGGCAGACGACAGACA<br>GAATAGAAATGCCAATCACCAG
GB840	KT943842	$(AGC)_6$	155-191	60	7	0.271	0.30	0.261	-0.073	[FAM]AAGCTAAACCACCATATCTCCG<br>GCGATGAATTTGCTCTTCTTG
GB841	KT943843	$(GCA)_6$	185-194	60.3	2	0.241	0.28	0.212	-0.072	[HEX]CCTAGCCGTAGAACATCTGCTT<br>ATGAATGCTTGCTTGAGTGTTG
GB843	KT943844	$(GCG)_5$	173-185	60	3	0.363	0.32	0.310	0.072	[FAM]GCAAAACATGTGCCAACAAC<br>TGCTCAGGCTTGTACAGGTG
GB847	KT943846	$(GCT)_5$	177-181	60	4	0.582	0.62	0.495	-0.030	[FAM]TTCCCATTCCCATTAACGTC<br>GCGACCGAGAGAGAGAGAGA
GB850	KT943847	$(GCT)_7$	213-231	60	7	0.515	0.62	0.454	-0.096	[HEX]TACCACCACAACAACAACATCA<br>AGCATGACGACACATCTCCTAA
GB851	KT943848	$(GCT)_8$	198-226	60	6	0.609	0.52	0.559	0.072	[HEX]ATCATCCTTCCCCTTGGACTTA<br>ACTCTGCCTATGTACCACCCAG

S1 Table (cont'd). Characteristics and primer sequences of 150 new simple sequence repeat loci from the genome sequence of *Corylus avellana* 'Jefferson'.

Locus	GenBank accession no.	Repeat motif	Allele size	Ta (°C)	n	H_e	H_o	PIC	F(null)	Primers (5' - 3')
GB852	KT943849	$(GCT)_8$	288-315	60	5	0.679	0.68	0.627	0.007	[FAM]TGCTCTTTCTGGGTTTGCTT<br>CAGAAGCATCATCGACAGGA
GB853	KT943850	$(GGA)_5$	180-189	60	6	0.347	0.30	0.330	0.069	[HEX]TCACATCATTGGGAACATCG<br>CCCGGATTTGGATGATACAG
GB854	KT943851	$(GGA)_5$	203-218	60	4	0.169	0.18	0.164	-0.038	[HEX]GCAAGGCTGCGAATTATGTAGT<br>CATACCCAGTTTTACGATGCAA
GB855	KT943852	$(GGA)_5$	234-240	60	4	0.201	0.18	0.190	0.045	[NED]GGAGCAGAGGAAGATGGAGA<br>AAGCCTTTGCAGCCATAGAA
GB856	KT943853	$(GGA)_5$	253-258	60	7	0.522	0.32	0.483	0.270	[NED]GTGCTTGATATTGGCTCTCACC<br>CTAGCTCATACACATTCAACAATGC
GB858	KT943854	$(GGA)_5$	327-333	60	3	0.499	0.04	0.384	0.854	[FAM]ATTGAATGCTCGTTGCACTG<br>TGGACAAGAAACGCTCAAAA
GB860	KT943855	$(GGA)_5$	293-302	60	3	0.393	0.42	0.358	-0.012	[FAM]ACCGCTATTACTGACCACCTTG<br>GACAGAGAAAGAGAGATGGGGA
GB865	KT943856	$(GGA)_6$	259-268	60	4	0.415	0.38	0.389	0.046	[NED]TCAAAGGGTTATTCAGAGGGTG<br>ACTTCTGCTTCTATGTACCGCC
GB866	KT943857	$(GGA)_6$	246-252	60	5	0.677	0.46	0.614	0.202	[NED]GCTTGTGGGTGTTGGTAAAGAT<br>TCATTCCTTTGCTTTGTCACTC
GB867	KT943858	$(GGA)_6$	286-301	60	5	0.429	0.44	0.404	-0.017	[FAM]CTTGGCAAAGCTACCCTCAC<br>ACGCGTTCTCTCCTAACGAA
GB868	KT943859	$(GGA)_6$	288-300	60	5	0.679	0.76	0.623	-0.075	[FAM]GAGAGCCACTGAAGCGGA<br>CACAAGGAAAGCACACTAGCAG
GB869	KT943860	$(GGA)_7$	184-196	60	3	0.167	0.18	0.158	-0.039	[HEX]AGCCGAAAAACCATTGATGA<br>AAAGCCCAGGGACAAAGATT
GB870	KT943861	$(GGA)_7$	318-324	60	3	0.524	0.42	0.430	0.126	[FAM]TTTTGCTTGTGTAGGGAACCTT<br>ATATGGAACTGCATGGGTCTTT
GB871	KT943863	$(GGA)_8$	329-341	60	5	0.437	0.42	0.378	0.007	[FAM]ATGACAAACTTAATGCCGTTGG<br>CTTCGCTGAAATCGTTCCAT
GB873	KT943862	$(GGA)_7$	268-279	60	5	0.449	0.44	0.416	0.030	[NED]CTTGCAGGTGATGAGGATGA<br>GGAAACTTAGCAGCTAGCAATGA
GB875	KT943864	$(GGA)_9$	325-349	60	8	0.676	0.72	0.620	-0.038	[FAM]ATGATGATGAGGAGGAGGAGAA<br>CAAAATCAGGCATACAGAACCA

S1 Table (cont'd). Characteristics and primer sequences of 150 new simple sequence repeat loci from the genome sequence of *Corylus avellana* 'Jefferson'.

Locus	GenBank accession no.	Repeat motif	Allele size	Ta (°C)	n	H_e	H_o	PIC	F(null)	Primers (5' - 3')
GB878	KT943866	$(GGT)_{10}$	273-297	60	6	0.556	0.68	0.509	-0.109	[NED]ATCAACGCACTCCACATGAA<br>ACGCCAAGAAAGAAGACAGC
GB880	KT943867	$(GGT)_5$	167-173	60	2	0.226	0.26	0.201	-0.065	[FAM]TTGCTGCTGTGTAGGGATCA<br>CCTCCTCCAAAACCCTAACC
GB887	KT943868	$(GGT)_5$	159-168	60	5	0.581	0.60	0.523	-0.020	[FAM]AGACTCGTTGGTGCTGCTTT<br>AACCCTGAACCATACCCTGA
GB889	KT943869	$(GGT)_7$	355-376	60	9	0.682	0.64	0.632	0.031	[HEX]CTCTCTGATTGGTGGAGCAAG<br>TCTCTCTCTAGTTCGGCATCAA
GB891	KT943870	$(GGT)_9$	275-281	60	3	0.526	0.42	0.443	0.107	[FAM]CCCTCTGAGCTTGTGAAGGA<br>CGAAGCGTCTCCTGGAATAC
GB892	KT943871	$(GTG)_5$	250-275	60	6	0.286	0.28	0.275	0.040	[NED]GGAAAGCCTGTTTTTGGTGA<br>TGGACTACAAGCTGCCACTG
GB893	KT943872	$(GTG)_5$	296-305	60	5	0.587	0.62	0.517	-0.026	[FAM]CGATGAGACCATGTTGATGC<br>TCTCTCCACAAACCCACCAT
GB894	KT943873	$(GTG)_5$	302-308	60	3	0.655	0.46	0.581	0.177	[FAM]TTGCAAAAGCTTTGTTGATGA<br>TTCAAAATGACTGCCCAACA
GB895	KT943874	$(GTG)_5$	132-149	62	3	0.266	0.28	0.249	-0.014	[HEX]TGTATGGGACACGGGTGG<br>ACCAACAAGAGCCAACCTTCAT
GB896	KT943875	$(GTG)_5$	391-412	61	9	0.634	0.54	0.569	0.084	[FAM]CTTAGATCATTCGCCCTAGCC<br>TGAAGTAGATCCCCAAGCAGAG
GB902	KT943876	$(GTG)_6$	108-123	60	5	0.578	0.24	0.497	0.426	[HEX]CTCCCGCAAATGAAATGTG<br>AAGGAGGATCCTTGAAGAGG
GB904	KT943878	$(GTG)_7$	372-377	60	5	0.648	0.64	0.579	0.006	[HEX]TCCGAAGCTCACTATGAATGAA<br>AGAGAAGGGGACCTGAGAAACT
GB906	KT943879	$(GTG)_7$	113-125	60	5	0.659	0.62	0.590	0.049	[HEX]CTCAGCGAAGAAGATGTGTTGT<br>GTCATACCCACGTTTTGCATAC
GB907	KT943880	$(GTG)_9$	178-190	60	6	0.633	0.68	0.592	-0.028	[FAM]GGGGATAGTTGGACCACTCC<br>ATTGCACAACTTGCCAACAA
GB908	KT943881	$(TCC)_5$	356-358	59	3	0.439	0.46	0.359	-0.032	[HEX]ACAATATCGGTGAAGAGGTCAA<br>GATGGGGTGATGCAGTTATTAG
GB909	KT943882	$(TCC)_5$	345-348	60	2	0.484	0.54	0.367	-0.055	[HEX]CGTAAGAAAAGGCGAAGACG<br>CCCTTCTCTCCACTCCCTTC

S1 Table (cont'd). Characteristics and primer sequences of 150 new simple sequence repeat loci from the genome sequence of *Corylus avellana* 'Jefferson'.

Locus	GenBank accession no.	Repeat motif	Allele size	Ta (°C)	n	H_e	H_o	PIC	F(null)	Primers (5' - 3')
GB912	KT943884	$(TCC)_5$	157-168	60	5	0.595	0.44	0.523	0.137	[FAM]ATTGCTGCTGCTGCTGATAA<br>TACGAGTTTGCTCCCGAGAT
GB913	KT943885	$(TCC)_5$	367-382	60	7	0.723	0.52	0.683	0.159	[FAM]ACCACAATGATCGGTCCACT<br>CTGAAGAGCTGGAGGTTTTGTT
GB916	KT943887	$(TCC)_6$	240-267	60	13	0.833	0.82	0.814	0.008	[NED]TAAAGGTGCACAAGCCACTG<br>TTCAAGAAAGCGGGAAAGAA
GB917	KT943888	$(TCC)_7$	235-238	60	2	0.490	0.46	0.370	0.032	[NED]TCCCAAGAAAATCGAGGATG<br>GAGGGAGACGACGAGAGAGA
GB921	KT943890	$(TCC)_8$	340-367	60	7	0.655	0.68	0.622	-0.031	[HEX]TGCATCCTGAGCATCGTAAG<br>CCATGGCTTGTAAGCAAGAA
GB924	KT943892	$(TGC)_5$	218-221	60	2	0.471	0.48	0.360	-0.009	[HEX]TGCGTAGCAGATGGTCAGAG<br>CATTATGGCTCCTGGTGCTT
GB925	KT943893	$(TGC)_5$	372-396	60	3	0.651	0.54	0.578	0.093	[FAM]TTAAGCAACATCCGCAAGTG<br>AAAAGAAAAGAGAGGTCCCCAT
GB928	KT943895	$(TGC)_5$	372-375	60	2	0.196	0.22	0.177	-0.053	[FAM]CAAACCCTCCACACAGATATGA<br>AAAAGCACCTCTTAGCCTCCTT
GB930	KT943897	$(TGC)_5$	289-301	60	4	0.186	0.16	0.180	0.064	[NED]GATGGGACAAGACATGGTCA<br>CGCAGGGAAGAAAGAAACAG
GB931	KT943898	$(TGC)_5$	361-373	59	7	0.778	0.68	0.746	0.074	[FAM]GCATTCGGGTAAACACATACTC<br>TTGAAGGAGCAGATGACCC
GB932	KT943899	$(TGC)_6$	375-399	60	7	0.784	0.74	0.753	0.032	[FAM]TGAAAGGTATGTTGCTGTTGCT<br>CAAGGAATGGGTTTGTTACCAT
GB936	KT943900	$(TGC)_7$	390-399	60	4	0.488	0.52	0.453	-0.056	[FAM]TTCTTTGTCATCTGAAGTGGGA<br>GCAGCCATCTATTCAAAACCAT
GB937	KT943901	$(TGC)_7$	273-282	60	7	0.741	0.74	0.705	0.003	[NED]AGCTGCAATCCAACAACTCC<br>GGAGTGAAAGTACGGCAACC
GB940	KT943902	$(TGC)_7$	113-127	60	2	0.498	0.42	0.374	0.085	[HEX]AATCTCCAATCCTGCTGAACTC<br>GCTTGGGCTAGAAAGTTATGCT
GB941	KT943903	$(TGC)_{11}$	130-153	60	8	0.663	0.56	0.635	0.101	[HEX]ATGCTGCTGGGGTTTCTG<br>TCTCTATTTGTTCCGTGTCTGC
GB942	KT943904	$(TGG)_5$	355-364	60	3	0.330	0.34	0.292	-0.006	[FAM]CGAACCTCAAATACCAGGAAAG<br>GGCACAATGCTTACCAAAAGA

S1 Table (cont'd). Characteristics and primer sequences of 150 new simple sequence repeat loci from the genome sequence of *Corylus avellana* 'Jefferson'.

Locus	GenBank accession no.	Repeat motif	Allele size	Ta (°C)	n	H_e	H_o	PIC	F(null)	Primers (5' - 3')
GB944	KT943905	$(TGG)_5$	284-290	60	2	0.039	0.04	0.038	-0.003	[NED]TCATCACCTGAAACAAGCTGTC<br>CTAGCCCTACACCTGAGAGAGG
GB946	KT943906	$(TGG)_6$	341-347	60	4	0.349	0.34	0.315	-0.006	[HEX]GTTCGAGTTTCAACCACACTGA<br>GCTCATTTCCATATTCTCCACC
GB949	KT943907	$(TGG)_7$	148-161	60	4	0.573	0.52	0.517	0.042	[FAM]TTTGGAGGGAGACAGTTTGG<br>GGTTGGCCAAGAATGAGAGA
GB950	KT943908	$(TGG)_7$	156-171	60	6	0.688	0.66	0.646	0.018	[HEX]GAAGAAGACGAGGAGCACATTT<br>ACTGAGCATTTCCAACCCATAC
Mean					4.73	0.509	0.486	0.457	0.042	
Loci with three or four bands in a few accessions.										
GB311	KT943766	$(ACC)_8$	273-288	60	5	--	--	--	--	[NED]AGAGTGTAACCTGTGGACGCTT<br>TCAACCAACATTATAGGCTCCC
GB346	KT943788	$(CCA)_5$	349-372	60	9	--	--	--	--	[HEX]ATGGAGCTATTTGACCATCACC<br>CAATGGAAGAGGGTATCCAAGT
GB377	KT943803	$(CCT)_6$	115-124	60	5	--	--	--	--	[HEX]AAACAGAGCAGGAAGAGTGAGG<br>ATTTAGGGGTAGGCAGGTTTGT
GB807	KT943816	$(CTG)_7$	212-215	60	3	--	--	--	--	[HEX]TGAATTGAAAAGGGGCTACG<br>GGGCAGTCAATCCTTGAGAG
GB815	KT943823	$(GAC)_6$	236-245	60	4	--	--	--	--	[HEX]GAATCTCAGCCCTTCAATGC<br>CGCATGTAGAGTCCCAAACA
GB822	KT943828	$(GAG)_5$	213-225	60	6	--	--	--	--	[HEX]CCTCAATCACCAAGCTCTGC<br>CCATCAACCAATGCTCCTCT
GB824	KT943830	$(GAG)_5$	123-135	61	4	--	--	--	--	[HEX]TGAAGAGGACGAGGAAAGAGAG<br>GGAACTTTACCGCCAAATCAG
GB827	KT943832	$(GAG)_6$	214-223	60	5	--	--	--	--	[HEX]GTAGGGCTTCGAGATCAACG<br>TCTCCTGCCTCTCTGTCCAT
GB839	KT943841	$(CAG)_5$	244-262	60	4	--	--	--	--	[NED]TTTGCCACTGCATCTGAGTC<br>TGCTGAAAGGATGGTTGTTG
GB845	KT943845	$(GCT)_5$	191-230	60	7	--	--	--	--	[HEX]CAAGTATCTGTGCCCATTGC<br>TTTCTTCCATTTCCCTTCCA
GB876	KT943865	$(GGC)_5$	176-193	60	12	--	--	--	--	[FAM]GGGAAGAAGGATTCCCTCAG<br>CCCAACTCCCTCATAAGCAA

S1 Table (cont'd). Characteristics and primer sequences of 150 new simple sequence repeat loci from the genome sequence of *Corylus avellana* 'Jefferson'.

Locus	GenBank accession no.	Repeat motif	Allele size	Ta (°C)	n	H_e	H_o	PIC	r	Primers (5' - 3')
GB903	KT943877	$(GTG)_6$	114-123	60	4	--	--	--	--	[HEX]TGAGTGGTGATAGTGAGAGAGCA<br>CGACATTTAAGAAGTGGGGTTC
GB910	KT943883	$(TCC)_5$	308-317	60	4	--	--	--	--	[FAM]CGGCTGCCTCTTCACCTAT<br>TTTGTGGCCAATTCCTCTTC
GB915	KT943886	$(TCC)_6$	262-268	60	6	--	--	--	--	NED]TCCAAGGTGGCAATTCATAA<br>AGTTGAGGGCAAAGACATGG
GB918	KT943889	$(TCC)_7$	299-311	60	5	--	--	--	--	[FAM]ATCAACCGGCGGACTAGAG<br>GTCAGCCATGGGGAATTAGA
GB922	KT943891	$(TCC)_8$	196-216	60	5	--	--	--	--	[HEX]TATCCTCTTCCCACGTGTCC<br>GCGGAGTTGTCAAAAAGAGC
GB926	KT943894	$(TGC)_5$	241-274	60	6	--	--	--	--	[NED]GAGGTCTTACGCAATGTTGGAT<br>CTCCCTCTCTCTTTGGTCACAC
GB929	KT943896	$(TGC)_5$	272-281	60	4	--	--	--	--	[NED]GGGCTATGGAAGGATCATCA<br>AACGCAAGAACAAAGGGAGA
