# Supplementary material for: In silico development and characterization of tri-nucleotide simple sequence repeat markers in hazelnut (Corylus avellana L.)
Source: PLoS One. 2017 May 22;12(5):e0178061. doi: 10.1371/journal.pone.0178061 (PMC5439716; doi:10.1371/journal.pone.0178061)
Supplement: S2 Table — (PDF) [file pone.0178061.s004.pdf]

S2 Table. Segregation at new tri-nucleotide simple sequence repeat marker loci in the hazelnut reference mapping population (OSU 252.146 x OSU 414.062).

Locus	Alleles (Female x Male)	Expected	Observed	χ2	P	LG and alleles
GB301	222/228 x 218/222	1:1:1:1	27:44:36:31	4.67	0.20	218 on 9R, 228 on 9S
GB302	318/318 x 318/324	1:1	70:66	0.12	0.73	324d on 4R
GB307	368/371 x 368/371	1:2:1	34:63:41	1.76	0.42	371 on 7R, 371 on 7S
GB309	279/287 x 279/279	1:1	67:70	0.07	0.80	287 on 5S
GB310	320/320 x 320/326	1:1	69:62	0.37	0.54	326 on 6R
GB313	318/321 x 318/321	1:2:1	36:61:41	2.22	0.33	321 on 8R, 318 on 8S
GB314	126/129 x 126/129	1:2:1	43:60:35	3.28	0.19	126 on 8R, 129 on 8S
GB315	129/135 x 129/135	1:2:1	7:62:68	54.97	0.00	129 on 11R, 135 on 11S
GB317	136/136 x 136/139	1:1	69:69	0.00	1.00	139d on 8R
GB318	103/106 x 103/112	1:1:1:1	31:27:39:38	2.93	0.40	106 on 9S, 112 on 9R
GB319	145/148 x 145/148	1:2:1	40:60:30	1.48	0.48	145 on 7R, 145 on 7S
GB326	150/150 x 150/156	1:1	60:77	2.11	0.15	156 on 4R
GB327	316/325 x 316/316	1:1	58:79	3.22	0.07	325d on 1S
GB328	141/144 x 144/144	1:1	58:74	1.94	0.16	141 on 11S
GB329	143/143 x 140/151	1:1	65:71	0.27	0.61	151 on 2R
GB332	283/286 x 283/286	1:2:1	29:76:33	1.65	0.44	283 on 9R, 283 on 9S
GB333	343/343 x 343/352	1:1	19:119	72.46	0.00	unlinked
GB338	380/388 x 388/388	1:1	56:80	4.24	0.04	380 on 11S
GB339	303/303 x 303/306	1:1	70:55	0.19	0.67	306 on 9R
GB340	341/344 x 341/344	1:2:1	44:66:27	4.30	0.12	341 on 8R, 344 on 8S
GB341	361/367 x 361/367	1:2:1	40:63:33	1.17	0.56	367 on 2R, 367 on 2S
GB343	337/340 x 337/340	1:2:1	66:62:8	49.48	0.00	340 on 11R, 337 on 11S
GB346	360/null x 356/371	1:1:1:1	43:35:32:23	6.16	0.10	371 on 1R, null on 1S
GB350	143/158 x 155/155	1:1	63:74	0.88	0.35	158 on 1S
GB351	181/181 x 181/190	1:1	63:75	1.04	0.31	190d on 5R
GB354	279/279 x 273/279	1:1	110:27	50.28	0.00	unlinked
GB357	385/393 x 385/388	1:1:1:1	17:41:26:54	23.22	0.00	393 on 11S
GB358	367/370 x 367/370	1:2:1	35:66:34	0.01	0.99	367 on 1R, 367 on 1S

S2 Table (cont'd). Segregation at new tri-nucleotide simple sequence repeat marker loci in the hazelnut reference mapping.

Locus	Alleles (Female x Male)	Expected	Observed	χ2	P	LG and alleles
GB361	183/189 x 183/189	1:2:1	33:80:24	5.35	0.07	189 on 5R, 183 on 5S
GB367	86/89 x 89/89	1:1	60:78	2.35	0.13	86 on 11S
GB372	219/222 x 207/222	1:1:1:1	35:29:31:40	2.10	0.55	207 on 7R, 219 on 7S
GB375	194/197 x 197/197	1:1	69:68	0.01	0.93	194d on 3S
GB377	115/121 x 115/121	1:2:1	52:55:31	12.07	0.00	121 on 8R, 115 on 8S
GB378	182/182 x 179/182	1:1	68:69	0.01	0.93	179 on 4R
GB381	230/233 x 233/233	1:1	60:77	2.11	0.15	230 on 4S
GB387	245/248 x 248/248	1:1	73:59	1.48	0.22	245 on 4S
GB388	236/239 x 236/236	1:1	70:66	0.12	0.73	239 on 10S
GB393	240/240 x 237/240	1:1	66:70	0.12	0.73	240 on 11R
GB394	255/261 x 255/261	1:2:1	34:79:24	4.96	0.08	255 on 1R, 261 on 1S
GB395	373/382 x 370/376	1:1:1:1	35:29:34:39	1.48	0.69	370 on 2R, 373 on 2S
GB802	266/273 x 266/273	1:2:1	32:57:49	8.36	0.02	273 on 7S
GB807	212/215 x 212/215	1:2:1	68:10:60	101.83	0.00	212 on 3R
GB808	371/371 x 362/374	1:1	73:64	0.59	0.44	374 on 4R
GB809	394/397 x 394/394	1:1	64:68	0.12	0.73	397 on 7S
GB812	355/355 x 352/355	1:1	61:74	1.25	0.26	352 on 3R
GB813	371/377 x 371/371	1:1	63:72	0.60	0.44	377 on 4S
GB814	376/385 x 382/385	1:1:1:1	31:29:39:36	1.86	0.60	376 on 8S, 382d on 8R
GB818	129/144 x 129/129	1:1	62:76	1.42	0.23	144 on 1S
GB819	148/148 x 145/151	1:1	71:67	0.12	0.73	151d on 2R
GB822	216/219 x 219/225	1:1:1:1	36:27:36:35	1.70	0.64	225 on 7R, 216 on 7S
GB823	144/160 x 144/148	1:1:1:1	32:39:29:38	2.00	0.57	148d on 1R
GB824	132/135 x 132/135	1:2:1	38:66:32	54.52	0.00	Unlinked
GB826	206/215 x 206/206	1:1	74:64	0.73	0.39	215 on 4S
GB827	214/220 x 214/223	1:1:1:1	32:34:35:30	0.45	0.93	223 on 2R, 220 on 2S
GB828	111/117 x 111/117	1:2:1	29:67:41	2.12	0.35	111 on 9R, 111 on 9S
GB829	141/141 x 141/148	1:1	71:64	0.36	0.55	148 on 2R
GB831	136/139 x 133/145	1:1:1:1	28:33:37:40	2.35	0.50	145 on 1R, 136 on 1S

S2 Table (cont'd). Segregation at new tri-nucleotide simple sequence repeat marker loci in the hazelnut reference mapping population.

Locus	Alleles (Female x Male)	Expected	Observed	χ2	P	LG and alleles
GB832	192/194 x 191/194	1:1:1:1	38:39:26:34	3.06	0.38	191 on 1R, 192 on 1S
GB834	158/158 x 145/158	1:1	59:78	2.64	0.10	145 on 2R
GB835	162/162 x 162/165	1:1	74:64	0.72	0.39	165d on 8R
GB836	196/196 x 193/196	1:1	76:58	2.42	0.12	193d on 10R
GB838	168/174 x 168/174	1:2:1	21:77:40	7.09	0.03	168 on 9R, 174 on 9S
GB841	194/194 x 185/194	1:1	75:63	1.04	0.31	185d on 1R
GB843	185/185 x 176/185	1:1	71:66	0.18	0.67	176d on 2R
GB850	222/225 x 222/225	1:2:1	30:58:49	8.13	0.02	222 on 7R, 225 on 7S
GB851	207/207 x 204/207	1:1	55:78	3.98	0.05	204d on 5R
GB852	305/308 x 308/308	1:1	59:78	2.64	0.10	305 on 7S
GB853	189/189 x 186/189	1:1	60:74	1.46	0.23	186d on 6R
GB855	234/237 x 234/234	1:1	70:62	0.48	0.49	237 on 10S
GB860	293/296 x 293/299	1:1:1:1	32:42:31:33	2.23	0.53	299 on 4R, 296 on 4S
GB867	286/292 x 292/292	1:1	60:76	1.88	0.17	286 on 11S
GB868	287/299 x 287/299	1:2:1	38:65:35	0.59	0.74	287 on 8R, 299 on 8S
GB869	193/193 x 184/193	1:1	46:91	14.78	0.00	184d on 11R
GB870	321/321 x 318/321	1:1	68:70	0.03	0.86	318d on 1R
GB871	335/338 x 338/338	1:1	68:66	0.03	0.86	335 on 6S
GB875	340/343 x 340/340	1:1	77:59	2.38	0.12	343d on 5S
GB876[z]	176/182/187 x 178/182/187/190	--	--	--	--	176 on 4S, 178 on 4R
GB878	282/282 x 282/288	1:1	71:66	0.18	0.67	288d on 2R
GB880	167/167 x 167/173	1:1	58:77	2.67	0.10	173 on 8R
GB887	159/159 x 159/162	1:1	26:110	51.88	0.00	unlinked
GB889	358/367 x 355/367	1:1:1:1	37:36:34:31	0.61	0.89	355d on 2R, 358 on 2S
GB892	259/268 x 259/268	1:2:1	31:78:29	2.91	0.23	268 on 5S, 259 on 5R
GB895	146/146 x 132/146	1:1	66:72	0.26	0.61	132 on 2R
GB903	124/124 x 124/127	1:1	62:72	0.75	0.39	127d on 10R
GB904	376/382 x 376/382	1:2:1	30:62:46	5.13	0.08	382 on 4R, 376 on 4S

S2 Table (cont'd). Segregation at new tri-nucleotide simple sequence repeat marker loci in the hazelnut reference mapping population.

Locus	Alleles (Female x Male)	Expected	Observed	$\chi2$	P	LG and alleles
GB907	187/190 x 187/190	1:2:1	38:67:32	0.55	0.76	190 on 1R
GB910	311/311 x 308/311	1:1	57:80	3.86	0.05	308 on 2R
GB912	166/166 x 157/166	1:1	68:70	0.03	0.86	157 on 2R
GB913	367/382 x 367/367	1:1	74:62	1.06	0.30	382 on 1S
GB915	262/265 x 262/265	1:2:1	31:73:34	0.59	0.74	262 on 10R, 265 on 10S
GB916	248/263 x 242/263	1:1:1:1	42:38:34:23	5.86	0.12	248 on 1S, 242d on 1R
GB917	235/238 x 235/235	1:1	65:71	0.27	0.61	238 on 6S
GB918	305/305 x 299/305	1:1	65:72	0.36	0.55	299d on 6R
GB921	352/355 x 349/355	1:1:1:1	32:36:41:27	3.12	0.37	349 on 5R, 352d on 5S
GB922[y]	195/199 x 195/199/205	1:1	72:64	0.47	0.49	205d on 4R
GB926	252/252 x 249/258	1:1	72:64	0.47	0.49	249d on 1R
GB928	372/372 x 372/375	1:1	63:72	0.60	0.44	375 on 2R
GB930	292/292 x 289/292	1:1	71:64	0.36	0.55	289d on 3R
GB932	375/384 x 375/384	1:2:1	30:84:24	7.04	0.03	384 on 5R, 375 on 5S
GB936	390/390 x 390/393	1:1	65:68	0.07	0.79	393 on 2R
GB937	279/282 x 265/279	1:1:1:1	26:35:35:41	3.35	0.34	265 on 8R, 282d on 8S
GB940	114/120 x 114/120	1:2:1	44:71:23	6.51	0.04	114 on 11R, 120 on 11S
GB941	130/143 x 130/143	1:2:1	32:87:19	11.84	0.00	130 on 8R, 143 accessory on 8S
GB949	148/155 x 148/148	1:1	71:66	0.18	0.67	155d on 10S
GB950	159/162 x 159/162	1:2:1	31:58:49	8.20	0.02	162 on 7S

[z] At locus GB876 there were three alleles in the female parent and four alleles in the male parent. Two alleles were mapped.
[y] At locus GB922 there were two alleles in the female parent and three alleles in the male parent. One allele was mapped.
