# Supplementary material for: In silico development and characterization of tri-nucleotide simple sequence repeat markers in hazelnut (Corylus avellana L.)
Source: PLoS One. 2017 May 22;12(5):e0178061. doi: 10.1371/journal.pone.0178061 (PMC5439716; doi:10.1371/journal.pone.0178061)
Supplement: S3 Table — (PDF) [file pone.0178061.s005.pdf]

S3 Table. Allele sizes at 132 tri-nucleotide repeat microsatellite loci developed from the 'Jefferson' hazelnut genome.

No.	Cultivar	GB301	GB302	GB305	GB306	GB307	GB308	GB309
1	Albania 55	228/228	318/318	220/226	240/240	371/371	284/285	279/279
2	Ala Kieri (COR187)	228/228	318/318	220/220	240/243	362/371	284/285	279/279
3	Alli	218/228	318/318	220/220	240/240	368/371	285/287	279/279
4	Artellet	222/228	318/318	220/220	240/240	371/371	284/284	279/287
5	Aurea	228/228	318/324	220/220	237/240	371/371	285/285	279/279
6	B-3	228/228	315/318	220/220	240/244	368/371	279/290	279/279
7	Barcelloner Zellernuss	228/228	318/318	220/220	243/243	368/368	284/284	279/279
8	Barcelona	222/228	318/318	220/226	240/240	368/371	284/285	279/279
9	Bergeri	228/228	318/318	220/220	237/240	371/371	284/290	279/279
10	Buttner's Zellernuss	228/228	318/318	220/220	240/246	368/368	283/284	279/279
11	Casina	222/228	318/318	220/220	240/240	368/371	284/284	279/279
12	Contorta	218/228	318/318	220/220	240/243	368/368	284/290	279/279
13	Cosford	228/228	318/318	220/220	240/240	368/371	284/285	279/287
14	Cutleaf	228/228	318/318	220/220	237/237	371/371	284/285	277/279
15	Des Anglais	218/228	318/318	220/220	240/243	371/382	279/284	279/279
16	DuChilly	218/228	318/318	220/220	240/243	368/371	285/286	279/279
17	Early Long Zeller	228/228	318/318	220/220	240/240	371/371	287/290	279/279
18	Fusco Rubra	225/228	318/318	217/220	240/240	368/382	285/285	279/279
19	Gasaway	228/228	315/318	220/220	237/240	359/371	284/285	279/279
20	Gem	218/228	318/318	220/226	240/243	368/368	284/286	279/279
21	Gunslebert	228/228	318/318	220/220	237/240	368/368	284/290	279/279
22	Gustav's Zellernuss	228/228	318/318	220/220	237/240	371/371	287/290	279/279
23	Hall's Giant	228/228	318/318	220/220	237/244	368/371	284/290	279/279
24	Iannusa Racinante	217/228	318/318	220/220	240/240	368/368	284/285	279/279
25	Imperiale de Trebizonde	222/228	321/321	220/220	240/243	371/382	284/284	279/279
26	Mortarella	222/228	318/318	220/226	240/240	368/368	284/285	279/279
27	Negret	222/228	318/318	220/220	237/240	368/368	284/285	279/279
28	OSU 026.072	218/228	318/318	220/220	243/243	371/371	284/285	279/279
29	OSU 408.040	218/233	318/324	220/220	240/240	368/371	282/286	279/279
30	OSU 495.049	216/218	315/318	220/220	249/249	368/371	284/285	279/279
31	OSU 054.039	218/218	318/318	220/220	240/244	368/371	286/287	279/279
32	OSU 556.027	218/218	318/324	220/220	240/243	371/371	284/286	279/279
33	OSU 681.078	218/218	315/318	220/220	240/240	359/371	283/283	279/279
34	OSU 759.010	218/228	315/318	220/220	240/240	368/371	285/286	279/279
35	Palaz	222/228	318/324	220/220	240/240	371/382	281/284	279/279
36	Pellicule Rouge	218/218	318/321	220/220	240/243	368/382	279/285	279/279
37	Pendula	228/228	318/330	220/220	237/246	371/371	285/290	287/287
38	Ratoli	222/228	318/318	220/220	240/243	362/368	284/290	279/279
39	Rode Zeller	228/228	318/318	220/220	237/240	368/368	284/284	279/279
40	Römische Nuss	222/228	318/318	220/226	240/240	368/368	284/285	279/279
41	Sant Jaume	228/228	318/318	220/226	240/240	368/371	284/290	279/279
42	Simon	222/222	318/321	220/220	240/240	368/371	284/285	279/287
43	Tapparona di SCC	218/228	318/318	220/220	237/243	368/368	284/284	279/279
44	Tombul Ghiaghli	218/218	318/318	220/220	240/243	368/371	285/286	279/279
45	Tonda Bianca	228/228	315/316	220/220	237/240	368/368	284/285	279/279
46	Tonda di Giffoni	222/228	315/318	220/220	240/240	368/368	284/285	279/279
47	Tonda G. d. Langhe	222/228	318/330	220/220	240/240	368/368	284/290	279/287
48	Tonda Romana	228/228	318/321	220/223	240/243	368/371	284/284	279/279
49	OSU 252.146	222/228	318/318	220/220	240/240	368/371	284/284	279/287
50	OSU 414.062	218/222	318/324	220/220	240/240	368/371	284/285	277/279

S3 Table (cont'd). Allele sizes at 132 tri- nucleotide repeat microsatellite loci developed from the 'Jefferson' hazelnut genome.

No.	GB310	GB313	GB314	GB315	GB317	GB318	GB319	GB322	GB326
1	320/323	318/324	129/129	135/135	130/136	103/106	145/145	117/117	156/156
2	320/329	318/318	126/129	135/135	130/136	112/112	145/148	117/117	150/153
3	320/323	318/324	126/129	135/135	136/139	106/106	145/148	117/120	150/156
4	320/326	321/321	126/126	135/135	136/139	103/106	145/147	117/117	150/153
5	320/320	318/321	129/129	135/135	136/136	103/106	145/145	120/120	150/150
6	320/320	318/321	126/129	129/135	130/136	103/106	145/145	114/117	150/156
7	317/320	318/321	126/129	135/135	136/136	103/103	145/145	117/117	150/156
8	320/320	318/321	126/129	129/135	136/136	103/106	145/148	117/117	150/153
9	317/320	318/318	129/129	135/135	136/139	103/106	145/148	117/117	150/156
10	317/320	318/318	129/129	129/135	127/136	103/106	145/145	117/117	150/150
11	320/320	318/321	126/126	129/135	136/136	103/106	145/148	117/120	150/150
12	320/320	318/318	126/126	135/135	136/139	103/106	145/145	117/117	153/153
13	317/317	318/318	129/129	129/135	130/136	103/103	145/145	117/120	150/150
14	317/320	318/324	129/129	135/135	136/136	103/106	145/145	117/117	150/150
15	320/320	318/318	126/129	129/135	130/139	103/103	145/145	117/120	150/150
16	320/320	318/318	129/129	129/135	136/136	103/112	145/145	117/120	150/150
17	320/323	318/318	126/129	135/135	139/139	103/106	145/148	117/117	150/156
18	320/326	318/318	126/129	135/135	127/136	103/103	145/145	114/117	150/153
19	320/326	318/318	129/129	135/135	137/140	103/112	145/145	114/117	150/156
20	320/320	318/321	126/129	129/135	136/136	106/112	145/148	117/117	150/150
21	320/320	318/321	126/129	135/135	136/139	103/106	145/148	117/117	156/156
22	320/320	318/318	126/129	129/135	139/139	103/106	145/145	117/117	150/156
23	320/320	318/318	126/129	129/135	136/139	106/106	145/145	117/117	150/150
24	320/320	318/321	126/129	129/135	129/136	106/106	145/148	117/117	156/156
25	320/320	318/318	126/126	135/135	136/136	103/103	148/148	117/120	150/156
26	320/320	318/321	126/129	129/135	129/130	103/106	145/148	117/117	150/159
27	320/320	318/321	126/129	129/135	129/136	103/103	148/148	117/117	150/150
28	320/320	321/330	126/129	135/135	136/139	103/112	145/151	117/117	150/150
29	317/320	321/324	126/129	135/135	129/136	103/106	145/145	117/120	156/156
30	320/320	321/324	129/129	135/135	130/137	106/106	145/145	117/120	150/150
31	320/320	318/321	126/129	129/135	136/139	103/103	145/147	117/120	150/150
32	320/320	318/324	129/129	135/135	136/136	112/112	145/147	117/120	150/156
33	320/320	318/318	129/129	135/135	130/139	103/112	145/148	117/117	150/156
34	320/320	321/321	126/129	129/129	136/136	106/106	145/145	111/120	150/150
35	320/320	318/318	126/129	129/129	137/140	103/112	145/148	117/117	150/150
36	320/320	318/318	126/129	129/129	130/136	103/112	145/148	117/120	150/150
37	320/320	318/321	126/129	135/135	136/139	103/103	145/145	117/117	150/156
38	320/326	318/321	126/129	129/135	130/136	103/106	148/148	117/117	150/150
39	317/320	318/318	129/129	129/129	129/136	103/106	145/145	117/117	150/153
40	320/320	318/321	126/129	135/135	129/130	103/103	145/148	117/117	150/156
41	320/320	318/321	126/129	129/135	129/136	106/106	145/148	117/117	153/156
42	320/320	318/321	126/129	129/135	136/139	103/103	145/148	117/117	150/150
43	320/320	318/321	126/129	135/135	129/136	103/103	145/148	117/117	150/150
44	320/320	321/324	126/129	135/135	136/136	103/112	147/148	117/120	150/150
45	320/320	318/324	129/129	135/135	129/136	106/106	145/148	117/120	150/156
46	320/320	321/324	126/129	129/135	129/130	103/106	145/148	117/117	150/150
47	320/320	318/318	126/129	129/135	129/136	103/103	148/148	117/117	150/153
48	320/320	318/321	126/129	129/135	136/136	103/112	145/148	117/117	150/156
49	320/320	318/321	126/129	129/135	136/136	103/106	145/148	117/117	150/150
50	320/326	318/321	126/129	129/135	136/139	103/112	145/148	117/117	150/156

S3 Table (cont'd). Allele sizes at 132 tri- nucleotide repeat microsatellite loci developed from the 'Jefferson' hazelnut genome.

No.	GB327	GB328	GB329	GB332	GB333	GB335	GB338	GB339	GB340
1	316/323	141/141	143/150	275/283	334/352	392/392	380/388	294/303	335/335
2	316/316	141/141	137/150	283/283	343/343	384/392	380/388	303/309	338/338
3	316/316	141/144	143/151	283/286	334/352	392/392	380/388	297/303	335/338
4	316/316	144/144	143/143	283/286	343/352	392/392	380/388	303/303	341/341
5	316/316	141/144	143/150	283/286	352/355	392/392	388/388	291/303	335/335
6	316/316	141/144	140/150	283/286	343/352	392/392	380/380	297/303	335/341
7	307/316	141/144	150/150	283/283	343/352	392/392	380/388	294/300	335/341
8	316/316	141/144	143/150	283/286	343/343	392/392	380/388	303/303	341/341
9	316/316	141/144	140/150	283/283	343/352	392/392	380/388	303/303	335/338
10	307/316	144/144	151/151	283/283	343/343	392/392	380/380	303/303	338/341
11	316/316	144/144	140/150	275/286	343/352	392/392	388/388	303/303	338/341
12	316/316	141/141	140/143	283/286	352/355	392/392	380/388	303/309	341/344
13	307/316	141/144	151/151	283/283	337/343	392/392	380/388	303/303	341/341
14	316/316	141/144	143/151	283/283	343/352	392/392	380/388	300/303	338/341
15	316/316	144/144	151/151	283/286	337/343	392/395	388/388	303/303	338/344
16	316/316	141/144	140/151	283/286	343/352	392/395	388/391	303/303	335/344
17	316/316	141/144	151/151	283/286	334/352	392/392	380/388	297/303	335/338
18	316/316	141/144	143/150	275/286	349/352	392/395	377/380	291/303	338/338
19	316/316	141/144	140/150	275/286	334/343	392/392	380/388	300/309	338/341
20	316/316	141/144	140/143	283/286	343/343	392/392	380/388	303/303	335/341
21	316/316	141/141	143/150	283/283	334/352	392/392	380/388	303/303	338/341
22	316/316	144/144	150/150	275/283	343/352	392/395	380/380	303/303	335/338
23	307/316	141/144	150/150	275/283	343/343	392/395	380/388	303/303	338/338
24	316/316	144/144	143/143	283/286	343/352	392/392	380/388	303/303	341/344
25	316/325	144/144	151/151	286/286	352/352	384/392	388/388	303/306	341/341
26	307/316	144/144	143/150	283/286	343/352	392/395	380/388	303/303	341/341
27	316/316	141/144	150/150	275/286	343/343	392/392	380/388	303/303	338/341
28	316/316	141/144	143/150	283/286	337/337	392/392	388/388	300/306	344/344
29	316/316	141/141	137/150	283/286	343/352	392/392	380/388	303/303	335/335
30	316/316	141/141	143/146	292/292	343/352	384/392	388/397	297/303	338/341
31	316/316	141/144	151/151	286/286	346/352	392/392	380/380	297/300	341/341
32	316/316	141/141	151/151	275/283	337/337	392/392	380/388	303/306	341/341
33	316/316	141/141	137/140	275/275	343/352	392/392	380/388	300/303	338/338
34	316/316	141/144	150/150	286/289	343/352	384/392	388/397	303/306	341/344
35	316/325	144/144	150/150	283/286	352/352	392/392	380/388	303/306	341/341
36	316/316	144/144	140/150	283/286	343/352	392/392	388/388	303/306	341/344
37	307/316	141/144	140/140	275/283	343/352	392/392	380/391	300/303	335/338
38	316/316	144/144	151/151	283/286	343/352	392/392	388/388	303/309	338/341
39	316/316	141/144	143/151	275/283	343/355	392/392	380/388	300/303	335/341
40	316/316	141/144	140/150	283/286	352/352	392/392	388/388	303/303	341/344
41	316/316	144/144	140/143	283/286	343/352	392/392	388/388	303/303	338/341
42	316/316	141/144	143/150	283/286	343/343	392/392	388/388	303/303	341/341
43	316/316	144/144	150/150	283/286	352/352	392/392	380/388	300/303	341/344
44	316/316	141/144	151/151	283/286	337/352	392/392	380/388	297/306	341/341
45	316/316	141/141	143/146	283/283	343/352	392/392	380/380	300/303	347/347
46	316/316	141/144	143/150	283/286	343/343	392/392	380/388	303/303	341/347
47	316/316	141/144	140/146	283/286	343/343	392/392	380/388	300/303	341/344
48	316/316	144/144	140/150	283/286	343/352	392/392	380/388	303/309	338/341
49	316/325	141/144	143/143	283/286	343/343	392/392	380/388	303/303	341/344
50	316/316	144/144	140/151	283/286	343/352	392/392	388/388	303/306	341/344

S3 Table (cont'd). Allele sizes at 132 tri- nucleotide repeat microsatellite loci developed from the 'Jefferson' hazelnut genome.

No.	GB341	GB343	GB344	GB350	GB351	GB352	GB353	GB354	GB356
1	358/361	337/337	160/160	143/155	181/189	376/376	371/371	273/276	361/361
2	361/367	337/337	160/160	155/158	189/190	376/379	371/371	273/276	355/357
3	361/367	337/337	160/160	143/143	181/190	376/379	371/371	276/276	357/357
4	367/367	337/337	160/163	155/158	181/193	376/379	371/371	276/279	361/361
5	367/367	337/337	160/163	143/143	189/189	379/379	371/371	273/276	355/355
6	361/367	337/340	160/160	155/155	181/189	379/379	371/371	276/279	361/361
7	361/367	337/337	160/160	155/155	181/190	379/379	371/374	273/279	355/357
8	361/367	337/340	160/160	155/158	181/190	376/376	371/371	273/279	361/361
9	367/367	337/337	160/160	143/155	181/190	376/379	371/371	276/279	355/357
10	358/361	337/340	160/163	147/155	189/189	376/379	371/371	273/279	355/355
11	361/367	337/340	160/163	155/155	181/189	376/379	371/371	273/279	355/361
12	361/361	337/337	160/160	143/155	181/181	379/379	371/374	276/276	355/355
13	358/361	337/340	160/160	143/155	189/189	376/379	371/371	273/279	355/357
14	361/361	337/337	163/163	143/155	189/190	376/379	371/371	276/276	355/357
15	361/367	337/340	160/160	155/155	181/181	376/379	371/371	276/279	355/361
16	367/367	337/340	160/160	143/155	181/189	379/379	371/371	276/279	355/361
17	361/367	337/337	160/160	143/143	181/189	376/379	371/371	276/276	355/357
18	361/361	337/337	160/163	143/155	190/190	376/379	371/374	279/279	357/361
19	361/367	337/337	160/160	155/155	181/189	379/379	371/371	273/282	355/361
20	361/367	337/340	160/160	143/158	181/189	376/379	371/371	273/279	361/361
21	361/367	337/337	160/160	143/155	181/189	376/379	371/374	276/276	355/361
22	361/367	337/340	160/160	143/143	181/189	376/379	371/371	279/279	355/357
23	367/367	337/340	160/160	143/143	189/189	375/378	371/371	279/279	355/355
24	361/361	337/340	160/160	155/155	181/190	376/379	371/374	273/279	361/361
25	361/367	337/337	160/160	155/155	181/190	376/379	371/374	276/276	355/361
26	367/367	337/340	160/160	155/155	181/181	376/376	371/371	273/279	355/361
27	361/367	337/340	160/163	155/155	181/189	379/379	371/371	279/279	355/357
28	361/367	337/337	160/160	155/155	181/193	376/379	368/374	273/276	357/361
29	361/361	337/337	160/160	143/143	190/190	376/379	371/371	273/276	357/357
30	367/367	337/337	160/160	155/155	181/190	376/379	371/371	279/279	355/357
31	367/367	337/337	160/160	155/155	181/181	379/379	371/371	276/279	355/361
32	361/361	337/337	160/160	155/155	181/190	379/379	371/371	276/276	355/361
33	367/367	337/337	160/163	143/143	181/189	376/379	371/371	276/276	357/357
34	361/361	337/337	160/160	155/155	181/190	379/379	371/371	276/279	357/357
35	361/367	337/337	160/160	155/155	181/181	379/379	371/371	276/279	361/361
36	367/367	337/340	160/160	155/155	181/181	376/379	371/371	279/279	355/355
37	358/367	337/337	160/163	143/155	181/190	379/379	371/371	273/282	357/357
38	361/361	337/340	160/160	155/155	181/189	376/379	371/371	276/276	355/361
39	358/367	340/340	160/160	155/155	189/189	376/379	371/371	273/276	355/361
40	361/361	337/337	160/160	143/155	181/181	376/379	371/371	273/276	355/355
41	361/361	337/340	160/160	155/155	182/190	376/379	371/371	273/279	357/361
42	361/367	337/340	160/160	155/155	181/189	376/379	371/371	279/279	357/361
43	361/367	337/337	160/160	143/155	181/190	376/379	371/371	276/276	361/361
44	361/367	337/337	160/160	155/155	181/181	376/379	371/374	276/279	361/361
45	361/367	337/337	160/160	155/155	181/181	376/376	371/371	273/276	361/361
46	361/367	337/340	160/160	155/155	181/181	376/379	371/371	276/279	361/361
47	361/361	337/340	160/160	143/155	181/193	376/379	371/371	273/279	357/361
48	361/361	337/340	160/160	155/155	181/189	376/379	371/371	273/279	355/361
49	361/367	337/340	160/160	143/158	181/181	379/379	371/371	279/279	361/361
50	361/367	337/340	160/160	155/155	181/190	379/379	371/371	273/279	361/361

S3 Table (cont'd). Allele sizes at 132 tri- nucleotide repeat microsatellite loci developed from the 'Jefferson' hazelnut genome.

No.	GB357	GB358	GB361	GB365	GB367	GB372	GB375	GB376	GB378
1	382/396	364/370	183/183	193/193	86/86	213/213	197/200	349/349	173/185
2	377/380	367/367	183/183	193/193	86/86	216/216	194/197	340/349	182/185
3	382/386	367/370	180/180	193/193	89/89	213/213	197/200	349/349	182/182
4	380/386	367/370	189/189	193/193	86/89	219/222	194/197	340/352	173/182
5	380/397	367/370	186/186	193/193	86/86	213/213	194/197	340/340	179/182
6	380/396	367/370	183/189	193/193	89/89	207/216	194/194	349/349	173/182
7	380/386	370/370	183/189	193/193	86/89	219/222	197/197	340/352	182/185
8	386/393	367/367	183/189	193/193	86/89	207/222	194/194	352/352	182/182
9	377/382	367/367	180/183	193/193	86/89	213/219	194/194	340/340	176/179
10	380/380	367/370	183/189	193/193	86/89	213/222	194/194	340/349	182/182
11	386/397	367/367	183/186	187/193	86/89	213/222	194/197	352/352	176/185
12	382/386	367/367	186/189	193/193	86/86	222/222	194/197	340/352	182/185
13	380/389	367/370	180/183	187/193	89/89	219/222	194/197	340/349	182/182
14	380/382	364/367	183/189	187/193	86/86	213/213	194/200	349/349	179/182
15	386/396	367/367	186/189	193/193	86/89	213/219	194/194	349/352	176/182
16	380/386	367/367	186/186	193/193	86/89	219/222	194/197	340/352	176/182
17	380/386	367/370	180/189	193/193	86/89	213/225	194/197	340/349	182/182
18	382/383	367/370	180/180	193/193	86/86	207/216	197/197	349/352	182/185
19	380/397	370/370	183/189	193/193	86/89	207/207	197/197	340/346	179/179
20	386/393	367/367	189/189	193/193	86/89	222/222	194/197	340/352	176/182
21	377/380	367/370	189/189	193/193	86/89	207/222	194/194	349/352	179/179
22	380/380	367/370	180/189	193/193	86/89	207/225	197/197	340/349	182/182
23	380/386	367/370	183/189	193/193	86/89	207/207	197/197	349/349	182/182
24	380/389	364/367	183/189	193/193	86/89	222/222	194/197	352/352	176/176
25	380/386	367/367	183/189	193/193	86/86	213/216	194/197	340/349	182/182
26	380/386	364/367	189/189	193/193	86/89	219/222	194/194	352/352	182/182
27	380/386	367/370	186/189	193/193	86/89	213/222	194/197	349/352	182/182
28	386/386	364/370	183/183	193/193	89/89	207/213	183/183	340/340	173/185
29	380/397	364/375	183/183	193/193	86/89	207/216	197/197	340/340	182/185
30	386/386	364/364	183/189	193/193	86/86	213/216	183/200	340/349	185/185
31	380/391	367/367	183/189	193/193	86/86	213/216	194/194	349/349	182/182
32	380/389	367/367	189/189	193/196	89/89	216/216	194/197	340/352	182/185
33	377/382	367/367	183/183	193/193	86/89	213/216	194/197	349/349	182/185
34	386/386	364/367	183/189	193/193	86/86	213/222	183/197	352/352	182/182
35	387/389	367/367	183/189	193/193	86/86	213/216	194/197	352/352	182/185
36	386/387	367/367	186/189	193/193	86/89	213/222	194/194	352/352	176/182
37	380/380	367/367	183/189	193/196	86/86	222/222	194/197	340/349	182/185
38	386/386	364/367	186/189	187/193	86/89	213/222	194/194	349/349	182/185
39	380/387	367/367	183/183	187/193	89/89	207/222	194/197	340/352	182/182
40	386/389	367/375	183/186	193/193	86/89	219/222	194/194	346/352	176/182
41	380/386	364/367	183/186	187/193	86/89	219/222	194/194	340/352	182/185
42	386/386	367/370	189/189	193/193	86/89	207/222	194/194	349/352	182/185
43	380/386	367/367	183/189	193/199	86/89	207/222	194/194	349/349	176/185
44	386/389	367/367	183/189	193/193	86/89	216/222	194/197	352/352	176/182
45	380/380	367/370	183/186	193/193	86/86	213/219	194/197	340/352	173/179
46	380/386	367/367	186/189	193/193	86/89	219/222	194/194	352/352	176/179
47	386/389	367/367	183/189	193/193	86/89	213/222	194/197	352/352	182/185
48	386/386	367/367	183/186	193/193	86/89	207/222	194/194	352/352	176/182
49	386/393	367/370	183/189	193/193	86/89	219/222	194/197	340/342	182/182
50	386/389	367/370	183/189	193/193	89/89	207/222	197/197	349/352	179/182

S3 Table (cont'd). Allele sizes at 132 tri- nucleotide repeat microsatellite loci developed from the 'Jefferson' hazelnut genome.

No.	GB381	GB383	GB386	GB387	GB388	GB390	GB393	GB394	GB395
1	233/233	201/201	314/323	248/248	224/236	314/317	237/240	261/261	370/376
2	233/233	198/198	313/313	248/248	236/239	314/314	240/240	261/261	379/388
3	230/233	198/201	313/313	248/248	224/224	314/314	240/240	261/261	388/388
4	233/233	198/198	313/313	248/251	233/236	314/317	240/240	261/261	376/379
5	230/233	198/198	313/313	248/248	236/236	314/314	237/240	255/261	373/388
6	233/233	198/201	311/313	248/248	233/236	314/314	240/240	261/261	370/388
7	230/233	198/201	313/319	248/248	236/236	314/314	237/240	261/261	376/382
8	233/233	201/201	313/318	248/251	236/239	317/318	240/240	255/261	373/376
9	230/230	198/198	313/318	248/248	224/236	314/314	237/237	255/261	373/388
10	230/233	198/198	313/313	245/248	236/236	317/317	237/237	261/261	382/382
11	230/233	201/201	318/318	248/248	224/236	317/318	237/240	255/261	376/379
12	230/233	198/201	312/318	248/248	224/233	314/314	240/240	261/261	373/379
13	230/230	198/201	313/313	245/248	236/236	314/317	237/237	261/261	373/382
14	230/233	198/198	312/314	248/248	236/236	314/314	237/237	261/261	388/388
15	230/230	198/201	311/313	245/248	233/236	314/314	240/240	255/261	373/376
16	230/230	198/198	311/322	245/248	236/242	314/317	240/240	261/261	376/388
17	230/233	198/201	313/313	248/248	224/236	314/317	240/240	261/261	388/388
18	233/233	201/201	313/313	248/248	227/236	314/314	237/237	255/261	388/388
19	233/233	198/201	313/313	248/248	236/236	314/314	237/237	261/261	379/388
20	230/233	198/201	313/322	245/248	239/242	317/318	240/240	261/261	376/388
21	230/233	198/201	312/317	248/251	236/236	317/318	240/240	261/261	376/388
22	230/230	198/201	313/313	245/248	224/236	314/317	237/240	261/261	388/388
23	230/230	198/198	313/313	245/245	224/236	314/317	240/240	261/261	388/388
24	233/233	198/201	313/318	248/248	233/236	314/317	240/240	255/255	376/379
25	230/233	198/201	312/318	245/248	236/236	314/317	237/237	255/261	379/379
26	233/233	198/201	313/318	248/251	233/236	314/314	240/240	255/261	373/376
27	230/233	197/197	313/313	248/248	233/233	314/318	240/240	261/261	376/388
28	230/230	198/201	314/316	248/248	236/236	314/314	240/240	261/261	376/382
29	230/233	201/201	313/313	248/248	233/236	314/314	240/240	261/261	373/379
30	230/242	198/201	312/316	248/248	233/233	314/314	231/237	255/261	364/379
31	230/233	198/198	312/312	245/248	224/236	317/317	240/246	255/261	370/370
32	230/242	198/198	318/318	245/248	236/236	314/314	237/240	255/255	376/379
33	230/233	198/201	313/323	248/248	236/239	314/314	237/240	261/261	382/388
34	224/233	201/201	312/318	245/245	233/236	314/317	237/240	261/261	379/379
35	230/233	198/201	312/312	245/248	236/236	314/314	240/240	261/261	370/376
36	230/233	198/198	312/312	245/248	233/236	314/314	240/240	261/261	376/388
37	233/242	198/201	313/313	245/248	236/239	314/314	237/237	261/261	376/388
38	233/233	198/198	318/318	248/248	233/236	314/318	240/240	255/255	370/370
39	230/233	198/198	313/313	245/248	236/236	314/317	240/240	261/261	382/388
40	233/233	198/201	312/318	248/251	236/236	314/318	237/237	255/261	376/382
41	233/233	198/201	315/318	248/248	236/239	314/317	240/240	255/255	376/388
42	233/233	198/201	313/315	248/251	233/236	314/318	240/240	261/261	376/388
43	233/233	198/201	313/313	248/248	224/236	314/314	231/237	255/255	370/379
44	230/233	198/201	309/317	245/248	236/236	314/314	237/240	255/255	370/376
45	233/233	201/201	313/317	248/251	236/236	314/314	240/240	261/261	373/379
46	233/233	198/201	318/318	248/248	236/236	314/314	240/240	255/261	373/376
47	233/233	198/201	314/318	248/248	233/239	314/314	240/240	255/261	376/388
48	233/233	198/201	312/314	248/251	224/236	314/318	240/240	255/255	376/379
49	230/233	201/201	312/313	245/248	236/239	314/318	240/240	255/261	373/382
50	233/233	201/201	312/318	248/248	236/236	314/317	237/240	255/261	370/376

S3 Table (cont'd). Allele sizes at 132 tri- nucleotide repeat microsatellite loci developed from the 'Jefferson' hazelnut genome.

No.	GB801	GB802	GB808	GB809	GB810	GB812	GB813	GB814	GB817
1	289/294	272/275	374/374	394/397	351/354	355/355	371/373	373/382	359/368
2	291/294	272/275	362/371	394/394	354/357	355/355	371/373	376/382	368/368
3	289/294	266/275	371/374	394/394	354/354	352/355	373/373	382/382	359/368
4	294/294	266/275	371/374	394/394	354/354	352/358	371/371	376/376	368/368
5	289/294	266/275	371/371	394/394	354/357	355/355	373/373	376/382	367/368
6	289/294	272/272	371/374	394/397	345/354	352/355	373/373	376/376	368/368
7	294/294	272/275	371/374	397/397	354/357	352/358	369/371	370/370	368/368
8	294/294	266/271	371/374	394/394	351/354	352/355	373/373	376/382	367/368
9	289/297	271/272	365/371	394/394	345/354	352/355	371/373	376/382	368/368
10	297/297	266/275	371/374	394/394	354/354	352/355	369/371	370/382	368/368
11	289/294	266/275	371/374	394/394	345/354	352/355	371/373	370/376	368/368
12	289/294	272/275	371/371	394/394	354/354	352/355	371/373	382/382	368/369
13	289/297	266/273	371/374	394/397	354/354	355/358	371/377	370/376	362/368
14	289/289	271/275	374/374	394/394	354/354	355/355	369/371	376/376	368/369
15	289/294	266/272	371/371	394/394	345/354	355/355	371/371	376/376	368/368
16	294/294	272/275	371/374	394/394	345/354	355/355	372/372	370/376	368/368
17	289/297	266/272	374/374	394/394	351/354	352/352	373/373	376/382	359/368
18	294/297	272/272	371/374	394/394	351/354	352/355	372/375	376/382	362/368
19	289/294	272/275	371/374	394/394	354/354	355/355	371/373	382/382	367/368
20	294/294	271/275	371/371	394/394	345/351	355/355	373/373	370/376	368/368
21	289/294	271/272	365/374	394/394	354/354	352/355	373/373	376/376	368/368
22	289/297	266/271	374/374	394/394	354/357	352/355	373/373	376/382	359/368
23	289/289	271/272	374/374	394/394	354/357	355/355	371/373	376/382	368/368
24	294/294	272/273	365/374	394/397	345/345	352/355	373/373	373/376	366/368
25	294/297	266/275	371/374	394/397	348/351	346/352	371/373	376/376	368/374
26	294/294	266/266	371/374	394/397	351/354	352/355	373/379	373/376	367/368
27	294/297	266/275	371/374	394/397	351/354	352/355	371/373	370/376	368/368
28	294/294	272/275	371/371	394/394	345/354	352/355	371/371	370/376	368/374
29	294/294	261/272	371/371	394/394	354/354	355/355	371/375	376/376	368/368
30	294/294	269/272	368/371	394/394	345/351	352/352	371/373	385/385	368/368
31	294/294	272/275	362/371	394/394	354/354	352/352	373/373	376/376	374/374
32	289/294	266/273	371/371	394/394	351/354	352/352	371/373	382/385	359/368
33	289/294	272/275	371/371	394/394	354/354	358/358	369/371	382/382	362/368
34	289/294	269/272	371/371	394/394	351/357	352/352	371/371	382/385	368/368
35	294/294	266/275	371/374	394/394	351/354	352/355	372/372	376/376	368/374
36	294/294	266/272	371/374	394/394	345/351	352/355	371/373	376/376	368/368
37	289/294	271/272	371/371	394/394	354/357	355/355	371/373	373/382	368/368
38	289/294	266/275	371/371	394/394	351/354	352/355	371/373	376/382	368/368
39	289/289	272/272	371/371	394/394	351/354	355/358	372/372	376/382	368/368
40	294/294	266/272	371/374	394/394	345/354	352/355	373/373	373/376	367/368
41	294/294	266/275	371/374	394/394	354/354	352/355	371/371	376/376	368/368
42	294/294	271/275	371/371	394/394	351/357	352/352	371/371	376/376	368/368
43	294/294	266/275	371/374	394/397	345/354	352/355	371/371	370/376	366/368
44	294/294	266/266	371/371	394/394	345/351	352/355	372/372	376/385	368/374
45	294/294	272/273	374/374	394/397	354/354	355/355	372/372	373/376	367/368
46	294/294	266/272	371/374	394/397	345/354	352/355	371/373	373/376	367/368
47	294/294	266/275	371/371	394/397	351/357	352/352	371/373	376/382	367/368
48	294/294	266/273	371/371	394/394	345/354	352/355	373/373	376/376	368/368
49	294/294	266/273	371/371	394/397	354/354	355/355	371/377	376/385	367/368
50	294/294	266/273	362/374	394/394	354/354	352/355	371/371	382/385	368/374

S3 Table (cont'd). Allele sizes at 132 tri- nucleotide repeat microsatellite loci developed from the 'Jefferson' hazelnut genome.

No.	GB818	GB819	GB821	GB823	GB826	GB828	GB829	GB831	GB832
1	138/144	148/148	152/152	149/149	206/206	111/117	141/141	142/145	194/194
2	144/144	148/148	158/158	152/160	209/215	111/111	141/141	136/136	189/191
3	138/141	148/148	155/155	152/152	206/218	111/111	141/141	142/145	183/188
4	129/141	148/151	158/158	152/160	206/209	111/117	141/141	136/145	191/195
5	138/138	148/148	152/152	149/149	206/206	111/117	141/141	133/136	191/200
6	129/138	148/148	155/155	154/157	206/209	111/111	141/141	127/142	189/191
7	138/144	148/148	152/155	142/152	206/221	111/117	141/141	127/145	192/192
8	129/144	148/151	155/155	148/160	209/209	111/117	141/141	136/142	191/192
9	138/141	148/148	155/155	142/152	209/218	111/111	141/141	142/145	183/194
10	144/144	148/148	152/155	144/152	206/215	111/111	141/141	136/145	191/192
11	138/138	148/151	155/155	148/160	206/209	111/111	141/141	136/142	186/197
12	129/138	148/148	155/155	144/154	218/218	111/111	141/141	136/145	183/191
13	138/144	148/148	155/155	144/144	206/215	111/111	141/141	142/145	191/195
14	129/138	148/148	158/158	142/152	206/206	111/111	141/141	142/145	188/191
15	138/138	148/148	155/155	144/154	206/215	111/111	141/144	142/145	191/191
16	138/138	148/148	152/155	149/154	209/215	111/111	141/141	142/145	188/191
17	129/138	148/148	155/155	149/152	206/218	111/111	141/141	145/145	183/197
18	129/144	145/148	158/158	152/152	206/206	111/117	141/141	145/145	191/191
19	129/129	148/148	158/158	160/160	206/218	111/117	141/141	133/145	194/197
20	138/144	148/151	155/155	149/160	209/215	111/117	141/141	136/145	188/192
21	129/138	148/151	152/155	144/160	206/218	111/111	141/141	145/145	197/197
22	129/138	148/148	158/158	149/152	206/218	111/111	141/141	145/145	183/197
23	129/144	148/148	152/152	152/152	206/215	111/117	141/141	142/145	191/197
24	138/141	151/151	155/155	148/148	206/206	111/117	141/141	136/142	194/197
25	129/141	145/151	155/170	144/148	206/215	111/111	141/144	142/145	191/203
26	138/141	148/151	155/155	148/160	206/215	111/117	141/141	136/142	188/197
27	129/141	148/151	155/155	142/144	206/206	111/111	141/141	142/145	191/200
28	138/141	145/148	158/170	144/148	206/206	111/111	141/144	136/142	180/194
29	138/141	148/148	155/155	154/154	206/206	111/117	141/141	145/145	183/200
30	129/141	148/148	155/155	155/160	206/215	111/111	141/144	127/136	191/191
31	138/138	145/145	155/155	144/152	206/215	111/111	141/148	139/145	191/197
32	138/138	145/151	158/158	148/148	206/206	111/111	141/144	142/145	191/200
33	138/144	148/148	155/155	149/149	206/206	111/111	141/141	145/145	189/200
34	138/141	148/151	155/155	144/148	203/206	111/111	141/144	136/145	197/200
35	138/138	148/151	155/155	144/144	206/215	111/111	141/144	139/145	191/197
36	129/138	148/148	155/155	144/154	209/215	111/111	141/144	142/145	191/191
37	129/138	148/148	155/155	154/154	206/206	111/117	141/141	136/145	183/183
38	129/129	148/148	155/155	148/148	206/209	111/111	141/141	142/145	191/191
39	138/144	148/148	155/155	144/144	206/215	111/117	141/141	142/145	191/191
40	129/141	148/151	155/155	148/149	206/209	108/111	141/141	136/142	191/194
41	129/129	148/151	155/155	148/152	206/209	111/111	141/141	142/145	191/191
42	129/141	148/151	155/155	142/149	206/209	111/111	141/141	136/145	191/200
43	129/129	151/151	155/155	144/152	206/209	111/111	141/141	136/142	183/191
44	138/138	145/151	154/154	148/149	206/209	111/111	141/144	145/145	191/191
45	141/141	148/148	158/158	148/160	206/209	111/111	141/141	142/142	191/191
46	129/141	148/151	155/155	148/160	209/209	111/111	141/141	142/142	191/191
47	135/138	148/151	155/155	148/149	209/215	111/111	141/141	136/145	188/197
48	129/138	145/151	155/158	144/149	206/209	111/117	141/141	142/145	191/191
49	129/144	148/148	155/155	144/160	206/215	111/117	141/141	136/139	192/194
50	129/129	145/151	155/155	144/148	206/206	111/117	141/148	133/145	191/194

S3 Table (cont'd). Allele sizes at 132 tri- nucleotide repeat microsatellite loci developed from the 'Jefferson' hazelnut genome.

No.	GB834	GB835	GB836	GB838	GB840	GB841	GB843	GB847	GB850
1	158/158	159/165	193/193	174/174	173/173	194/194	173/185	177/179	225/225
2	158/158	162/162	193/196	174/174	173/173	194/194	185/185	177/179	225/225
3	158/158	154/162	196/196	174/174	173/173	194/194	185/185	177/179	222/225
4	158/158	162/162	193/193	174/174	173/173	194/194	176/185	177/179	222/225
5	158/158	162/162	193/196	174/174	173/173	194/194	185/185	179/180	225/225
6	158/158	162/162	196/196	174/174	158/173	185/194	185/185	177/177	225/225
7	158/158	165/165	193/196	174/174	164/173	194/194	185/185	177/179	225/225
8	145/158	162/162	193/196	168/174	173/173	194/194	176/185	177/179	222/225
9	158/158	162/162	193/193	174/174	173/173	185/194	185/185	179/180	225/225
10	158/158	165/165	193/193	174/182	173/173	194/194	185/185	179/179	222/225
11	158/158	162/162	193/196	168/174	173/173	194/194	185/185	177/179	222/225
12	148/158	153/162	193/193	168/182	173/173	194/194	185/185	177/180	219/225
13	158/158	165/165	193/193	174/182	173/173	194/194	185/185	179/179	222/225
14	158/158	162/162	196/196	174/174	173/173	194/194	185/185	179/179	225/225
15	158/158	162/165	193/196	174/174	173/182	194/194	176/176	179/179	222/225
16	158/158	162/165	193/196	168/168	173/173	194/194	185/185	179/179	222/225
17	158/158	162/162	193/193	174/182	173/173	194/194	185/185	179/180	222/225
18	158/158	159/162	193/196	174/182	161/173	185/194	185/185	177/181	213/225
19	158/158	165/168	193/193	174/174	173/173	185/194	185/185	177/180	225/225
20	158/158	162/165	193/196	168/174	173/173	194/194	176/185	179/179	225/225
21	158/158	162/162	193/193	168/174	155/173	194/194	185/185	177/179	225/225
22	145/158	162/162	196/196	174/174	173/173	194/194	185/185	177/180	222/225
23	145/158	162/165	193/196	174/174	173/173	194/194	185/185	177/179	225/225
24	158/158	162/162	193/193	174/174	173/173	194/194	176/185	177/179	225/225
25	158/158	153/162	193/196	174/174	164/173	185/194	176/185	177/179	222/225
26	145/158	162/162	193/196	174/174	173/173	194/194	176/185	177/179	216/222
27	158/158	162/162	193/196	168/174	173/173	194/194	176/185	177/177	222/225
28	148/161	162/165	196/196	174/182	173/173	194/194	185/185	179/180	219/225
29	158/158	154/154	193/196	174/174	173/173	194/194	185/185	177/179	225/225
30	158/158	159/162	193/193	174/174	164/173	194/194	185/185	177/177	225/228
31	158/158	153/162	193/196	174/174	155/173	194/194	185/185	177/179	225/225
32	158/161	162/162	193/193	168/174	173/173	185/194	173/176	179/179	216/222
33	158/158	162/171	193/196	174/174	173/173	194/194	185/185	177/177	225/225
34	158/158	159/162	196/196	174/174	164/173	194/194	185/185	177/180	225/225
35	158/158	153/153	193/193	168/174	164/173	185/194	176/185	177/177	222/225
36	158/158	153/162	193/193	168/174	164/173	194/194	176/185	179/179	222/225
37	158/158	165/165	196/196	174/174	173/173	194/194	185/185	177/177	225/225
38	158/158	162/162	193/193	168/174	173/173	185/194	185/185	177/179	222/225
39	158/158	165/165	193/193	174/182	173/173	194/194	185/185	179/180	216/225
40	158/158	162/162	193/196	168/174	173/176	194/194	185/185	177/179	222/231
41	158/158	162/162	193/196	174/174	173/173	185/194	176/185	179/179	222/225
42	145/158	162/162	193/193	168/168	173/173	194/194	185/185	177/179	219/225
43	158/158	162/162	193/196	174/174	173/173	185/194	176/185	177/179	222/225
44	158/158	162/162	193/196	168/174	164/173	194/194	176/176	177/179	222/222
45	145/158	162/162	193/193	168/174	155/173	185/194	185/185	179/179	225/225
46	145/158	162/162	193/193	168/168	155/173	185/194	176/185	177/179	222/225
47	158/158	162/162	193/196	174/174	173/173	185/194	176/185	177/179	222/225
48	158/158	162/165	193/193	168/174	173/173	194/194	176/176	177/177	222/225
49	158/158	162/162	196/196	168/174	173/173	194/194	185/185	179/179	222/225
50	145/158	162/165	196/196	168/174	173/173	185/194	176/185	179/179	222/225

S3 Table (cont'd). Allele sizes at 132 tri- nucleotide repeat microsatellite loci developed from the 'Jefferson' hazelnut genome.

No.	GB851	GB852	GB853	GB854	GB855	GB856	GB858	GB860	GB865
1	201/204	305/305	188/188	206/206	234/234	256/256	327/327	293/296	259/262
2	207/210	296/305	182/188	206/218	234/234	253/253	330/330	293/299	262/262
3	201/207	296/314	188/188	206/206	234/234	253/256	327/327	293/299	262/262
4	204/207	305/305	188/188	206/206	234/234	258/258	327/327	293/293	262/262
5	204/207	296/296	188/188	206/206	234/237	253/256	327/327	293/293	259/262
6	198/207	305/305	188/188	206/206	234/234	256/256	327/327	293/293	259/262
7	207/207	296/305	179/188	206/206	234/237	253/258	330/330	293/293	262/262
8	204/207	296/308	185/188	206/206	234/237	258/258	327/327	293/293	262/262
9	207/207	296/305	188/188	206/206	234/234	258/258	330/333	293/299	268/268
10	204/204	296/305	188/188	206/206	234/234	258/258	330/330	293/293	262/262
11	207/207	296/308	188/188	206/206	234/234	258/258	327/327	293/299	262/262
12	207/207	305/305	185/185	206/206	234/237	258/258	327/327	293/299	262/262
13	198/204	296/305	188/188	206/206	234/234	258/258	327/327	293/296	262/262
14	201/201	305/308	188/188	206/206	234/234	253/256	327/327	293/299	262/265
15	198/207	299/305	188/188	206/218	234/234	258/258	327/327	293/293	262/262
16	204/207	305/305	182/188	206/206	234/234	258/258	330/330	293/293	262/262
17	204/204	296/314	188/188	206/206	234/234	256/258	327/327	293/299	262/265
18	204/210	296/305	185/188	206/206	234/237	253/258	330/330	293/293	259/262
19	207/210	305/305	188/188	206/206	234/234	258/258	327/327	293/299	262/262
20	204/207	305/308	182/188	206/206	234/237	258/258	327/327	293/293	262/262
21	204/207	299/314	188/188	206/206	234/234	258/258	327/327	293/299	265/268
22	204/204	296/305	185/188	206/206	234/234	256/258	327/327	296/299	265/268
23	204/204	305/305	185/188	206/206	234/234	256/258	330/330	293/296	262/268
24	204/207	305/308	185/188	206/206	234/234	258/258	327/327	293/293	259/262
25	207/207	305/305	188/188	203/206	234/234	253/258	330/330	293/293	262/262
26	204/207	296/308	188/188	206/206	234/234	258/258	327/327	293/293	262/262
27	204/207	296/308	188/188	206/206	233/233	254/254	327/327	293/293	262/262
28	198/207	305/305	185/188	206/206	234/234	256/258	327/330	293/299	262/265
29	198/198	305/305	179/188	206/206	234/234	258/258	327/327	293/293	262/262
30	207/226	305/308	188/188	206/206	234/234	253/256	330/330	296/299	262/262
31	198/207	299/305	188/188	206/206	234/234	258/258	330/330	293/293	262/262
32	207/207	305/305	185/188	206/212	234/234	257/265	330/330	293/293	262/262
33	207/207	296/296	182/188	206/206	234/234	258/258	327/327	293/296	262/262
34	207/207	305/308	185/188	203/206	234/234	258/258	330/330	293/293	262/262
35	207/207	305/308	188/188	203/206	234/240	258/258	327/327	293/293	262/262
36	207/207	305/314	188/188	206/206	234/234	258/258	327/327	293/293	262/262
37	198/198	308/308	188/188	206/218	234/234	253/256	330/330	296/299	262/265
38	198/207	296/308	188/188	206/206	234/234	258/258	327/327	293/293	259/262
39	204/207	305/305	188/188	206/206	234/234	258/258	330/330	293/293	262/262
40	207/207	305/308	188/188	206/206	234/234	258/258	330/330	293/293	262/262
41	198/204	296/308	188/188	206/206	234/237	255/258	330/330	293/293	259/262
42	204/207	305/308	188/188	206/206	234/234	253/253	330/330	293/293	265/265
43	207/207	296/308	188/188	206/206	234/234	258/258	327/327	293/293	259/262
44	207/207	305/308	188/188	206/206	234/234	253/258	330/330	293/293	262/262
45	207/207	296/299	188/188	206/212	234/234	253/258	330/330	293/296	262/265
46	207/207	296/308	188/188	206/206	234/234	258/258	330/330	293/296	262/265
47	204/207	305/308	188/188	206/218	234/234	256/256	327/327	293/293	259/262
48	207/207	305/308	188/188	206/206	234/234	258/258	327/327	293/293	259/262
49	207/207	305/308	189/189	206/206	234/237	258/258	330/330	293/296	262/262
50	204/207	308/308	186/189	206/206	234/234	258/258	327/327	293/299	262/262

S3 Table (cont'd). Allele sizes at 132 tri- nucleotide repeat microsatellite loci developed from the 'Jefferson' hazelnut genome.

No.	GB866	GB867	GB868	GB869	GB870	GB871	GB873	GB875	GB878
1	248/248	292/292	296/299	193/193	318/321	335/335	274/279	331/340	282/282
2	249/252	292/292	287/289	193/193	318/321	338/338	274/274	340/343	282/282
3	250/250	292/292	287/296	193/193	318/321	335/335	274/274	331/343	282/294
4	248/252	292/301	287/289	193/193	318/321	335/338	268/274	340/349	282/288
5	248/252	292/292	296/299	184/193	321/321	335/338	274/279	334/343	282/294
6	250/250	292/292	296/299	193/196	321/321	335/338	274/279	340/343	282/294
7	248/248	292/292	296/296	193/193	321/321	335/335	279/279	340/343	282/294
8	248/252	286/292	287/299	193/193	321/321	335/338	274/274	340/340	282/288
9	248/252	292/292	296/299	193/193	321/321	335/335	274/274	343/349	282/294
10	248/252	286/292	296/299	193/193	321/321	335/338	271/274	343/343	282/282
11	248/252	292/292	287/299	193/193	318/321	335/338	274/279	340/343	282/288
12	248/248	286/292	287/299	193/193	321/321	335/335	274/274	334/340	282/294
13	246/250	292/292	296/296	193/193	321/321	335/335	271/279	334/343	282/282
14	248/252	292/292	290/290	193/193	318/324	332/335	268/279	331/343	282/282
15	248/252	292/292	296/296	193/196	318/321	335/338	268/274	334/343	273/294
16	250/250	286/292	287/299	193/193	318/324	335/335	274/274	343/343	282/282
17	250/250	286/292	287/299	193/193	321/321	335/335	274/279	343/343	282/294
18	250/250	286/292	287/296	193/193	318/318	335/335	268/274	334/343	282/282
19	250/250	292/301	299/299	184/193	318/324	338/338	274/279	343/349	282/282
20	250/250	286/292	287/299	193/193	318/321	335/335	274/274	340/343	282/288
21	248/248	292/298	287/299	184/193	318/321	335/335	274/274	340/343	282/294
22	248/252	286/292	287/299	193/193	321/321	335/335	274/274	343/343	282/294
23	248/248	286/292	289/299	193/193	318/321	335/335	271/274	343/349	282/282
24	248/252	292/292	287/299	193/193	318/321	332/335	274/274	340/340	282/288
25	250/250	292/301	287/287	193/193	318/321	335/338	274/274	328/340	282/288
26	248/252	292/292	287/299	193/193	318/321	335/335	274/274	340/343	282/294
27	248/252	286/292	287/296	184/193	321/321	335/335	274/279	340/340	282/288
28	250/250	292/292	287/289	193/193	318/324	335/341	274/274	340/340	282/288
29	248/252	286/298	287/296	193/193	321/321	335/338	268/279	334/334	282/282
30	249/252	292/292	287/299	193/193	318/318	335/335	274/274	340/343	282/282
31	250/250	292/292	287/287	193/193	318/318	335/335	274/274	340/343	282/282
32	250/250	292/292	287/296	193/193	318/318	338/338	274/274	343/343	282/282
33	250/250	292/292	287/299	193/193	321/321	335/338	271/274	334/340	282/282
34	250/250	292/295	287/289	193/193	318/318	329/335	274/274	340/343	282/288
35	250/250	292/301	287/287	193/193	318/318	335/338	274/274	340/343	282/288
36	250/250	292/292	287/296	193/193	318/321	335/335	274/274	340/343	282/294
37	248/248	292/292	287/287	193/193	321/321	335/335	274/279	325/343	273/282
38	250/250	298/301	287/287	193/193	321/321	335/341	268/274	334/340	288/294
39	248/252	292/292	296/296	193/193	318/321	335/335	268/279	343/343	282/282
40	248/252	292/301	287/299	193/193	318/321	335/335	274/274	340/343	282/288
41	252/252	292/292	287/299	193/193	321/321	335/338	268/274	325/340	288/294
42	248/248	292/292	287/299	193/193	321/321	335/335	274/279	340/340	282/288
43	248/252	292/301	287/299	193/193	318/318	335/335	274/274	340/343	282/294
44	250/250	292/292	287/296	193/193	318/318	335/338	274/274	340/343	282/288
45	246/250	298/298	287/299	184/193	321/321	335/338	274/279	340/343	279/282
46	248/252	292/298	287/299	184/193	321/321	335/335	274/274	340/340	282/288
47	248/252	292/292	287/287	193/193	318/324	335/338	274/274	337/340	288/297
48	250/250	292/292	287/299	193/193	321/321	335/335	274/274	340/349	282/288
49	248/252	286/292	287/299	193/193	321/321	335/338	274/274	340/343	282/282
50	250/250	292/292	287/299	184/193	318/321	338/338	274/274	340/340	282/288

S3 Table (cont'd). Allele sizes at 132 tri- nucleotide repeat microsatellite loci developed from the 'Jefferson' hazelnut genome.

No.	GB880	GB887	GB889	GB891	GB892	GB893	GB894	GB895	GB896
1	167/167	162/162	373/376	281/281	259/259	296/296	302/302	146/149	394/394
2	167/173	159/165	367/373	281/281	259/262	296/299	302/308	146/146	400/412
3	167/167	159/159	355/367	275/281	259/274	296/299	308/308	146/146	394/400
4	167/173	159/162	355/376	281/281	259/262	296/299	302/305	146/146	394/412
5	167/167	162/168	355/367	275/281	259/274	296/299	302/302	146/149	394/412
6	167/173	159/162	367/367	275/281	259/259	299/299	305/308	146/146	391/400
7	167/167	159/168	355/355	275/275	259/259	296/296	308/308	146/146	391/400
8	167/167	159/159	355/358	281/281	259/268	299/299	302/305	146/146	394/400
9	167/167	159/162	367/367	281/281	259/259	296/296	302/302	146/146	394/400
10	167/173	162/165	355/367	275/275	259/259	296/299	308/308	146/146	400/412
11	167/167	159/162	367/367	281/281	259/259	296/299	305/305	146/146	400/400
12	167/167	159/159	355/367	275/278	259/259	296/299	308/308	146/146	400/400
13	167/167	159/165	367/367	275/275	259/259	296/296	305/305	146/146	400/412
14	167/167	162/168	367/367	275/281	259/259	296/299	302/308	146/146	400/400
15	167/167	159/162	355/355	275/281	259/259	296/299	302/305	146/149	394/400
16	167/173	159/162	367/367	281/281	259/259	296/299	305/308	146/146	400/400
17	167/167	159/159	367/367	278/281	259/259	296/296	302/302	146/146	394/412
18	167/173	162/162	361/376	278/281	259/259	296/299	302/308	146/146	400/412
19	167/167	159/159	367/367	275/281	259/259	296/296	302/308	146/146	394/397
20	167/173	159/159	355/367	281/281	259/259	296/298	302/308	146/146	400/400
21	167/167	162/162	358/367	281/281	259/274	296/296	308/308	146/146	394/394
22	167/167	159/159	355/355	278/281	259/259	296/299	302/305	146/149	394/412
23	167/167	159/159	355/367	278/278	259/259	296/299	305/305	146/149	394/412
24	167/167	159/159	355/358	278/281	268/277	296/296	305/305	146/146	400/400
25	167/167	159/165	355/367	281/281	259/259	296/296	305/305	132/146	400/400
26	167/167	159/159	355/358	281/281	259/259	298/299	305/308	146/146	394/412
27	167/173	159/162	355/370	275/275	259/259	296/298	308/308	146/146	394/394
28	167/167	159/159	376/376	281/281	259/259	296/296	308/308	146/146	400/412
29	167/173	156/159	367/367	275/281	259/259	298/299	302/308	146/149	400/400
30	167/167	159/165	367/367	275/275	259/259	296/296	305/305	146/146	394/403
31	167/173	159/165	367/367	275/275	259/259	296/296	305/305	132/146	394/394
32	167/167	159/159	355/373	275/275	259/259	298/298	302/305	132/149	394/394
33	167/167	159/159	355/355	281/281	259/259	296/299	302/305	146/146	400/412
34	167/167	159/165	367/367	281/281	259/259	296/298	302/302	146/146	394/400
35	167/167	162/168	355/367	275/281	259/259	296/298	305/305	132/146	394/394
36	167/167	159/168	355/367	275/281	259/259	296/296	305/305	146/146	400/400
37	167/167	159/162	367/367	281/281	259/259	296/296	305/308	146/149	400/400
38	167/167	159/159	367/376	275/281	259/259	298/305	302/305	146/146	394/400
39	167/167	159/165	358/367	275/281	259/259	296/299	305/305	146/146	400/400
40	167/167	159/162	355/358	275/275	259/268	296/299	305/308	146/146	400/400
41	167/167	159/162	355/367	275/281	259/268	299/305	302/305	146/146	394/400
42	167/173	159/159	355/370	275/281	259/259	298/299	308/308	146/146	394/400
43	167/167	159/162	355/367	281/281	259/259	296/299	305/308	146/149	400/400
44	167/167	159/168	355/367	275/281	259/259	296/298	305/308	132/146	394/400
45	167/167	162/162	358/367	281/281	268/274	296/299	302/308	146/146	394/400
46	167/173	159/162	355/358	275/281	259/274	296/299	305/308	146/146	400/400
47	167/167	159/162	355/364	281/281	259/259	298/299	305/305	146/146	394/400
48	167/167	159/162	355/367	275/281	256/259	296/296	305/305	146/146	400/400
49	167/167	159/159	358/367	281/281	259/268	296/299	302/302	146/146	394/394
50	167/173	159/162	355/367	281/281	259/268	299/299	305/305	132/146	394/394

S3 Table (cont'd). Allele sizes at 132 tri- nucleotide repeat microsatellite loci developed from the 'Jefferson' hazelnut genome.

No.	GB902	GB904	GB906	GB907	GB908	GB909	GB912	GB913	GB916
1	117/117	376/376	122/125	184/187	356/356	345/345	157/157	375/381	242/257
2	108/111	376/382	122/125	178/187	355/355	348/348	157/157	381/381	248/263
3	117/117	382/382	122/122	187/187	356/356	345/348	157/160	375/378	258/263
4	117/117	376/382	119/125	187/187	356/356	345/345	160/166	366/378	249/249
5	117/117	376/377	122/125	187/190	356/356	345/348	151/151	375/375	248/257
6	111/111	372/372	122/122	187/190	356/358	345/345	157/157	375/375	249/263
7	117/117	376/382	122/125	184/187	356/358	348/348	166/166	375/381	248/248
8	117/117	372/382	119/122	187/187	356/358	348/348	166/166	381/381	249/263
9	111/111	372/382	122/125	187/187	356/358	345/348	157/166	375/378	250/260
10	111/123	372/376	125/125	184/190	356/356	345/348	157/166	381/381	249/263
11	114/114	372/372	122/122	178/187	356/358	345/348	166/166	375/375	248/257
12	117/117	382/382	122/125	187/190	356/358	345/348	166/166	375/375	248/263
13	111/111	376/376	125/125	178/190	356/358	345/348	166/166	375/381	250/257
14	117/117	376/376	125/125	178/187	358/358	345/348	157/166	366/375	248/257
15	111/111	376/382	119/122	187/190	356/356	345/345	166/166	375/375	263/263
16	117/117	372/376	122/125	187/190	356/358	345/348	157/166	375/375	248/248
17	111/123	376/382	122/125	184/187	356/356	345/348	151/157	366/375	260/263
18	114/117	382/382	125/125	184/187	356/356	348/348	157/166	366/381	248/269
19	117/117	372/382	125/125	187/187	356/356	345/348	161/161	366/366	242/248
20	117/117	372/376	122/122	187/187	356/358	348/348	157/166	375/381	249/249
21	117/117	376/382	119/125	184/191	356/356	345/348	157/166	366/375	249/260
22	111/123	376/382	119/122	184/187	356/358	348/348	151/157	366/375	260/263
23	111/111	376/376	119/122	187/187	356/358	345/348	157/166	366/381	260/269
24	117/117	376/376	122/122	178/187	356/356	345/348	151/151	375/378	249/249
25	111/117	376/376	119/125	187/190	356/356	345/348	157/166	366/378	242/260
26	117/117	382/382	119/125	187/187	356/356	345/348	157/157	375/378	248/266
27	123/123	376/382	119/119	187/190	356/356	345/345	166/166	378/378	249/263
28	111/111	376/376	125/125	187/187	356/358	348/348	157/166	375/378	248/260
29	117/117	376/376	122/125	187/187	356/358	348/348	157/166	375/378	248/263
30	111/111	372/373	125/125	184/187	356/356	345/348	157/157	366/378	260/263
31	111/111	376/376	119/125	184/190	356/356	345/348	157/157	375/375	249/261
32	111/117	382/382	119/125	187/187	356/356	348/348	166/166	375/375	249/263
33	117/117	376/382	125/125	184/187	356/358	348/348	166/166	375/381	263/268
34	117/117	372/382	119/125	184/190	356/356	345/348	157/157	375/378	249/260
35	111/117	376/382	119/125	190/190	356/356	345/348	157/166	375/375	248/248
36	111/111	372/376	119/122	190/190	356/358	345/345	166/166	375/375	248/263
37	117/117	376/382	125/125	178/187	356/358	345/348	157/166	366/375	248/269
38	111/111	372/382	119/122	187/187	356/358	348/348	166/166	366/366	246/261
39	111/117	376/382	125/125	178/191	356/356	345/348	166/166	375/381	250/258
40	117/117	372/376	122/125	187/187	356/358	345/348	151/166	378/378	249/263
41	117/117	382/382	113/119	184/187	356/358	348/348	166/166	366/366	248/263
42	111/123	376/382	119/122	187/187	356/358	345/348	166/166	378/378	248/248
43	111/111	372/376	122/125	187/190	358/358	348/348	157/166	366/381	249/263
44	111/111	372/382	119/122	187/187	356/358	348/348	157/166	375/375	248/260
45	117/117	376/376	119/122	178/184	356/356	345/345	151/151	378/378	263/266
46	111/117	372/376	122/122	184/187	356/358	345/348	166/166	378/378	263/263
47	111/117	372/376	119/125	178/187	358/358	345/348	166/166	373/375	249/266
48	111/111	372/376	122/122	187/190	356/358	345/348	157/166	375/375	248/263
49	117/117	376/382	119/119	187/190	356/356	348/348	166/166	367/382	248/263
50	117/117	376/382	125/125	187/190	356/356	348/348	157/166	367/367	242/263

S3 Table (cont'd). Allele sizes at 132 tri- nucleotide repeat microsatellite loci developed from the 'Jefferson' hazelnut genome.

No.	GB917	GB921	GB924	GB925	GB928	GB930	GB931	GB932	GB936
1	235/235	355/355	218/218	378/378	372/375	290/292	361/361	396/396	390/390
2	235/235	355/360	218/218	378/378	372/372	292/301	362/364	396/396	390/390
3	235/238	355/355	218/218	372/381	372/372	292/292	361/372	381/381	390/390
4	235/238	343/355	218/218	378/381	372/372	292/292	361/361	384/384	390/393
5	235/235	360/360	218/221	378/378	372/372	292/292	361/361	396/396	390/390
6	235/238	349/355	221/221	378/381	372/375	292/292	372/372	384/384	390/396
7	235/235	355/358	218/221	378/378	372/372	292/292	362/364	390/390	390/396
8	235/238	349/355	218/221	372/372	372/375	292/292	361/372	375/384	390/393
9	235/235	355/355	218/218	372/378	372/372	292/292	361/372	381/396	390/396
10	235/235	343/355	218/218	378/378	372/372	292/292	361/369	390/396	396/399
11	235/238	355/355	218/221	378/381	372/372	292/301	362/372	384/387	390/390
12	235/238	352/355	218/221	372/378	372/372	290/292	361/361	387/390	396/396
13	235/235	343/355	218/221	378/378	372/372	292/292	364/369	381/396	390/396
14	235/235	352/355	218/221	378/378	372/372	292/301	372/372	396/396	390/390
15	235/238	352/355	218/221	372/381	372/372	292/292	362/369	384/387	390/396
16	238/238	355/355	218/221	372/381	372/372	292/292	369/372	387/396	390/396
17	235/238	355/355	218/218	372/378	372/372	292/292	361/370	381/390	390/390
18	235/235	355/355	218/218	378/381	372/372	289/289	361/373	381/396	390/390
19	235/235	343/355	218/218	381/381	372/372	289/292	361/362	387/396	393/393
20	238/238	349/355	218/221	372/372	372/372	292/292	369/372	384/396	390/396
21	235/238	352/355	218/221	378/381	372/372	292/292	361/373	384/390	390/399
22	235/238	355/355	218/218	372/372	372/372	292/292	361/362	381/390	390/390
23	235/238	352/355	218/218	372/372	372/372	292/292	362/369	390/396	390/399
24	235/238	343/355	218/221	372/378	372/372	292/292	361/373	375/384	390/390
25	235/238	349/355	221/221	372/378	372/375	292/292	361/369	384/384	390/390
26	238/238	343/349	218/221	372/378	372/375	292/292	372/372	384/396	390/393
27	238/238	343/355	218/221	372/372	372/372	292/292	364/372	387/396	390/390
28	238/238	355/355	218/218	378/381	372/372	292/292	361/364	381/396	390/396
29	235/235	358/360	218/218	378/378	372/372	292/292	361/361	396/396	390/390
30	235/238	343/355	218/221	381/381	372/372	292/292	361/361	387/399	390/396
31	238/238	340/349	218/221	381/381	372/372	292/292	361/369	384/396	390/399
32	235/238	352/352	218/221	378/378	372/372	292/292	372/372	384/396	390/399
33	235/235	343/352	218/218	378/378	372/372	292/292	364/364	384/384	390/390
34	238/238	352/355	218/218	372/378	372/372	292/292	361/372	387/399	390/396
35	238/238	349/352	218/221	372/381	372/372	292/292	369/372	384/387	390/390
36	238/238	352/355	218/221	372/381	372/372	292/292	369/372	387/390	390/396
37	235/235	355/355	218/218	378/378	372/372	292/292	361/361	384/396	390/390
38	235/238	349/355	221/221	372/381	372/372	289/292	372/372	384/387	390/390
39	235/235	343/355	221/221	378/381	372/372	292/292	369/373	396/396	390/396
40	235/238	355/355	218/221	378/381	372/375	292/292	372/373	387/396	390/393
41	235/235	352/355	218/218	372/378	372/372	292/292	361/361	375/396	390/390
42	238/238	343/349	218/218	372/372	372/375	292/292	372/373	384/396	390/393
43	235/238	349/355	218/221	378/381	372/375	292/292	361/372	384/384	390/390
44	235/238	352/352	218/221	372/372	372/372	292/292	372/372	384/387	390/399
45	235/235	355/355	218/221	372/378	372/375	292/292	373/373	375/381	390/393
46	235/238	349/355	221/221	378/381	372/375	292/292	372/373	381/384	390/393
47	235/238	349/355	221/221	378/381	372/372	292/292	364/372	384/396	390/390
48	235/238	355/355	218/221	378/381	372/372	292/292	370/372	387/396	390/390
49	235/238	352/355	221/221	372/372	372/372	292/292	369/372	375/384	390/390
50	235/235	349/355	218/218	381/381	372/375	289/292	361/362	375/384	390/393

S3 Table (cont'd). Allele sizes at 132 tri- nucleotide repeat microsatellite loci developed from the 'Jefferson' hazelnut genome.

No.	GB937	GB940	GB941	GB942	GB944	GB946	GB949	GB950
1	273/273	114/120	133/133	361/361	286/286	341/344	155/155	156/159
2	265/279	114/120	143/143	355/361	286/286	341/341	158/158	156/156
3	273/273	120/120	143/143	361/364	286/286	341/341	155/155	159/159
4	265/282	114/114	143/143	355/355	286/286	341/341	148/155	159/159
5	273/279	120/120	150/153	361/361	286/286	341/347	155/158	156/162
6	265/273	114/120	140/143	361/361	286/290	341/344	148/155	162/165
7	273/278	114/120	133/150	361/364	286/286	341/341	148/158	156/159
8	279/282	120/120	143/143	355/361	286/286	341/344	148/155	159/165
9	273/273	114/114	140/143	361/361	286/286	341/341	148/148	156/165
10	265/273	120/120	134/140	361/361	286/286	341/344	155/158	159/159
11	279/282	114/114	143/143	355/361	286/286	341/344	155/155	159/162
12	273/273	114/120	143/143	355/364	286/286	341/344	148/155	162/165
13	273/279	120/120	133/143	361/361	286/286	341/344	155/155	156/159
14	273/278	114/114	143/143	355/361	286/286	341/341	155/155	162/165
15	273/273	114/120	140/143	361/361	286/286	341/341	155/155	159/159
16	265/273	114/120	130/140	361/361	286/286	340/340	148/155	159/162
17	273/273	120/120	143/143	361/361	286/286	341/341	148/148	156/159
18	265/273	114/120	133/133	361/361	286/286	341/344	158/158	159/159
19	273/279	114/120	143/143	355/361	286/286	341/344	155/155	156/159
20	265/282	120/120	140/143	361/361	286/286	341/344	148/155	159/165
21	273/282	114/114	143/143	361/361	286/286	341/344	148/148	162/165
22	273/273	120/120	143/143	361/361	286/286	341/341	155/155	159/165
23	273/279	120/120	143/143	361/361	286/286	341/344	148/155	165/165
24	279/279	114/120	133/143	361/361	286/286	341/341	148/155	159/162
25	273/282	114/114	143/153	361/361	286/286	341/341	155/161	159/159
26	273/282	120/120	143/143	355/361	286/286	341/341	148/155	159/165
27	265/273	114/120	143/147	355/361	286/286	341/344	155/155	159/162
28	273/275	114/114	133/143	355/361	286/286	341/341	148/155	159/165
29	265/273	114/120	143/143	355/361	286/286	341/341	155/155	159/171
30	265/279	114/114	143/143	355/361	286/290	341/341	155/155	156/162
31	275/275	114/114	130/147	361/361	286/286	341/341	155/161	159/162
32	273/276	114/114	130/130	361/361	286/286	341/341	155/155	159/159
33	279/279	114/120	133/140	361/361	286/286	341/341	148/158	159/165
34	273/273	114/114	130/143	361/361	286/286	341/341	155/161	162/165
35	273/282	120/120	143/147	361/361	286/286	341/341	155/161	159/159
36	273/273	120/120	130/140	361/361	286/286	341/341	148/155	159/162
37	273/282	114/120	143/143	361/361	286/286	341/341	155/158	156/156
38	278/282	120/120	143/143	361/361	286/286	341/341	155/155	159/159
39	273/279	120/120	133/140	361/361	286/286	341/344	155/155	157/157
40	279/282	114/120	133/147	361/361	286/286	341/341	155/155	156/159
41	279/279	114/120	143/143	361/361	286/286	341/344	148/155	159/159
42	265/282	120/120	143/147	355/361	286/286	341/341	155/155	162/165
43	273/282	114/120	143/147	355/361	286/286	341/341	148/158	156/159
44	273/282	114/120	130/130	361/361	286/286	341/341	155/158	159/159
45	265/279	114/114	133/143	355/361	286/286	341/341	148/155	162/162
46	265/279	114/114	133/147	355/361	286/286	341/341	155/155	159/162
47	276/282	114/120	130/143	361/361	286/286	341/341	155/155	159/159
48	273/282	120/120	130/143	361/361	286/286	341/344	155/158	159/165
49	279/282	114/120	130/143	361/361	286/286	347/347	148/155	159/162
50	265/279	114/120	130/143	361/361	286/286	341/341	148/148	159/162
