# Supplementary material for: In silico development and characterization of tri-nucleotide simple sequence repeat markers in hazelnut (Corylus avellana L.)
Source: PLoS One. 2017 May 22;12(5):e0178061. doi: 10.1371/journal.pone.0178061 (PMC5439716; doi:10.1371/journal.pone.0178061)
Supplement: S4 Table — Three or four amplicons were observed in some accessions. (PDF) [file pone.0178061.s006.pdf]

S4 Table. Amplicon sizes at 18 tri-nucleotide repeat microsatellite loci developed from the 'Jefferson' hazelnut genome. Three or four amplicons were observed in some accessions.

No.	Cultivar	GB311	GB346	GB377	GB807	GB815
1	Albania 55	282/282	361/372	121/121	215/215	236/236
2	Ala Kieri (COR187)	276/282	361/372	121/121	215/215	239/245
3	Alli	273/276	372/372	118/121/109	203/215	239/245
4	Artellet	273/276	360/360	115/115	203/215	236/239
5	Aurea	276/282	356/368	115/115	203/215	239/245
6	B-3	273/273	357/372	121/121	215/215	236/239
7	Barcelloner Zellernuss	273/276	360/360	121/121	212/215/203	236/239
8	Barcelona	273/282	357/357	115/115	215/215	236/239
9	Bergeri	273/276	357/360	121/121	215/215	239/239
10	Buttner's Zellernuss	273/273	357/357	121/121	215/215	236/236
11	Casina	273/273	368/368	115/115	215/215	239/239
12	Contorta	273/273	357/357	118/121	215/215	239/239
13	Cosford	273/273	372/372	121/121	203/203	236/239/245
14	Cutleaf	276/276	372/372	115/115	215/215	236/239
15	Des Anglais	273/276	357/357	121/121	215/215	236/239
16	DuChilly	273/276	361/361	121/121	203/215/212	236/239
17	Early Long Zeller	273/273	361/361	121/121	215/215	239/239
18	Fusco Rubra	282/282	358/369	121/121	215/215	239/239
19	Gasaway	276/276	357/372	115/115	203/215	239/245
20	Gem	276/282	360/360	115/121	215/215	236/239
21	Gunslebert	288/288	368/368	115/121	215/215	239/245
22	Gustav's Zellernuss	273/273	360/360	121/121	215/215	239/239
23	Hall's Giant	288/288	361/361	121/121	215/215	236/239
24	Iannusa Racinante	273/282/276	368/368	115/115	215/215	236/239
25	Imperiale de Trebizonde	273/273	361/369	121/121	215/215	236/242
26	Mortarella	273/273	368/369	115/115	215/215	239/239
27	Negret	273/273	357/357	115/121	215/215	239/239
28	OSU 026.072	282/284	361/361	115/124	212/212	239/242
29	OSU 054.039	282/282	361/368	118/118	203/212/215	239/239
30	OSU 408.040	276/282	361/372	121/121	215/215	239/245
31	OSU 495.049	282/282	361/369	115/121/124	212/212	236/239
32	OSU 556.027	273/273	361/368	115/121	212/212	236/239
33	OSU 681.078	282/282	360/372	121/121	215/215	239/239
34	OSU 759.010	273/273	361/368	115/115	203/203	236/236
35	Palaz	273/273	357/360/368	115/115	203/215/212	236/239
36	Pellicule Rouge	273/273	357/360	121/121	215/215	236/239
37	Pendula	273/273	372/372	121/121	215/215	239/245
38	Ratoli	273/282	356/356	115/121	215/215	239/239
39	Rode Zeller	273/273	361/372	121/121	203/203	236/239
40	Römische Nuss	273/273	356/360	121/121	215/215	239/239
41	Sant Jaume	273/282	357/357	115/115	215/215	239/239
42	Simon	273/273	360/360	115/115	215/215	236/239
43	Tapparona di SCC	273/282	357/372	115/121	215/215	239/245
44	Tombul Ghiaghli	273/273	361/368	118/118	203/215/212	239/239
45	Tonda Bianca	273/276/282	357/369	115/115	215/215	236/239/245
46	Tonda di Giffoni	273/273	357/369	115/115	203/215	236/239
47	Tonda G. d. Langhe	273/276	368/368	115/115	215/215	239/239
48	Tonda Romana	273/282	357/369	115/121	215/215	239/239
49	OSU 252.146	273/282	360/360	115/115	212/215	239/245
50	OSU 414.062	276/276	356/372	121/121	212/215	239/245

S4 Table (cont'd). Amplicon sizes at 18 tri-nucleotide repeat microsatellite loci developed from the 'Jefferson' hazelnut genome. Three or four amplicons were observed in some accessions.

No.	GB822	GB824	GB827	GB839	GB845	GB876
1	216/219	123/129/132	220/223	246/263	191/191	178/187
2	216/219	129/132/135	217/220/214	262/262	206/206	176/187
3	219/219	129/135	217/217	246/263	206/206	178/187
4	216/219	129/132	214/217	246/263	209/209	176/187
5	219/219	129/132/135	217/217	246/263	206/206	178/187
6	219/219	123/129/132	217/223	246/263	227/227	182/187
7	216/219	123/129/132	217/217	246/263	206/206	178/187/193
8	219/225	129/132	214/220	246/263	209/209	182/187/176
9	219/219	129/132/135	214/217	246/263	206/206	182/187
10	219/219	123/129/132	214/217	246/244	206/206	176/187/193
11	219/219	129/132	214/214	246/263	209/209	182/187
12	219/219	129/132	217/217	246/263	206/206	178/187/182/193
13	216/219	123/129/132	214/217	246/263	206/206	176/187
14	219/219	129/135	217/217	246/263	206/209	178/187
15	213/219	123/129/132	214/214	246/263	209/209	181/187/176/178
16	216/219	129/132/135	214/214	246/263	209/209	182/187
17	213/219	129/132/135	217/217	246/263/250	206/206	187/193
18	213/219	123/129/132	217/217	246/263	206/224	178/187
19	219/219	129/132/135	217/217	246/263	209/209	178/185
20	219/219	129/132	214/214	246/263	209/209	182/187
21	219/219	129/132	217/220	246/263	209/209	187/193
22	213/219	129/132/135	217/217	246/263/250	206/231	187/190/193
23	219/219	129/132	217/217	246/263	206/206	187/190/193
24	216/219	129/132/135	214/220	246/263	191/206	178/187
25	218/219	129/132	214/223	246/263	209/209	182/187
26	219/219	129/132/135	214/220	246/263	203/206	183/187
27	216/219	129/132	214/214	246/263	209/209	183/187
28	216/219	129/132	217/220	246/263	209/209	182/187
29	219/219	123/129/132/135	223/223	263/263	191/191	183/188
30	216/219	129/132	220/220	246/263	209/209	178/187
31	219/219	129/132	220/220	246/263	206/206	182/187
32	219/219	129/132	214/223	246/263	209/209	184/187/178
33	213/219/216	123/129/132/135	217/217	246/263	206/224	177/187
34	216/219	129/135	220/223	246/263	209/209	182/187
35	218/219/216	123/129/132/135	214/223	246/263	209/209	183/187
36	218/219	129/132/135	214/214	246/263	206/209	182/187/193
37	219/219	123/129/132/135	217/217	246/263	209/209	178/187
38	219/219	129/132	214/217	246/263	209/209	182/187
39	219/219	123/129/132	214/220	246/263	209/209	182/187/176
40	219/219	123/129/132/135	214/220	246/263	206/209	182/187
41	213/219	129/132	214/214	246/263	206/209	176/187
42	219/225	129/132	214/214	246/263	209/209	182/187
43	219/219	129/132	214/217	246/244	206/206	182/187/178
44	219/219	129/132/135	214/223	246/263	209/209	182/187
45	219/219	129/132/135	217/220	246/263	206/224	187/187
46	219/219	129/135	214/220	246/263	206/206	182/187
47	219/219	129/132	214/220	246/263	209/224/206	178/187
48	213/219	129/132	214/214	246/263	209/209	182/187/177
49	216/219	129/132	214/220	246/263	209/209	176/182/187
50	219/225	129/132/135	214/223	246/263	209/209	178/182/187/190

S4 Table (cont'd). Amplicon sizes at 18 tri-nucleotide repeat microsatellite loci developed from the 'Jefferson' hazelnut genome. Three or four amplicons were observed in some accessions.

No.	GB903	GB910	GB915	GB918	GB922	GB926
1	124/127/123	308/308	265/268	299/305	195/199	252/261
2	124/127	311/315	265/265	299/305	199/206/195	252/252
3	124/127/123	308/308	265/268	305/311	199/202/195	258/261
4	123/127	308/311	262/268	305/305	199/202/195	252/252
5	123/127	308/311	262/265	305/305	199/202/195	252/258
6	124/127	308/308	265/268	302/305	195/199	261/261
7	124/127	308/311	262/265	299/305	195/199	252/255
8	124/127	308/311	262/265	305/305	199/205/195	252/258
9	124/127	308/308	262/262	302/305	199/205/195	258/258
10	124/127/123	308/308	262/265	299/305	199/202/195	258/258
11	123/127	311/311	262/262	302/305	195/205/206	258/261
12	124/127	308/308	262/268	305/305	195/199/202	258/258
13	127/127	308/311	262/262	299/305	199/202/195	252/255
14	124/127	308/311	262/262	302/305/311	199/202/195	252/258
15	124/127/123	308/308	265/268	305/305	195/205/206	252/261
16	124/127	308/308	262/265	305/305	199/205/195	252/261
17	124/124/123	308/315/311	262/268	302/305	195/199	258/258
18	124/127/123	308/315	262/265	299/305	195/199	252/255
19	124/127	311/311	265/265	305/305	195/199	249/258
20	124/127	308/311	265/265	305/305	195/205	252/252
21	123/127	308/308	262/262	305/305	195/199	252/258
22	124/127/123	315/317	262/268	305/305	199/202/195	258/258
23	124/127	308/317	262/265	305/305	199/202/195	258/261
24	124/124	311/317	263/268	305/305	195/205	252/258
25	127/127	308/311	262/265	305/305	199/205/195	258/261/252
26	124/127	308/311	262/268	305/311	199/202/195	252/258
27	123/127	308/311	262/268	305/305	195/199	252/258
28	124/133/123/127	308/311	262/271	299/305	195/199	252/258
29	127/127	308/308	262/268	305/311	199/205/195	258/258
30	124/127	308/308	262/265	305/311	199/202/195	252/258
31	127/127	308/311	262/262	305/305	195/205	240/240
32	127/133	308/308	262/265	299/305	195/199/205	258/258
33	127/133/123	308/308	265/268	305/305	199/206	252/252
34	127/127	311/311	262/265	299/305	195/199	258/258
35	127/127	308/311	265/268	305/305	199/205/195	258/258
36	124/127	308/308	262/268/265	305/305	199/205/195	258/261
37	124/127	311/311	262/271	299/305	195/199	252/258
38	127/127	311/311	266/268	305/305	195/199	252/258
39	127/127	308/315	262/262	299/305	195/199	252/258
40	123/127	308/311	262/262	305/305	199/202/195/205	252/258
41	124/127	311/311	265/266	305/305	195/199	252/258
42	124/127	308/311	262/268	325/325	199/202/195	252/252
43	124/127/123	308/311	262/265	305/305	195/202/205	258/258
44	127/127	308/311	262/262	299/305	195/205/199	252/258
45	124/127/123	308/308	262/262	305/305	195/199	249/252
46	124/127/123	308/311	262/262	305/305	199/205/195	252/258
47	127/127	311/311	262/268	305/305	199/202/195	252/258
48	127/127	308/311	262/265	302/305	199/205/195	249/258
49	124/124	311/311	262/265	305/305	195/199	252/252
50	124/127	308/311	262/265	299/305	195/199/205	249/258

S4 Table (cont'd). Amplicon sizes at 18 tri-nucleotide repeat microsatellite loci developed from the 'Jefferson' hazelnut genome. Three or four amplicons were observed in some accessions.

No.	GB929
1	278/278
2	278/278
3	278/278
4	278/278
5	278/278
6	272/278
7	272/278
8	278/278
9	272/272
10	272/272
11	278/278
12	278/278
13	272/272
14	278/278
15	278/278
16	272/278
17	272/278
18	278/278
19	275/281/278
20	272/278
21	272/278
22	272/278
23	272/272
24	278/278
25	278/278
26	278/278
27	278/278
28	278/278
29	278/278
30	278/278
31	278/281
32	278/278
33	275/278/281
34	275/278
35	278/278
36	278/278
37	278/281
38	278/278
39	272/278
40	278/278
41	278/278
42	278/278
43	278/278
44	278/278
45	278/278
46	278/278
47	272/278
48	278/278
49	275/278
50	275/278
